# Supplementary material for: A complete, multi-level conformational clustering of antibody complementarity-determining regions
Source: PeerJ. 2014 Jul 1;2:e456. doi: 10.7717/peerj.456 (PMC4103072; doi:10.7717/peerj.456)
Supplement: Supplemental Information 1 — Rogue CDR sequences in every CDR/length, with the respective lists of level-1 cluster-tagged CDRs, in which they are identified. Entries with an asterisk indicate bound structures. Additionally, entries with completely identical Fvs that belong to different conformational clusters (〈***full-chain rogues〉), are given immediately after the detected rogue CDR sequences, when applicable. [file peerj-02-456-s001.doc]

**Rogue CDR sequences in every CDR/length, with the respective lists of level-1 cluster-tagged CDRs, in which they are identified. Entries with an asterisk indicate bound structures. Additionally, entries with completely identical Fv that belong to different conformational clusters (<***full-chain rogues>), are given immediately after the detected rogue CDR sequences, when applicable.**

--------------------------------------

clusters_H1_13res

VASGFTFNNYWMS

[3GHE_H*->I, 1aif_H->II, 1aif_B->II, 1iai_I*->II, 3IY5_B->II]

****Full-chain rogues:

none

KASGYTFTSYWMH

[1a6u_H->I, 1a6v_H*->I, 1a6v_I*->I, 1a6v_J*->I, 1a6w_H*->I, 1fbi_H*->I, 1fbi_Q*->I, 1n7m_L*->I, 1ngp_H*->I, 1ngq_H->I, 1ngw_H*->I, 1ngw_B*->I, 1ngy_B->I, 1ngz_B->I, 1nqb_A->I, 1nqb_C->I, 1oaq_H->I, 1oar_H*->I, 1oar_I*->I, 1oar_J*->I, 1oau_H*->I, 1oau_J*->I, 1oax_H*->I, 1oax_J*->I, 1oay_H*->I, 1oay_J*->I, 1oaz_H*->I, 1oaz_J*->I, 1wt5_A->I, 1wt5_B->I, 1z3g_H*->I, 1z3g_I*->I, 2bjm_H*->I, 2XZQ_H*->I, 2y06_H*->I, 2Y07_H*->I, 2Y36_H*->I, 3CMO_H->I, 3CMO_Y->I, 3fct_B*->I, 3fct_D*->I, 1ngx_B*->XI, 1ngx_H*->XI]

****Full-chain rogues:

[1n7m_L*->I, 1ngz_B->I, 1ngx_B*->XI, 1ngx_H*->XI]

AASRRSSRSWAMA

[3QXW_D->II, 3QXT_A*->IV, 3QXT_B*->IV, 3QXV_A*->IV, 3QXV_B*->IV, 3QXV_C*->IV, 3QXV_D*->IV, 3QXV_E*->IV]

****Full-chain rogues:

[3QXW_D->II, 3QXT_A*->IV, 3QXT_B*->IV, 3QXV_A*->IV, 3QXV_B*->IV, 3QXV_C*->IV, 3QXV_D*->IV, 3QXV_E*->IV]

AVSGSTYSPCTTG

[1YC7_A->IV, 1YC8_B->V, 1YZZ_B->V]

****Full-chain rogues:

none

--------------------------------------

clusters_H2_10res

AINWDSARTY

[1hcv_A->I, 1G9E_A_13->II, 1G9E_A_4->V, 1G9E_A_9->V, 1G9E_A_10->V, 1G9E_A_11->V, 1G9E_A_18->V, 1G9E_A_20->V]

****Full-chain rogues:

[1G9E_A_13->II, 1G9E_A_4->V, 1G9E_A_9->V, 1G9E_A_10->V, 1G9E_A_11->V, 1G9E_A_18->V, 1G9E_A_20->V]

EILPGSGSTN

[1bql_H*->I, 2iff_H*->I, 2V7H_B->I, 2V7H_H->I, 2ZJS_H*->I, 3hfl_H->I, 1d6v_H*->V]

****Full-chain rogues:

none

YISSGGGSTY

[2ZUQ_C*->II, 2ZUQ_F*->II, 2KH2_B*->III]

****Full-chain rogues:

none

AVSGSGGSTY

[1OL0_A->II, 1OL0_B->II, 1vhp_A->IV]

****Full-chain rogues:

none

AMDSGGGGTL

[2P42_B*->II, 2P42_D*->II, 2P43_B*->II, 2P44_B*->II, 2P45_B*->II, 2P46_B*->II, 2P46_D*->II, 2P47_B*->II, 2P48_B*->II, 2P49_B*->II, 2P4A_B*->II, 2P4A_D*->II, 3QSK_B*->II, 1bzq_K*->VI, 1bzq_L*->VI, 1bzq_M*->VI, 1bzq_N*->VI]

****Full-chain rogues:

[2P42_B*->II, 2P42_D*->II, 2P43_B*->II, 2P44_B*->II, 2P45_B*->II, 2P46_B*->II, 2P46_D*->II, 2P47_B*->II, 2P48_B*->II, 2P49_B*->II, 2P4A_B*->II, 2P4A_D*->II, 1bzq_K*->VI, 1bzq_L*->VI, 1bzq_M*->VI, 1bzq_N*->VI]

GITPAGGYTY

[2fjf_H->II, 2fjf_B->II, 2fjf_D->II, 2fjf_F->II, 2fjf_I->II, 2fjf_K->II, 2fjf_N->II, 2fjf_P->II, 2fjf_R->II, 2fjf_T->II, 2fjf_V->II, 2fjf_X->II, 2fjg_H*->IX, 2fjg_B*->IX]

****Full-chain rogues:

[2fjf_H->II, 2fjf_B->II, 2fjf_D->II, 2fjf_F->II, 2fjf_I->II, 2fjf_K->II, 2fjf_N->II, 2fjf_P->II, 2fjf_R->II, 2fjf_T->II, 2fjf_V->II, 2fjf_X->II, 2fjg_H*->IX, 2fjg_B*->IX]

--------------------------------------

clusters_H2_12res

FIGNKANDYTTE

[3I50_H*->I, 3IXX_G*->III, 3IXX_I*->III, 3IXY_G*->III, 3IXY_I*->III]

****Full-chain rogues:

none

--------------------------------------

clusters_H2_9res

SISSPGTIY

[1YC8_A->I, 1YC8_B->I, 1YZZ_A->I, 1YZZ_B->I, 1YC7_A->V, 1YC7_B->V]

****Full-chain rogues:

none

NVYDSGDTN

[2aj3_B->I, 2aj3_D->VI, 2aj3_F->VI]

****Full-chain rogues:

[2aj3_B->I, 2aj3_D->VI, 2aj3_F->VI]

--------------------------------------

clusters_H3_5res

GLFDY

[1p4b_H*->I, 1p4i_H->II]

****Full-chain rogues:

[1p4b_H*->I, 1p4i_H->II]

RDSDY

[1n7m_L*->I, 1ngx_B*->IV, 1ngx_H*->IV]

****Full-chain rogues:

[1n7m_L*->I, 1ngx_B*->IV, 1ngx_H*->IV]

EGYIY

[1ggi_H*->I, 1ggi_J*->I, 1ggb_H->V, 1ggc_H->V]

****Full-chain rogues:

[1ggi_H*->I, 1ggi_J*->I, 1ggb_H->V, 1ggc_H->V]

--------------------------------------

clusters_H3_6res

EYDEAY

[2EH8_H*->I, 2EH7_H->II]

****Full-chain rogues:

[2EH8_H*->I, 2EH7_H->II]

--------------------------------------

clusters_H3_9res

HGGYYAMDY

[1igt_D->III, 1igt_B->IV]

****Full-chain rogues:

[1igt_D->III, 1igt_B->IV]

--------------------------------------

clusters_H3_10res

GDYYGSRGAY

[2gfb_B->VI, 2gfb_D->VI, 2gfb_F->VI, 2gfb_H->VI, 2gfb_J->VI, 2gfb_L->VI, 2gfb_N->VI, 2gfb_P->VI, 1kno_B*->XXVIII, 1kno_D*->XXVIII, 1kno_F*->XXVIII]

****Full-chain rogues:

[2gfb_B->VI, 2gfb_D->VI, 2gfb_F->VI, 2gfb_H->VI, 2gfb_J->VI, 2gfb_L->VI, 2gfb_N->VI, 2gfb_P->VI, 1kno_B*->XXVIII, 1kno_D*->XXVIII, 1kno_F*->XXVIII]

--------------------------------------

clusters_H3_11res

EKTTYYYAMDY

[1ad9_H->II, 1ad9_B->II, 1ae6_H->III]

****Full-chain rogues:

none

YDYYGGSYFDY

[2XZQ_H*->II, 2Y36_H*->II, 2Y07_H*->III]

****Full-chain rogues:

none

DRHDYGEIFTY

[1jp5_A*->XII, 1jp5_B*->XII, 1svz_A*->XII, 1svz_B*->XII, 1n4x_H->XXI, 1n4x_I->XXI]

****Full-chain rogues:

none

--------------------------------------

clusters_H3_13res

HWGGYYIPYGMDH

[2GK0_H->VI, 3FO0_H->VI, 3FO1_H*->VI, 3FO1_B*->VI, 3FO2_H*->VI, 3FO2_B*->VI, 2GJZ_H->XX, 2GJZ_B->XX]

****Full-chain rogues:

[2GK0_H->VI, 3FO0_H->VI, 2GJZ_H->XX, 2GJZ_B->XX, 3FO1_H*->VI, 3FO1_B*->VI, 3FO2_H*->VI, 3FO2_B*->VI]

--------------------------------------

clusters_H3_14res

KGSDRLSDNDPFDA

[1op3_H*->II, 1op3_M*->II, 1op5_H*->II, 1op5_M*->II, 1zls_H*->II, 1zlu_H*->II, 1zlu_M*->II, 1zlv_M*->II, 1zlw_H*->II, 1zlw_M*->II, 2OQJ_B*->II, 2OQJ_E*->II, 2OQJ_H*->II, 2OQJ_K*->II, 3OAU_H*->II, 3OAY_M*->II, 3OAY_H*->II, 3OAZ_M->II, 3OB0_H*->II, 3OB0_M*->II, 1om3_K->X]

****Full-chain rogues:

[1op3_H*->II, 1op3_M*->II, 1op5_H*->II, 1op5_M*->II, 1zls_H*->II, 1zlu_H*->II, 1zlu_M*->II, 1zlv_M*->II, 1zlw_H*->II, 1zlw_M*->II, 2OQJ_B*->II, 2OQJ_E*->II, 2OQJ_H*->II, 2OQJ_K*->II, 3OAU_H*->II, 3OAY_M*->II, 3OAY_H*->II, 3OAZ_M->II, 3OB0_H*->II, 3OB0_M*->II, 1om3_K->X]

--------------------------------------

clusters_H3_15res

LYLFEGAQSSNAFDL

[3MLR_H*->II, 3MLS_H*->II, 3MLS_I*->II, 3MLS_J*->II, 3MLS_K*->II, 3MLT_H*->II, 3MLT_B*->II, 3MLU_H*->II, 3MLV_H*->II, 3MLV_N*->II, 3MLT_E*->XIV, 3MLT_I*->XIV]

****Full-chain rogues:

[3MLR_H*->II, 3MLS_H*->II, 3MLS_I*->II, 3MLS_J*->II, 3MLS_K*->II, 3MLT_H*->II, 3MLT_B*->II, 3MLU_H*->II, 3MLV_H*->II, 3MLV_N*->II, 3MLT_E*->XIV, 3MLT_I*->XIV]

PWYPFMASKGSEFDY

[2VYR_I*->V, 2VYR_J*->V, 2VYR_K*->V, 2VYR_L*->V, 2VYR_E*->VII, 2VYR_F*->VII, 2VYR_G*->VII, 2VYR_H*->VII]

****Full-chain rogues:

[2VYR_I*->V, 2VYR_J*->V, 2VYR_K*->V, 2VYR_L*->V, 2VYR_E*->VII, 2VYR_F*->VII, 2VYR_G*->VII, 2VYR_H*->VII]

RGFYGRKYEVNHFDY

[3BAE_H*->IX, 3BKC_H->IX, 3BKJ_H*->IX, 3BKM_H->IX, 3AAZ_A->XVI, 3AAZ_H->XVI]

****Full-chain rogues:

none

--------------------------------------

clusters_H3_16res

EGPRATGYSMADVFDI

[3CSY_A*->II, 3CSY_C*->II, 3CSY_E*->II, 3CSY_G*->II, 3INU_H->V, 3INU_M->V]

****Full-chain rogues:

[3CSY_A*->II, 3CSY_C*->II, 3CSY_E*->II, 3CSY_G*->II, 3INU_H->V, 3INU_M->V]

--------------------------------------

clusters_L1_14res

TGTSSDVGGYNYVS

[1a8j_L*->II, 1mcb_A*->II, 1mcb_B*->II, 1mcc_A*->II, 1MCD_A*->II, 1mce_A*->II, 1mcf_A*->II, 1MCH_A*->II, 1mci_A*->II, 1MCJ_A*->II, 1mck_A*->II, 1mcl_A*->II, 1mcn_A*->II, 1mco_L->II, 1mcq_A*->II, 1mcr_A*->II, 1mcs_A*->II, 1mcw_M->II, 2mcg_2->II, 3KDM_L*->II, 3KDM_A*->II, 3mcg_1->II, 3mcg_2->II, 1mcc_B*->VII, 1mcn_B*->VII, 1mcs_B*->VII]

****Full-chain rogues:

[1mcb_A*->II, 1mcb_B*->II, 1mcc_A*->II, 1MCD_A*->II, 1mce_A*->II, 1mcf_A*->II, 1MCH_A*->II, 1mci_A*->II, 1MCJ_A*->II, 1mck_A*->II, 1mcl_A*->II, 1mcn_A*->II, 1mcq_A*->II, 1mcr_A*->II, 1mcs_A*->II, 1mcw_M->II, 2mcg_2->II, 3mcg_1->II, 3mcg_2->II, 1mcc_B*->VII, 1mcn_B*->VII, 1mcs_B*->VII, 1mco_L->II]

TGVSSIVGSYNLVS

[1jvk_B->III, 1lgv_B->III, 1lhz_B->III, 1jvk_A->IV, 1lgv_A->IV, 1lhz_A->IV]

****Full-chain rogues:

[1jvk_B->III, 1lgv_B->III, 1lhz_B->III, 1jvk_A->IV, 1lgv_A->IV, 1lhz_A->IV]

--------------------------------------

clusters_L2_7res

GDNNRPS

[3MAC_L*->I, 3MA9_L*->II]

****Full-chain rogues:

[3MAC_L*->I, 3MA9_L*->II]

GNNERPS

[3LZF_L*->I, 3QHZ_M->I, 3QHF_L->II]

****Full-chain rogues:

none

YTSRLHS

[1dvf_C*->I, 1fai_L->I, 1fbi_L*->I, 1fbi_P*->I, 1fe8_L*->I, 1fe8_M*->I, 1fe8_N*->I, 1ghf_L->I, 1jv5_A->I, 1mam_L->I, 1xiw_C*->I, 1xiw_G*->I, 1ynt_A*->I, 1ynt_C*->I, 2f19_L->I, 2XKN_C->I, 3CFD_L*->I, 3CFD_A*->I, 3CFE_L->I, 3CFE_A->I, 3HNS_L*->I, 3HNT_L*->I, 3HNV_L*->I, 2a6d_L*->III, 2a6d_A*->III, 2a6i_A*->III, 2a6j_L->III, 2a6j_A->III, 2a6k_A*->III, 2a6k_L*->III, 2ZJS_L*->III]

****Full-chain rogues:

None

YTSNLHS

[1a14_L*->I, 1nma_L*->I, 1nmb_L*->I, 1nmc_L*->I, 1nmc_C*->I, 2V7H_A->III, 2V7H_L->III]

****Full-chain rogues:

none

FTSRSQS

[1jfq_L->I, 6fab_L->III]

****Full-chain rogues:

[1jfq_L->I, 6fab_L->III]

--------------------------------------

clusters_L3_10res

SSYEGSDNFV

[1dcl_B->II, 1mcb_A*->II, 1mcc_A*->II, 1MCD_A*->II, 1mce_A*->II, 1mcf_A*->II, 1MCH_A*->II, 1mci_A*->II, 1MCJ_A*->II, 1mck_A*->II, 1mcl_A*->II, 1mcn_A*->II, 1mco_L->II, 1mcq_A*->II, 1mcr_A*->II, 2mcg_2->II, 3mcg_2->II, 1mcf_B*->II, 1mcs_A*->VII, 1mcw_M->VII, 1a8j_H*->XII, 1mcb_B*->XII, 1mcc_B*->XII, 1MCD_B*->XII, 1mce_B*->XII, 1MCH_B*->XII, 1mci_B*->XII, 2mcg_1->XII, 1MCJ_B*->XII, 1mck_B*->XII, 1mcl_B*->XII, 1mcn_B*->XII, 1mcq_B*->XII, 1mcr_B*->XII, 1mcs_B*->XII]

****Full-chain rogues:

[1mcb_A*->II, 1mcc_A*->II, 1MCD_A*->II, 1mce_A*->II, 1mcf_A*->II, 1MCH_A*->II, 1mci_A*->II, 1MCJ_A*->II, 1mck_A*->II, 1mcl_A*->II, 1mcn_A*->II, 1mcq_A*->II, 1mcr_A*->II, 2mcg_2->II, 3mcg_2->II, 1mcf_B*->II, 1mcs_A*->VII, 1mcw_M->VII, 1a8j_H*->XII, 1mcb_B*->XII, 1mcc_B*->XII, 1MCD_B*->XII, 1mce_B*->XII, 1MCH_B*->XII, 1mci_B*->XII, 2mcg_1->XII, 1MCJ_B*->XII, 1mck_B*->XII, 1mcl_B*->XII, 1mcn_B*->XII, 1mcq_B*->XII, 1mcr_B*->XII, 1mcs_B*->XII, 1mco_L->II]

QVWDSNASVV

[1lil_B->II, 1lil_A->XII]

****Full-chain rogues:

[1lil_B->II, 1lil_A->XII]

--------------------------------------

clusters_L3_11res

AAWDDSLDVAV

[1bjm_B->I, 3bjl_A->I, 3bjl_B->I, 4bjl_B->I, 4bjl_A->V]

****Full-chain rogues:

[1bjm_B->I, 3bjl_A->I, 3bjl_B->I, 4bjl_B->I, 4bjl_A->V]

--------------------------------------

clusters_L3_13res

GVGDTIKEQFVYV

[2OTU_A*->I, 2OTU_C*->I, 2OTU_E*->I, 2OTU_G*->I, 2OTW_A*->I, 2OTW_C*->I, 2GSG_A->II, 2QHR_L*->II, 3FFD_B*->II]

****Full-chain rogues:

[2OTU_A*->I, 2OTU_C*->I, 2OTU_E*->I, 2OTU_G*->I, 2OTW_A*->I, 2OTW_C*->I, 2GSG_A->II]

--------------------------------------

clusters_L3_8res

YQYNNGYT

[1fn4_A->I, 1fn4_C->I, 1F3R_B*->V]

****Full-chain rogues:

[1fn4_A->I, 1fn4_C->I, 1F3R_B*->V]

FQGSLVPT

[1keg_L*->II, 1ehl_L*->III]

****Full-chain rogues:

[1keg_L*->II, 1ehl_L*->III]

QHSRELLT

[3DGG_C->II, 3DGG_A->V]

****Full-chain rogues:

[3DGG_C->II, 3DGG_A->V]

--------------------------------------

clusters_L3_9res

KQSYYHMYT

[3BD3_B->I, 3BD4_A->I, 3BD5_A->I, 3BD5_B->I, 2GKI_A->III, 2GKI_B->III]

****Full-chain rogues:

none

QQWSSHIFT

[3C09_L*->II, 3C09_B*->II, 3C08_L->III]

****Full-chain rogues:

[3C09_L*->II, 3C09_B*->II, 3C08_L->III]

QVYGASSYT

[3JUY_B->III, 3JUY_A->III, 3JUY_C->III, 3JUY_E->III, 3JUY_F->III, 3JUY_D->III, 1hzh_L->V, 1hzh_M->V, 1n0x_L*->V, 1n0x_M*->V, 2NY7_L*->V, 3RU8_L*->V]

****Full-chain rogues:

none
