# Supplementary material for: A complete, multi-level conformational clustering of antibody complementarity-determining regions
Source: PeerJ. 2014 Jul 1;2:e456. doi: 10.7717/peerj.456 (PMC4103072; doi:10.7717/peerj.456)
Supplement: Supplemental Information 2 — The cluster medoid/median or representative of the external sets was used for identification of correspondences. Only level-1 clusters with a correspondence are shown here, in order to preserve a readable size for the table (213 total level-1 clusters in CDR-H3). In brackets, next to each correspondence, is the full, level-3, classification in this work of the representative of the external set. The entire correspondence is marked between square brackets and in full-italics because the CDR-H3 definition used in North, Lehmann & Dunbrack (2011), was longer by 2 residues (i.e., 93-102). [file peerj-02-456-s002.doc]

**Supplementary Table:** Comparison of level-1 conformational clusters obtained in CDR-H3 with North et al., 2011. The cluster medoid/median or representative of the external sets was used for identification of correspondences. Only level-1 clusters with a correspondence are shown here, in order to preserve a readable size for the table (213 total level-1 clusters in CDR-H3). In brackets, next to each correspondence, is the full, level-3, classification in this work of the representative of the external set. The entire correspondence is marked between square brackets and in full-italics because the CDR-H3 definition used in North et al., 2011, was longer by 2 residues (*i.e.* 93-102).
