# Supplementary material for: A complete, multi-level conformational clustering of antibody complementarity-determining regions
Source: PeerJ. 2014 Jul 1;2:e456. doi: 10.7717/peerj.456 (PMC4103072; doi:10.7717/peerj.456)
Supplement: Supplemental Information 3 — Collection of heatmaps for all CDR/length combinations, showing the minimum number of amino acid differences, position-by-position, between any two sequences of different clusters. allow a quick visual appreciation of the degree of sequence dissimilarity between clusters. mSD heatmaps allow a quick visual appreciation of the degree of sequence dissimilarity between clusters. [file peerj-02-456-s003.pdf]

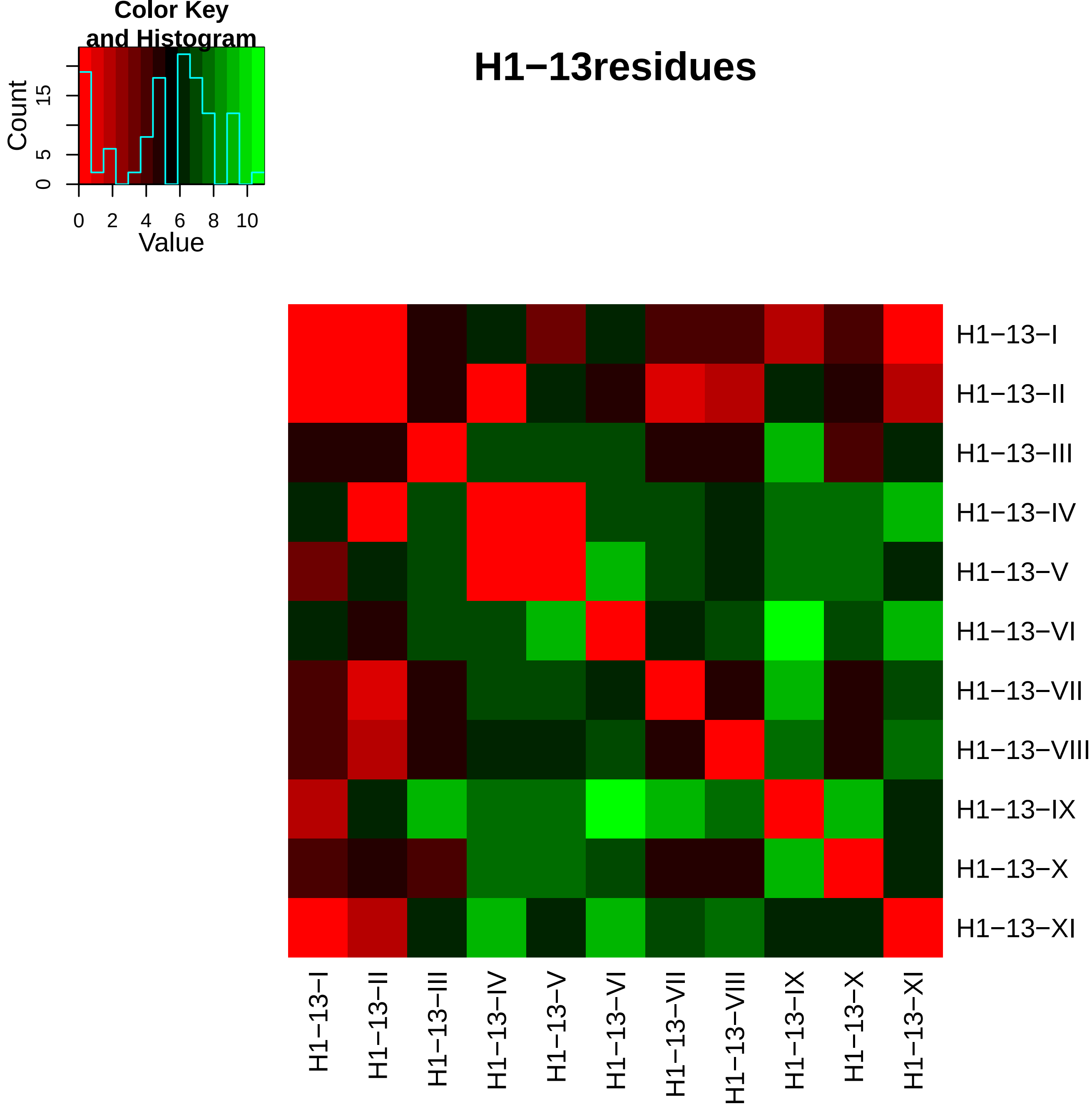
Color Key
and Histogram
Count
Value
0
5
15
0
2
4
6
8
10
H1−13residues
H1−13−I
H1−13−II
H1−13−III
H1−13−IV
H1−13−V
H1−13−VI
H1−13−VII
H1−13−VIII
H1−13−IX
H1−13−X
H1−13−XI

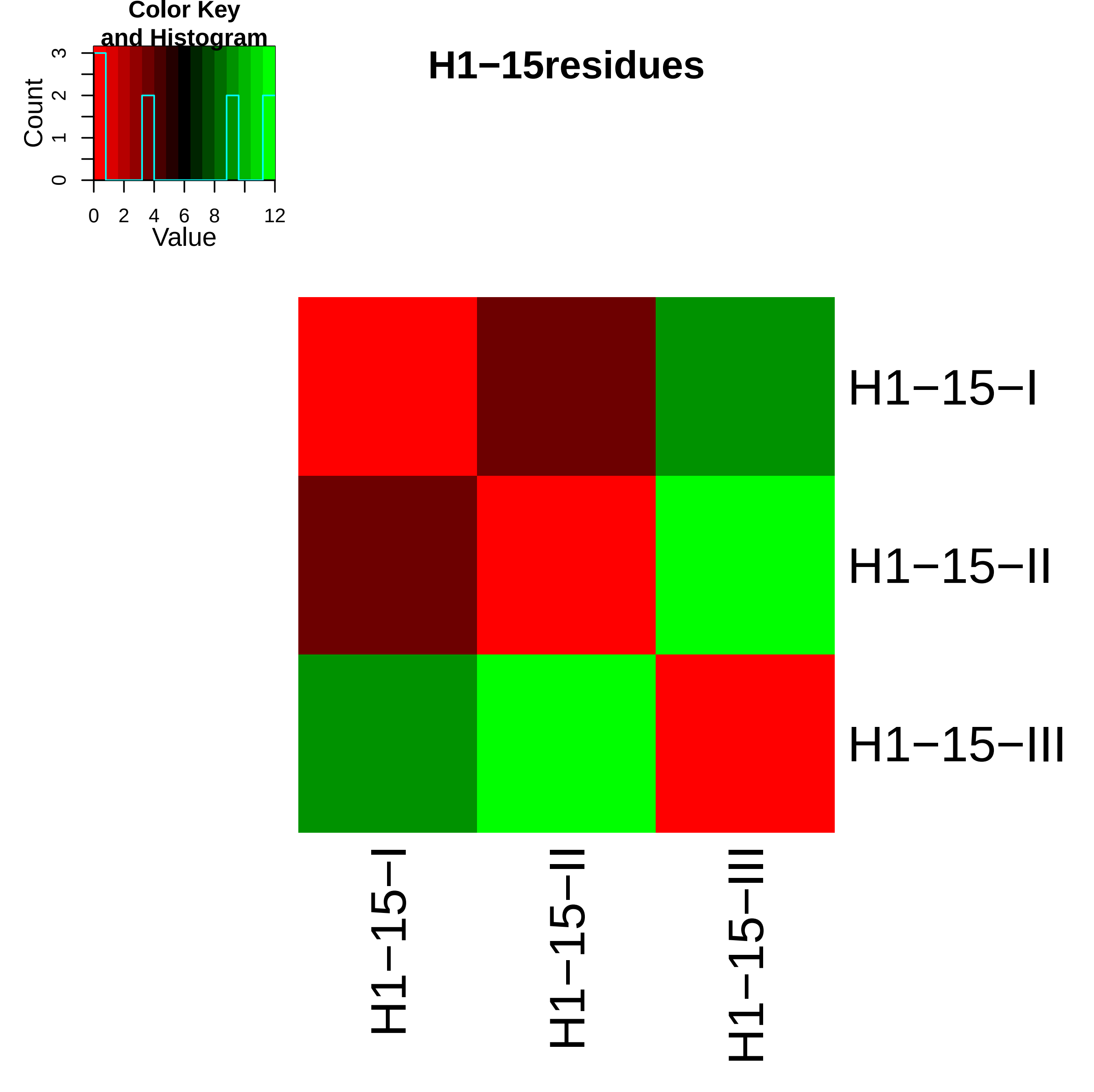

Color Key
and Histogram
Count
0
1
2
3
0
2
4
6
8
12
Value
H1−15residues
H1−15−I
H1−15−II
H1−15−III
H1−15−I
H1−15−II
H1−15−III

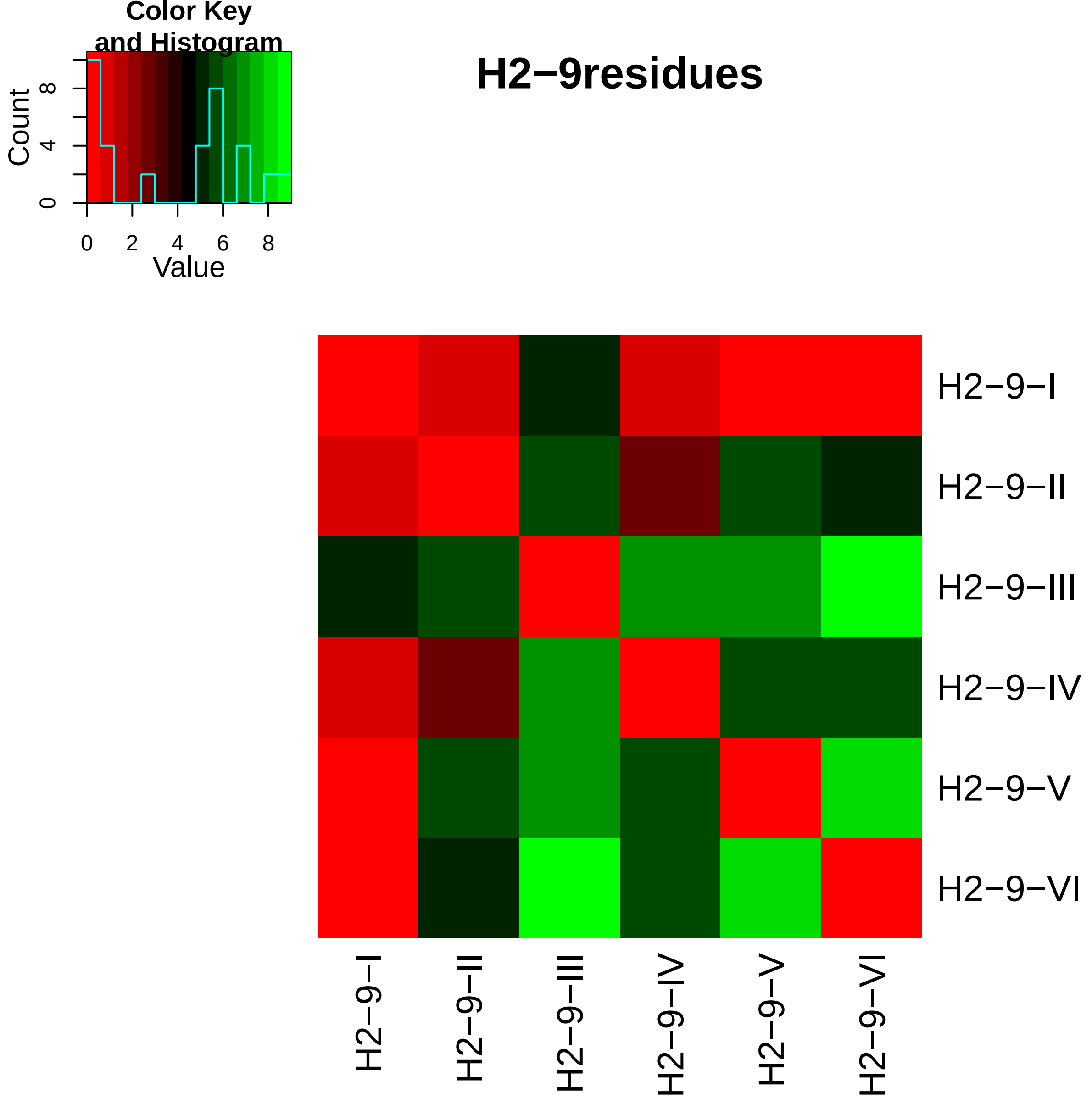

Color Key
and Histogram
Count
Value
0
4
8
0
2
4
6
8
H2−9residues
H2−9−I
H2−9−II
H2−9−III
H2−9−IV
H2−9−V
H2−9−VI

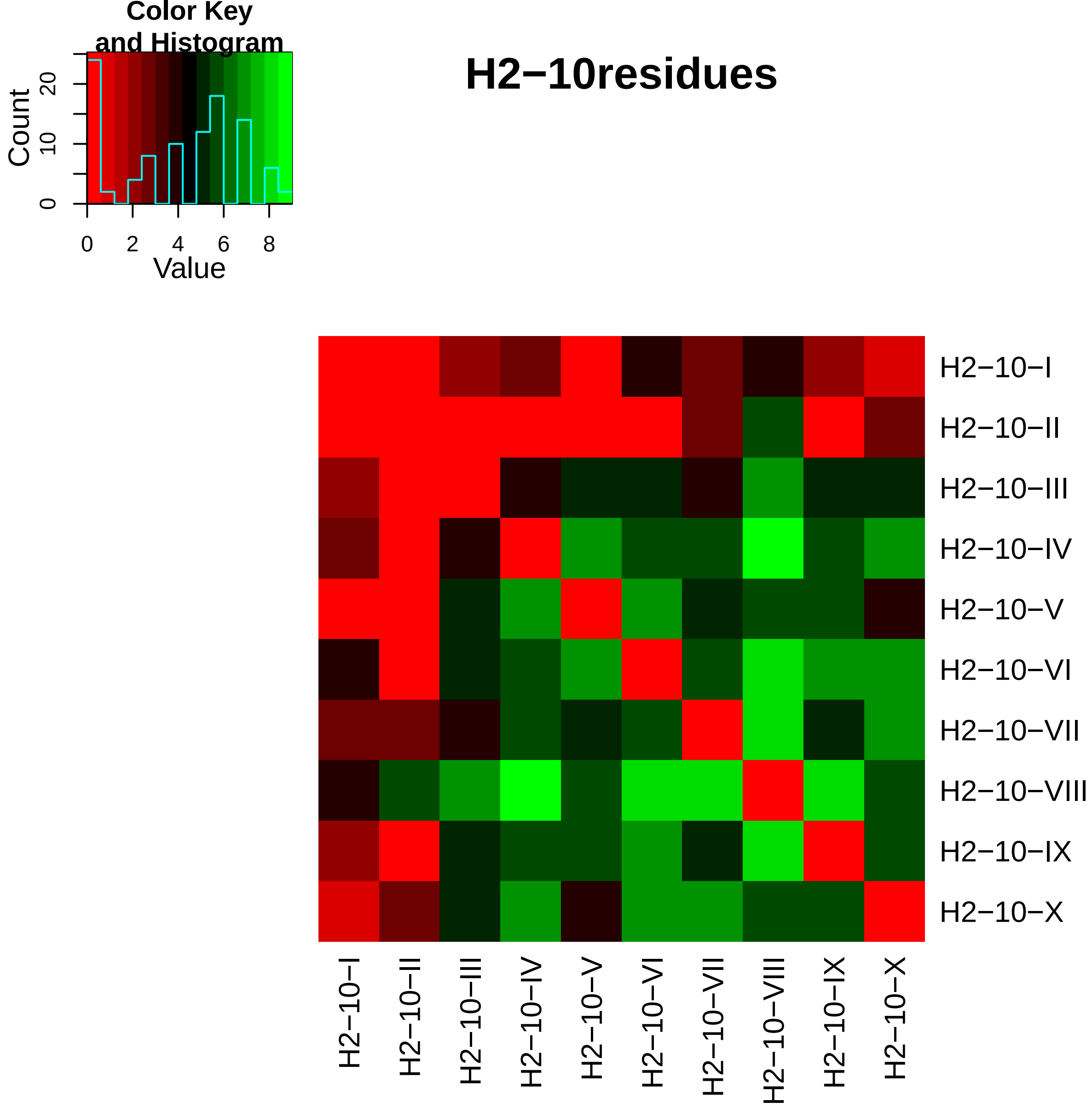
H2−10residues
Color Key
and Histogram
Count
Value
0
10
20
0
2
4
6
8
H2−10−I
H2−10−II
H2−10−III
H2−10−IV
H2−10−V
H2−10−VI
H2−10−VII
H2−10−VIII
H2−10−IX
H2−10−X

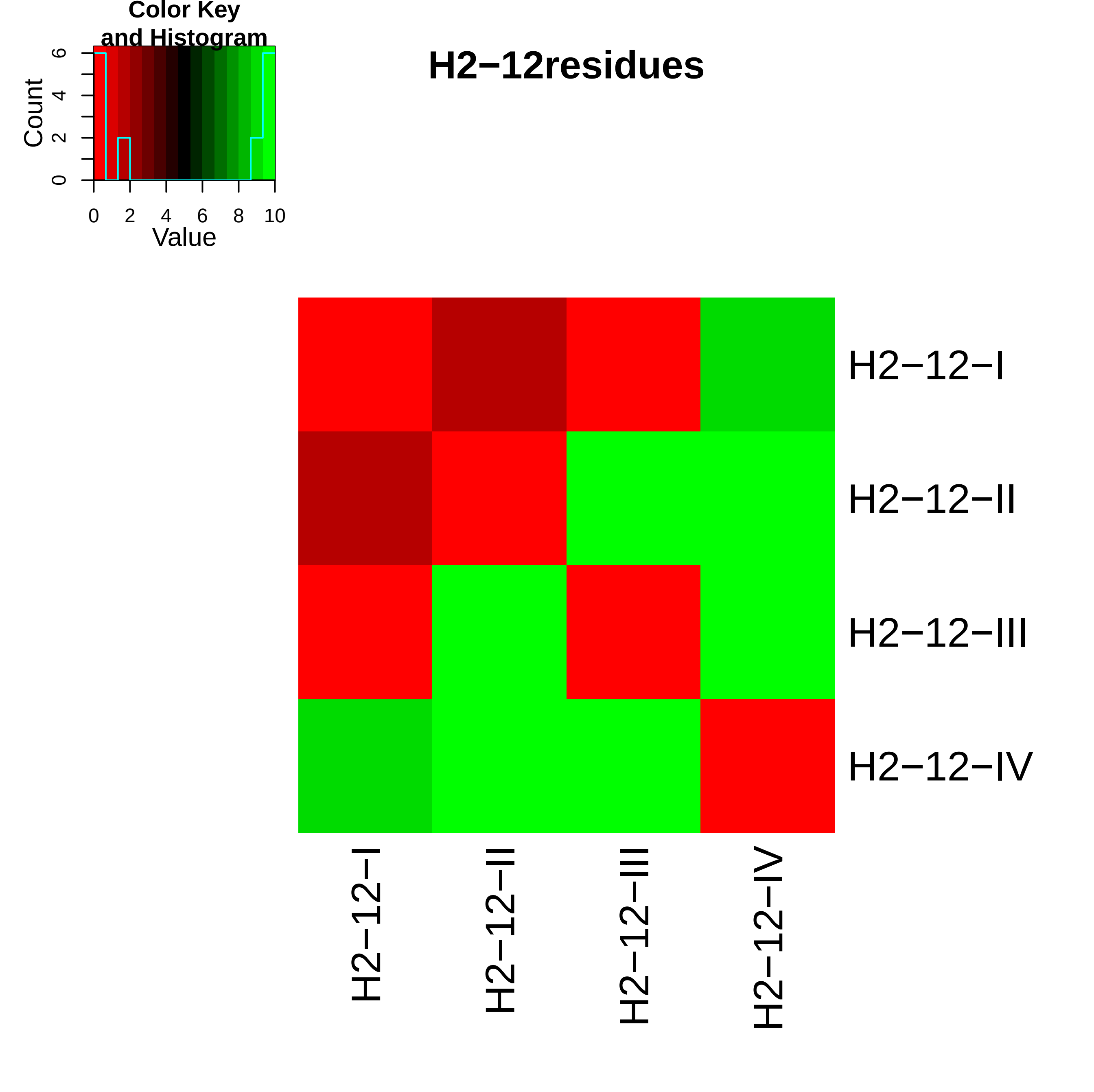
Color Key
and Histogram
Count
Value
0
2
4
6
0
2
4
6
8
10
H2−12residues
H2−12−I
H2−12−II
H2−12−III
H2−12−IV
H2−12−I
H2−12−II
H2−12−III
H2−12−IV

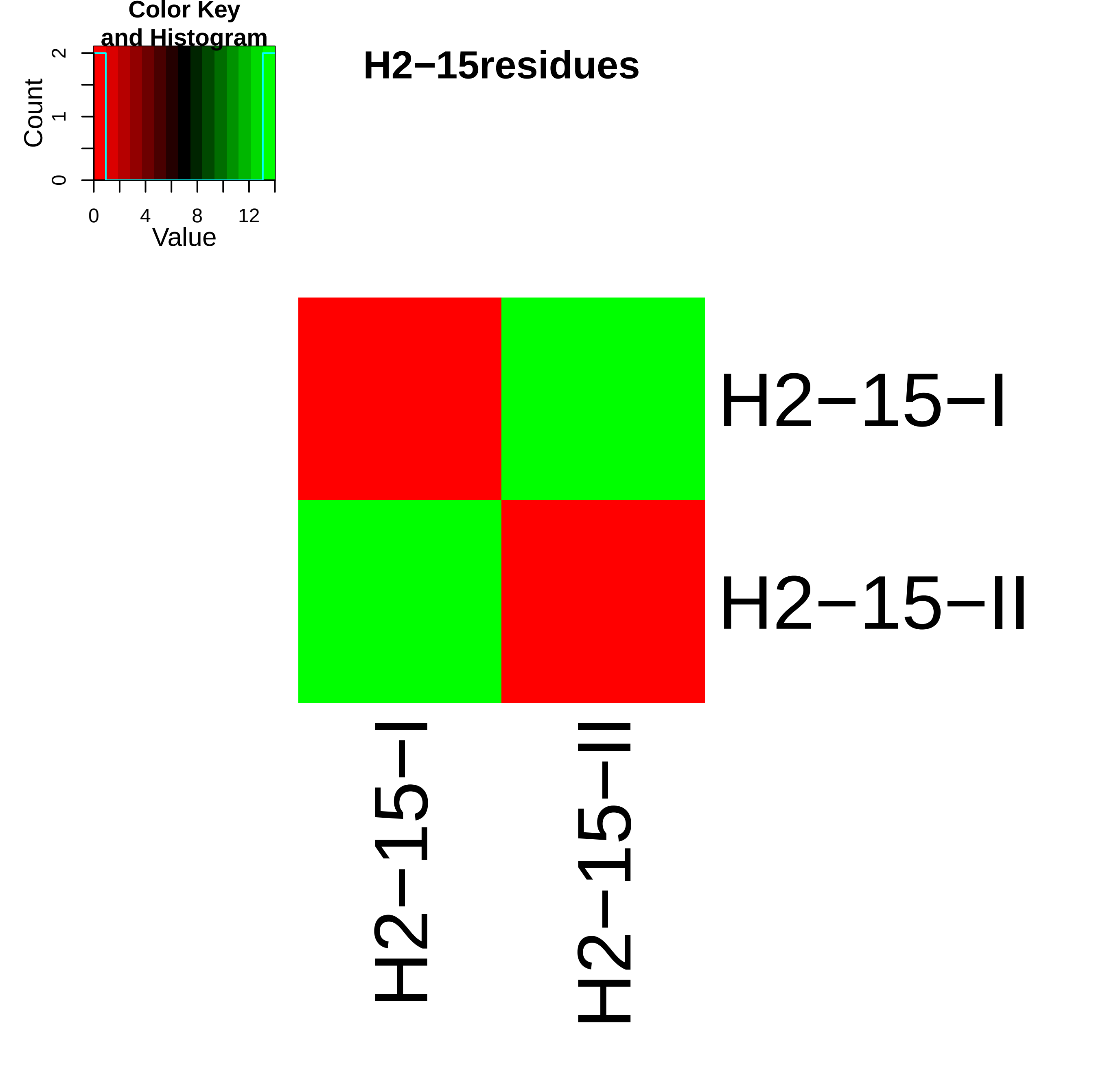
Color Key
and Histogram
Count
Value
0
4
8
12
0
1
2
H2−15residues
H2−15−I
H2−15−II
H2−15−I
H2−15−II

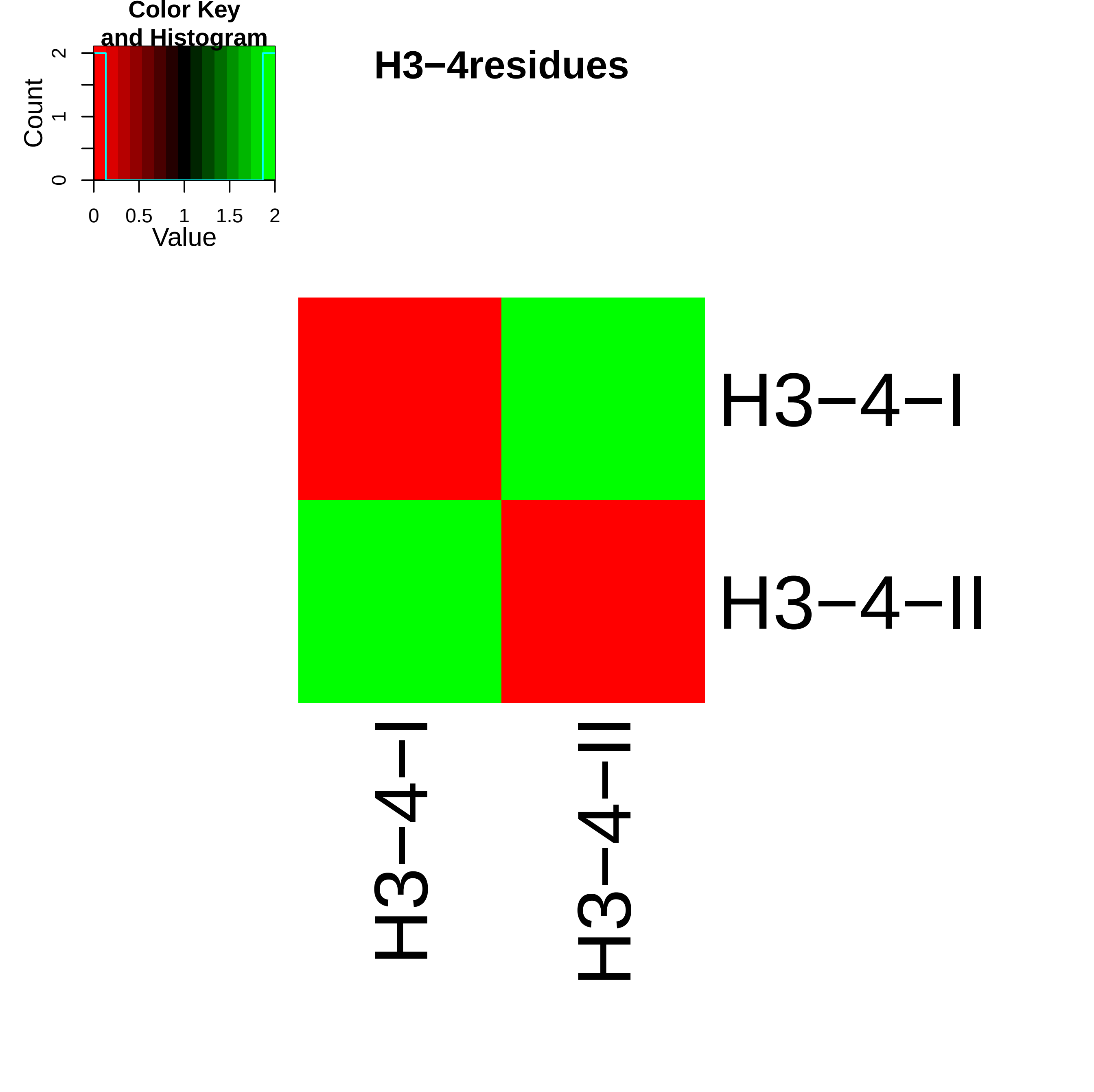
Color Key
and Histogram
Count
Value
0
0.5
1
1.5
2
0
1
2
H3−4residues
H3−4−I
H3−4−II
H3−4−I
H3−4−II

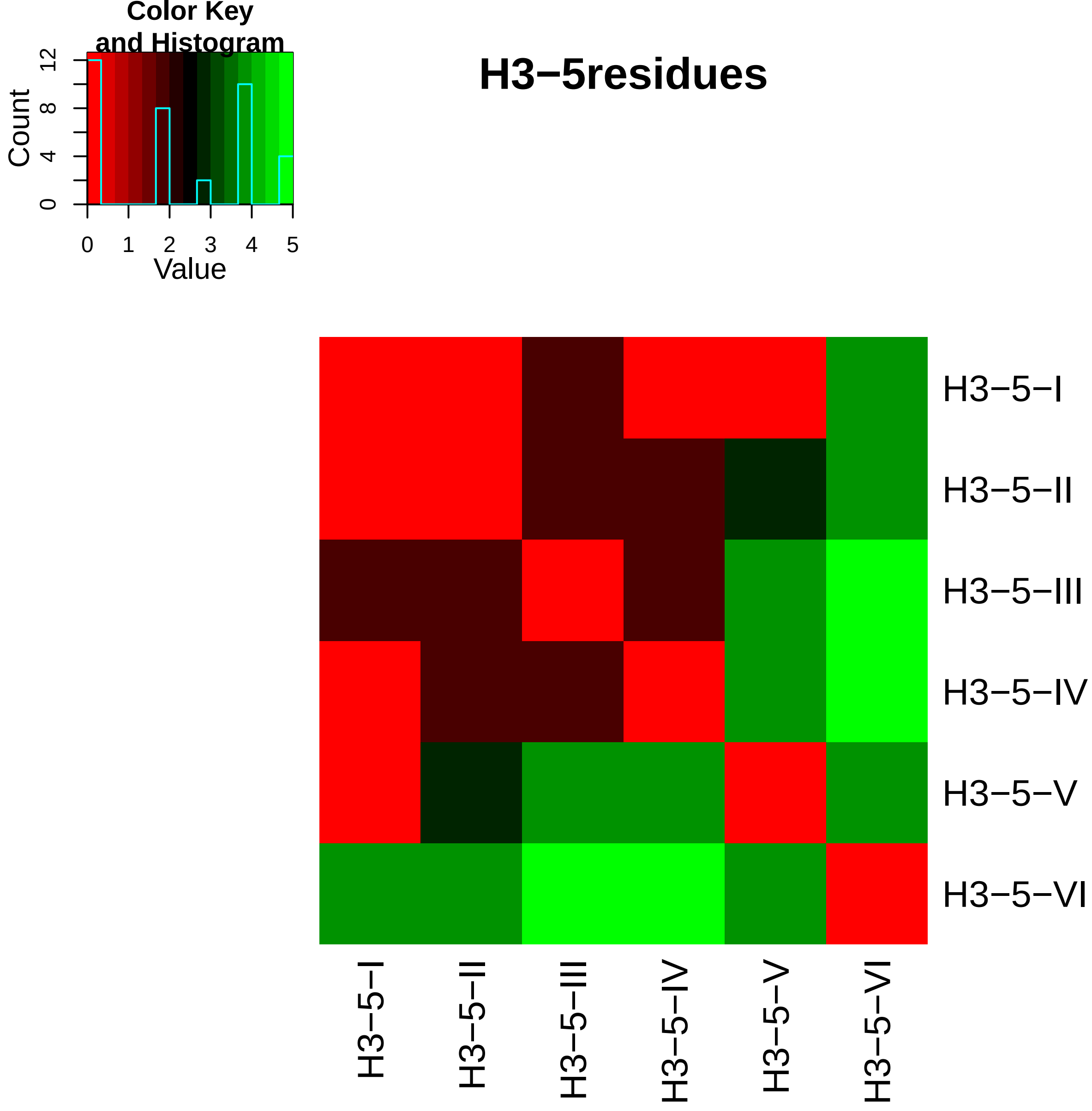
Color Key
and Histogram
Count
Value
0
1
2
3
4
5
0
4
8
12
H3−5residues
H3−5−I
H3−5−II
H3−5−III
H3−5−IV
H3−5−V
H3−5−VI
H3−5−I
H3−5−II
H3−5−III
H3−5−IV
H3−5−V
H3−5−VI

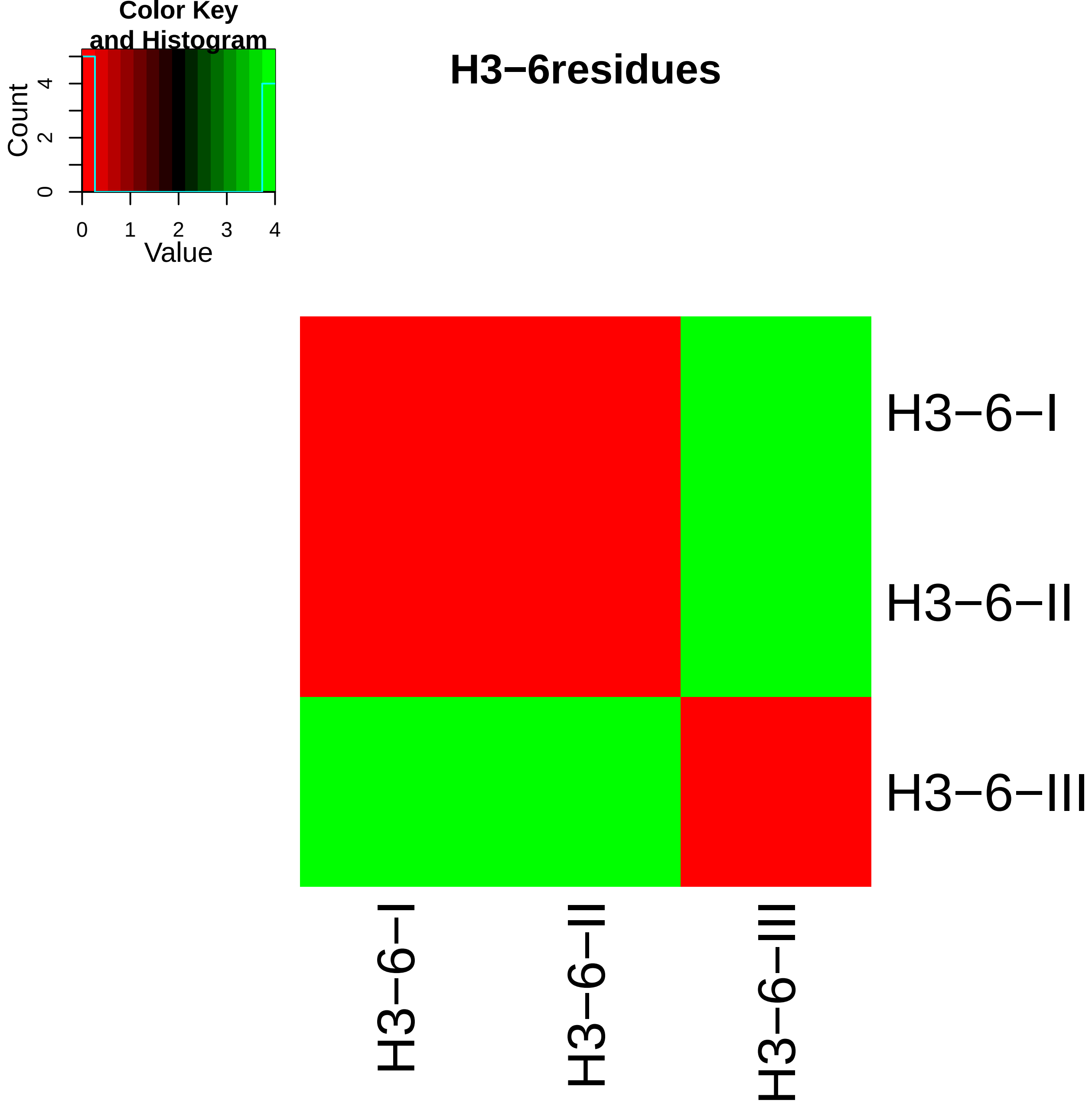
H3−6residues
Color Key
and Histogram
Count
Value
0
1
2
3
4
0
2
4
H3−6−I
H3−6−II
H3−6−III
H3−6−I
H3−6−II
H3−6−III

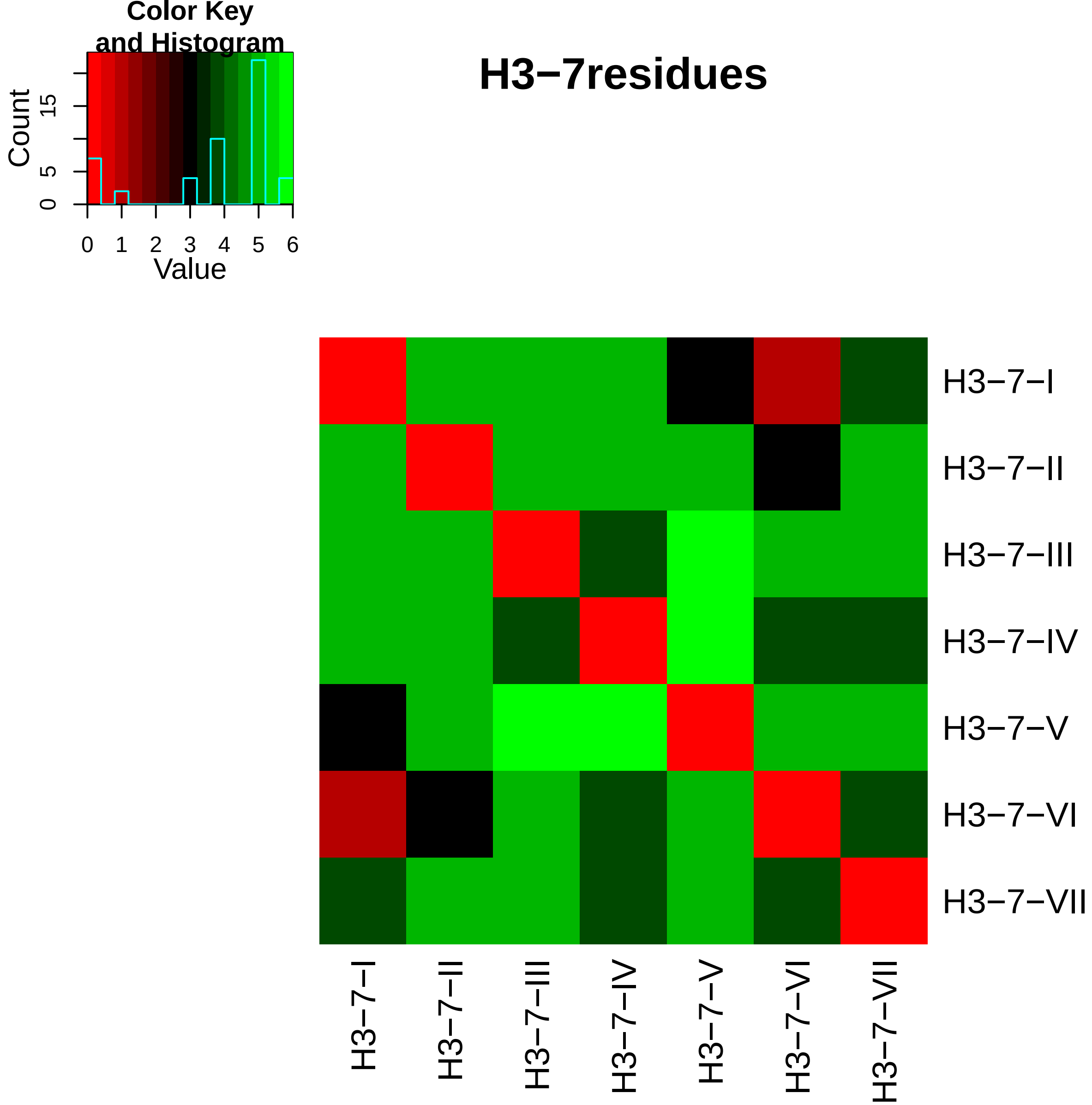
Color Key
and Histogram
Count
0
5
15
0 1 2 3 4 5 6
Value
H3−7residues
H3−7−I
H3−7−II
H3−7−III
H3−7−IV
H3−7−V
H3−7−VI
H3−7−VII
H3−7−I
H3−7−II
H3−7−III
H3−7−IV
H3−7−V
H3−7−VI
H3−7−VII

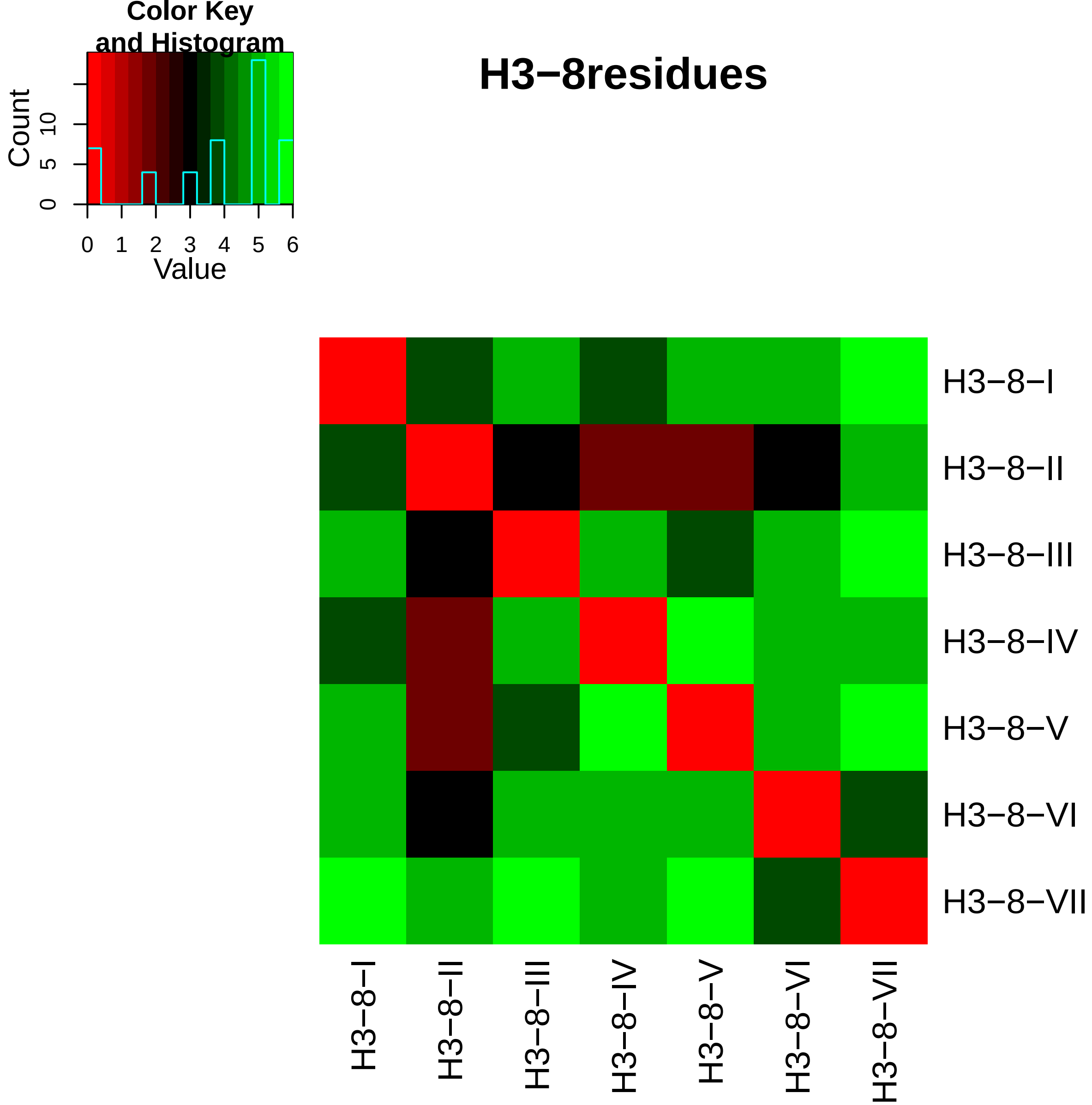
Color Key
and Histogram
Count
0
5
10
0 1 2 3 4 5 6
Value
H3−8residues
H3−8−I
H3−8−II
H3−8−III
H3−8−IV
H3−8−V
H3−8−VI
H3−8−VII
H3−8−I
H3−8−II
H3−8−III
H3−8−IV
H3−8−V
H3−8−VI
H3−8−VII

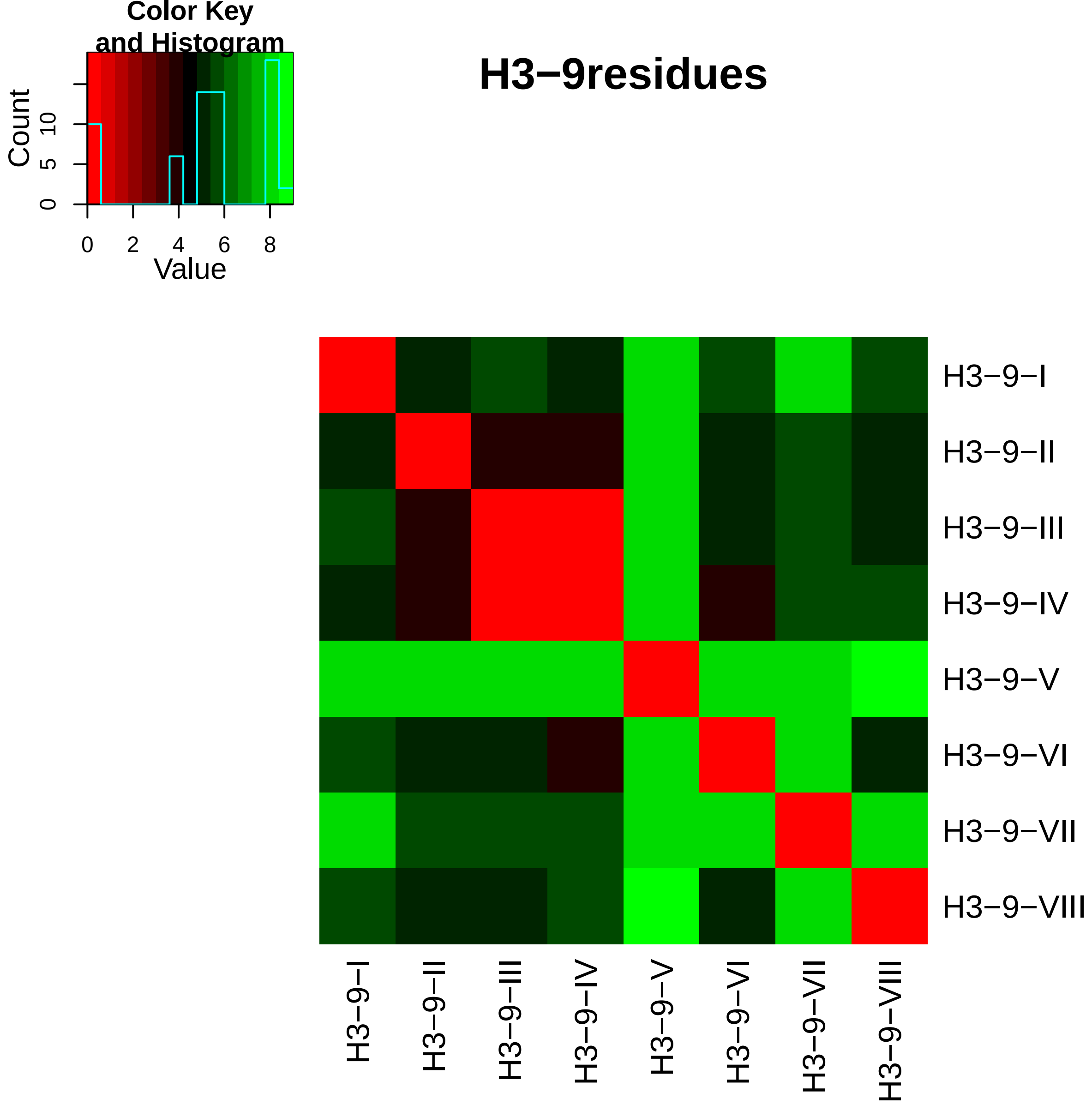
Color Key
and Histogram
Count
0
5
10
0
2
4
6
8
Value
H3−9residues
H3−9−I
H3−9−II
H3−9−III
H3−9−IV
H3−9−V
H3−9−VI
H3−9−VII
H3−9−VIII
H3−9−I
H3−9−II
H3−9−III
H3−9−IV
H3−9−V
H3−9−VI
H3−9−VII
H3−9−VIII

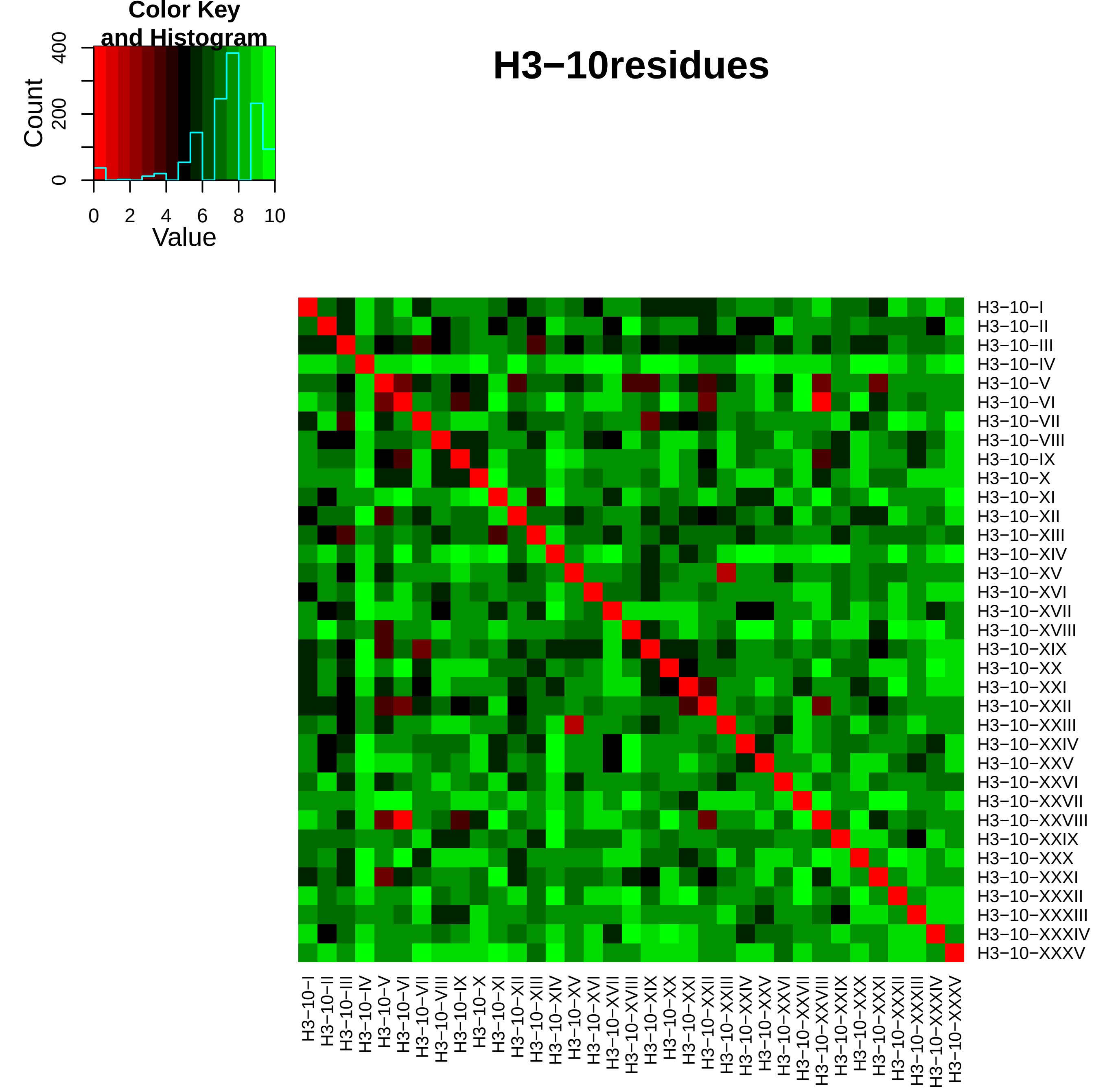

Color Key and Histogram
Count
Value
H3−10residues
H3−10−I
H3−10−II
H3−10−III
H3−10−IV
H3−10−V
H3−10−VI
H3−10−VII
H3−10−VIII
H3−10−IX
H3−10−X
H3−10−XI
H3−10−XII
H3−10−XIII
H3−10−XIV
H3−10−XV
H3−10−XVI
H3−10−XVII
H3−10−XVIII
H3−10−XIX
H3−10−XX
H3−10−XXI
H3−10−XXII
H3−10−XXIII
H3−10−XXIV
H3−10−XXV
H3−10−XXVI
H3−10−XXVII
H3−10−XXVIII
H3−10−XXIX
H3−10−XXX
H3−10−XXXI
H3−10−XXXII
H3−10−XXXIII
H3−10−XXXIV
H3−10−XXXV

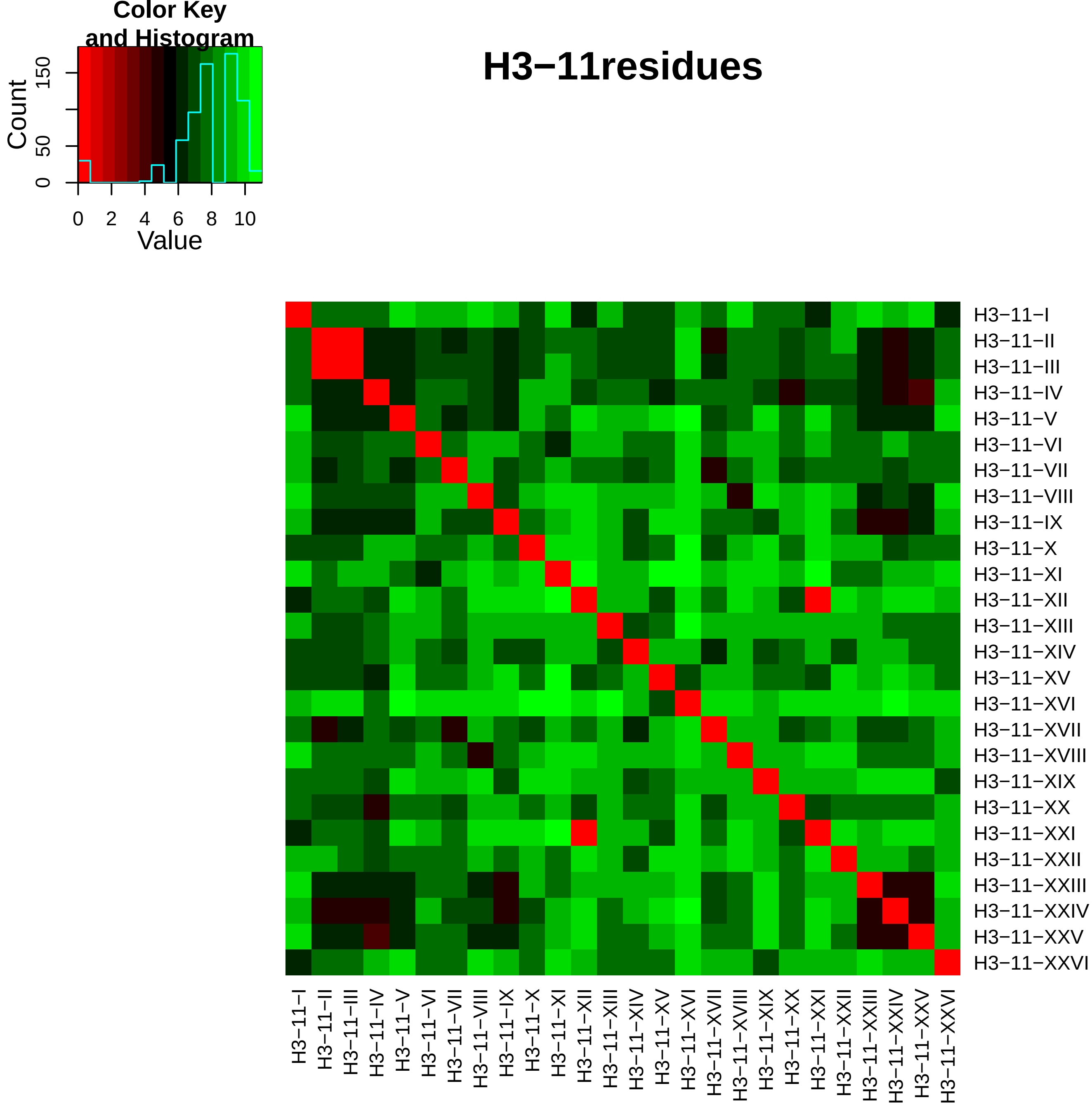

Color Key and Histogram
Count
0
50
150
0
2
4
6
8
10
Value
H3−11residues
H3−11−I
H3−11−II
H3−11−III
H3−11−IV
H3−11−V
H3−11−VI
H3−11−VII
H3−11−VIII
H3−11−IX
H3−11−X
H3−11−XI
H3−11−XII
H3−11−XIII
H3−11−XIV
H3−11−XV
H3−11−XVI
H3−11−XVII
H3−11−XVIII
H3−11−XIX
H3−11−XX
H3−11−XXI
H3−11−XXII
H3−11−XXIII
H3−11−XXIV
H3−11−XXV
H3−11−XXVI

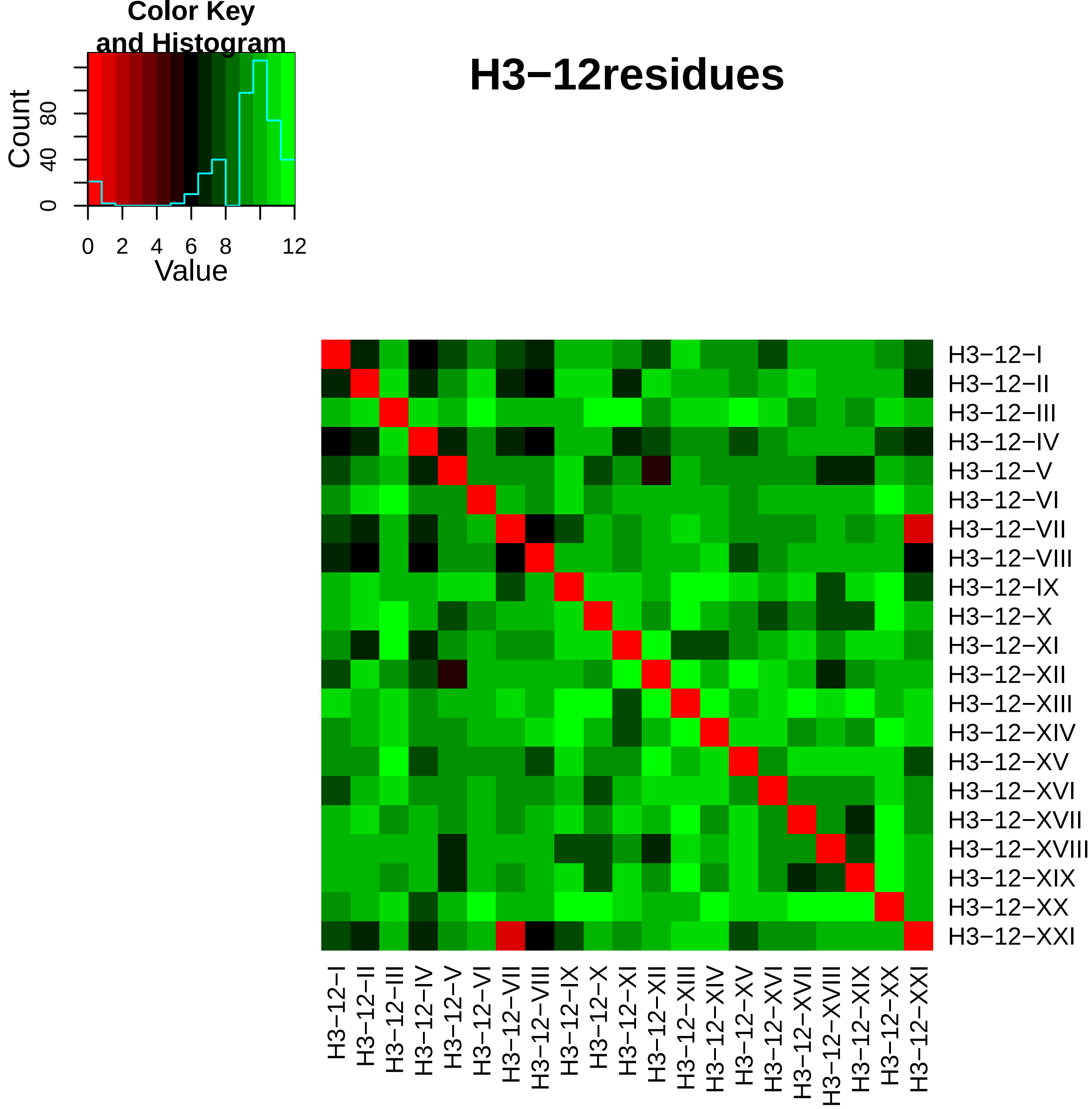
Color Key
and Histogram
Count
0
40
80
0
2
4
6
8
12
Value
H3−12residues
H3−12−I
H3−12−II
H3−12−III
H3−12−IV
H3−12−V
H3−12−VI
H3−12−VII
H3−12−VIII
H3−12−IX
H3−12−X
H3−12−XI
H3−12−XII
H3−12−XIII
H3−12−XIV
H3−12−XV
H3−12−XVI
H3−12−XVII
H3−12−XVIII
H3−12−XIX
H3−12−XX
H3−12−XXI

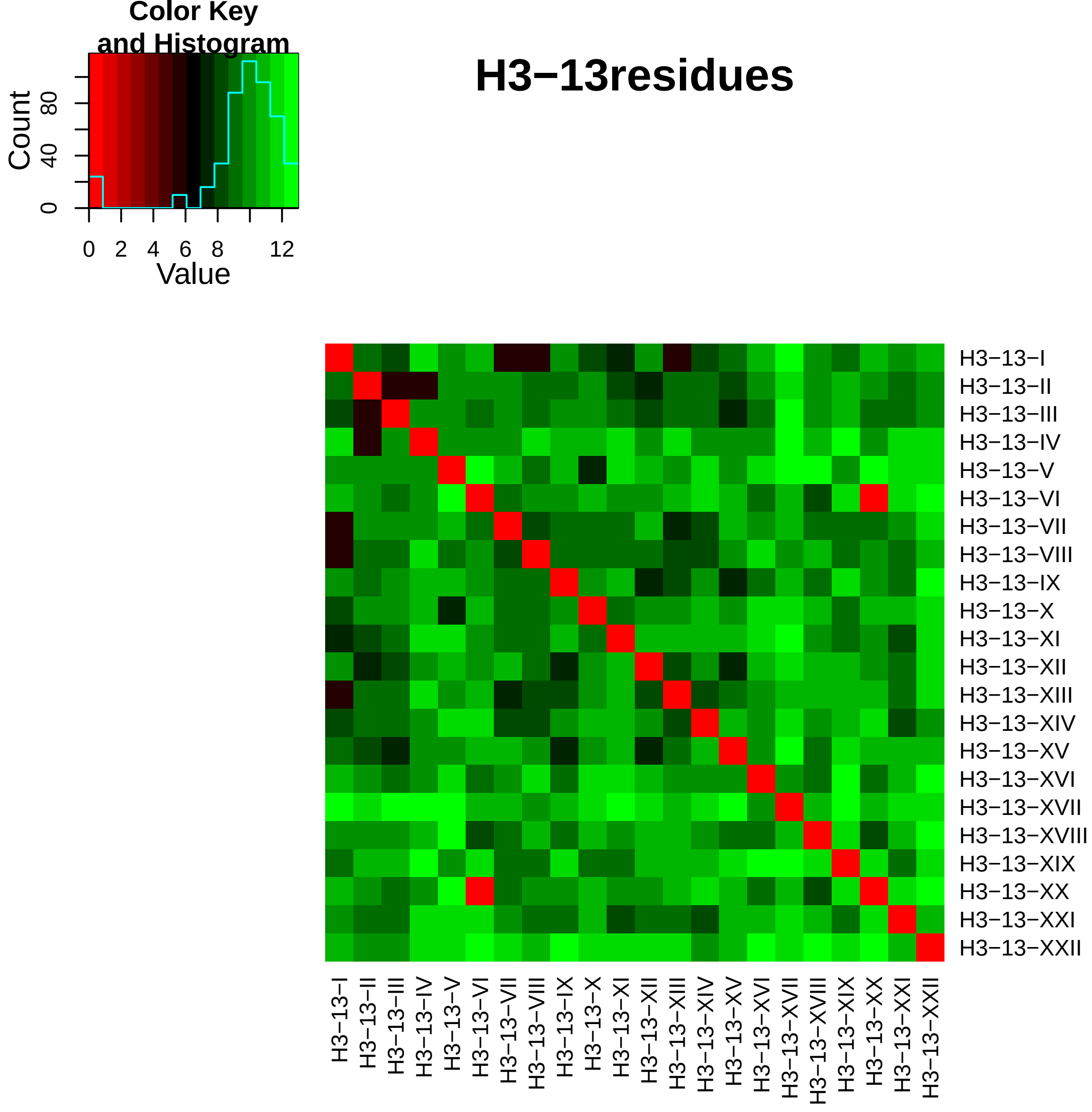
Color Key
and Histogram
Count
0
40
80
0 2 4 6 8 12
Value
H3−13residues
H3−13−I
H3−13−II
H3−13−III
H3−13−IV
H3−13−V
H3−13−VI
H3−13−VII
H3−13−VIII
H3−13−IX
H3−13−X
H3−13−XI
H3−13−XII
H3−13−XIII
H3−13−XIV
H3−13−XV
H3−13−XVI
H3−13−XVII
H3−13−XVIII
H3−13−XIX
H3−13−XX
H3−13−XXI
H3−13−XXII

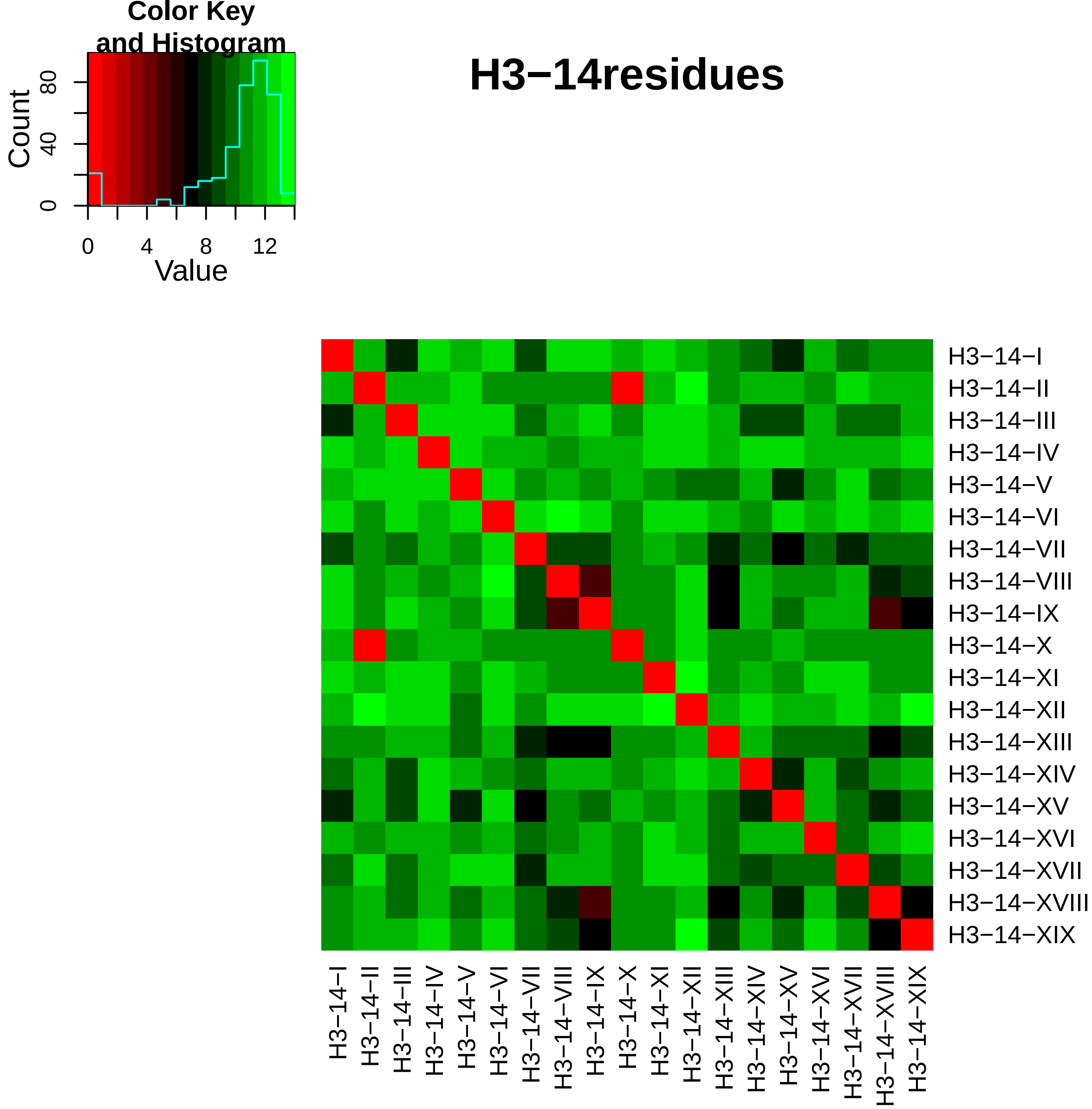
Color Key and Histogram
Count
Value
0
4
8
12
0
40
80
H3−14residues
H3−14−I
H3−14−II
H3−14−III
H3−14−IV
H3−14−V
H3−14−VI
H3−14−VII
H3−14−VIII
H3−14−IX
H3−14−X
H3−14−XI
H3−14−XII
H3−14−XIII
H3−14−XIV
H3−14−XV
H3−14−XVI
H3−14−XVII
H3−14−XVIII
H3−14−XIX

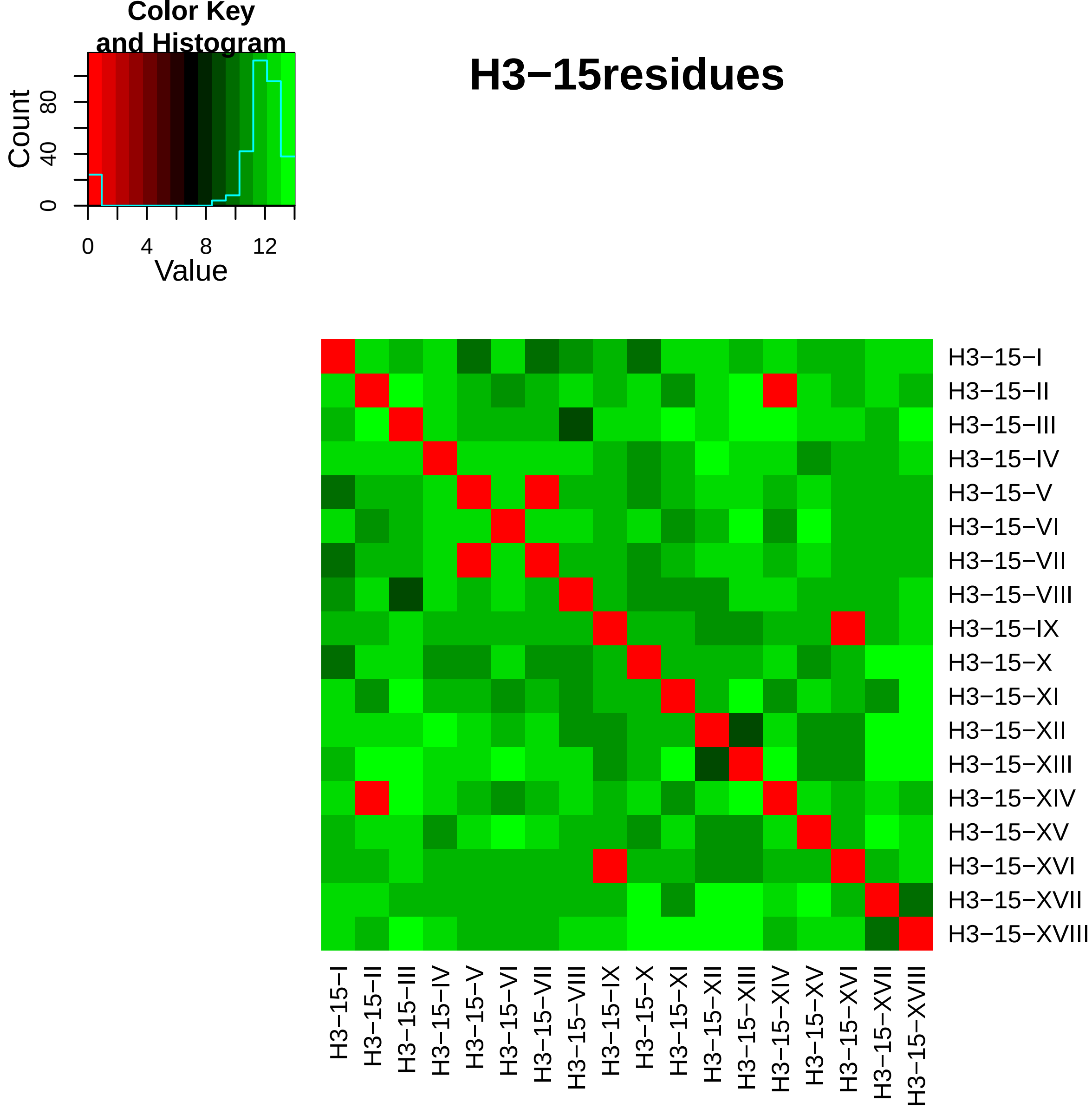

Color Key
and Histogram
Count
0
40
80
0
4
8
12
Value
H3−15residues
H3−15−I
H3−15−II
H3−15−III
H3−15−IV
H3−15−V
H3−15−VI
H3−15−VII
H3−15−VIII
H3−15−IX
H3−15−X
H3−15−XI
H3−15−XII
H3−15−XIII
H3−15−XIV
H3−15−XV
H3−15−XVI
H3−15−XVII
H3−15−XVIII

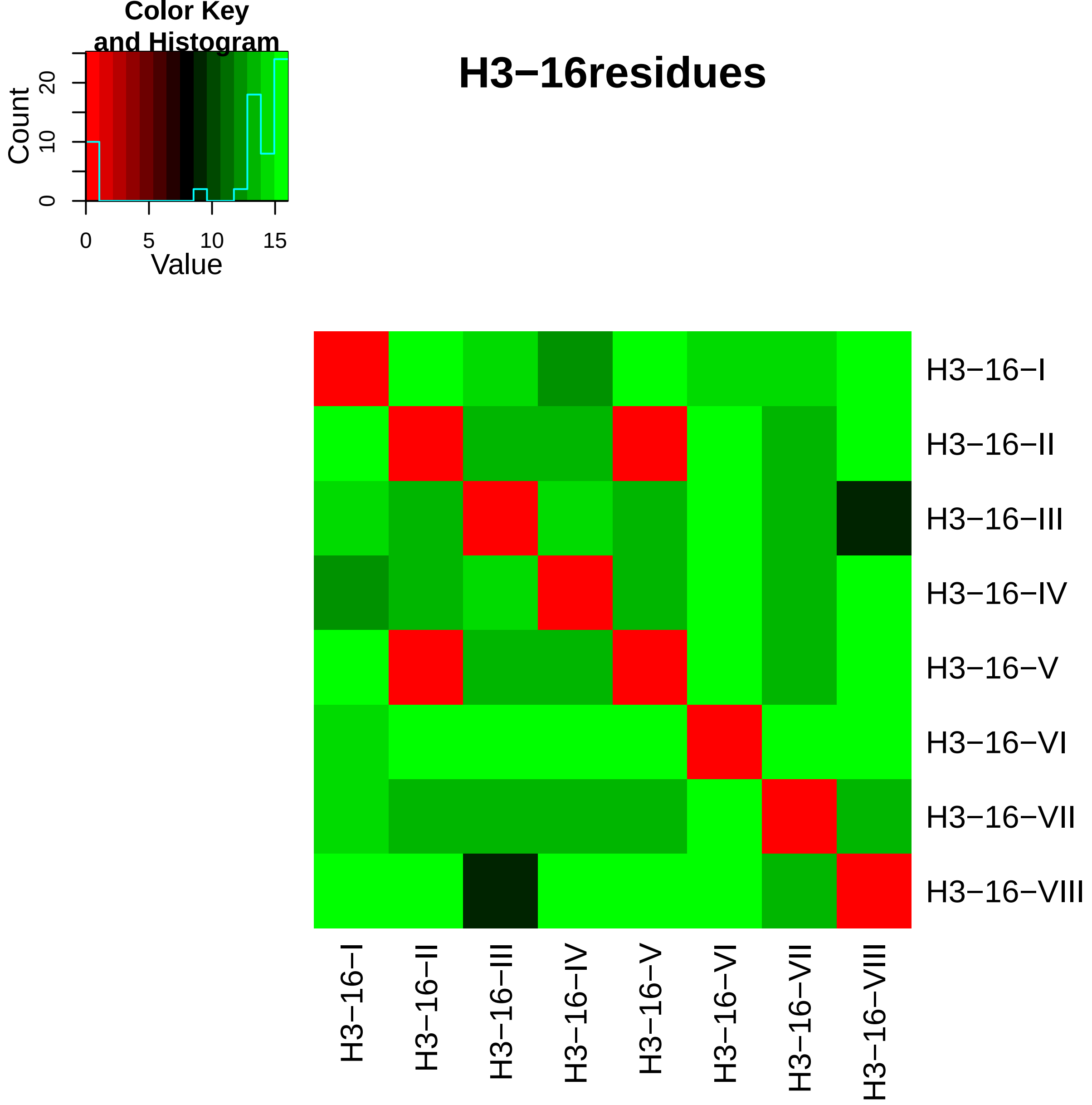

Color Key
and Histogram
Count
Value
0
10
20
0
5
10
15
H3−16residues
H3−16−I
H3−16−II
H3−16−III
H3−16−IV
H3−16−V
H3−16−VI
H3−16−VII
H3−16−VIII

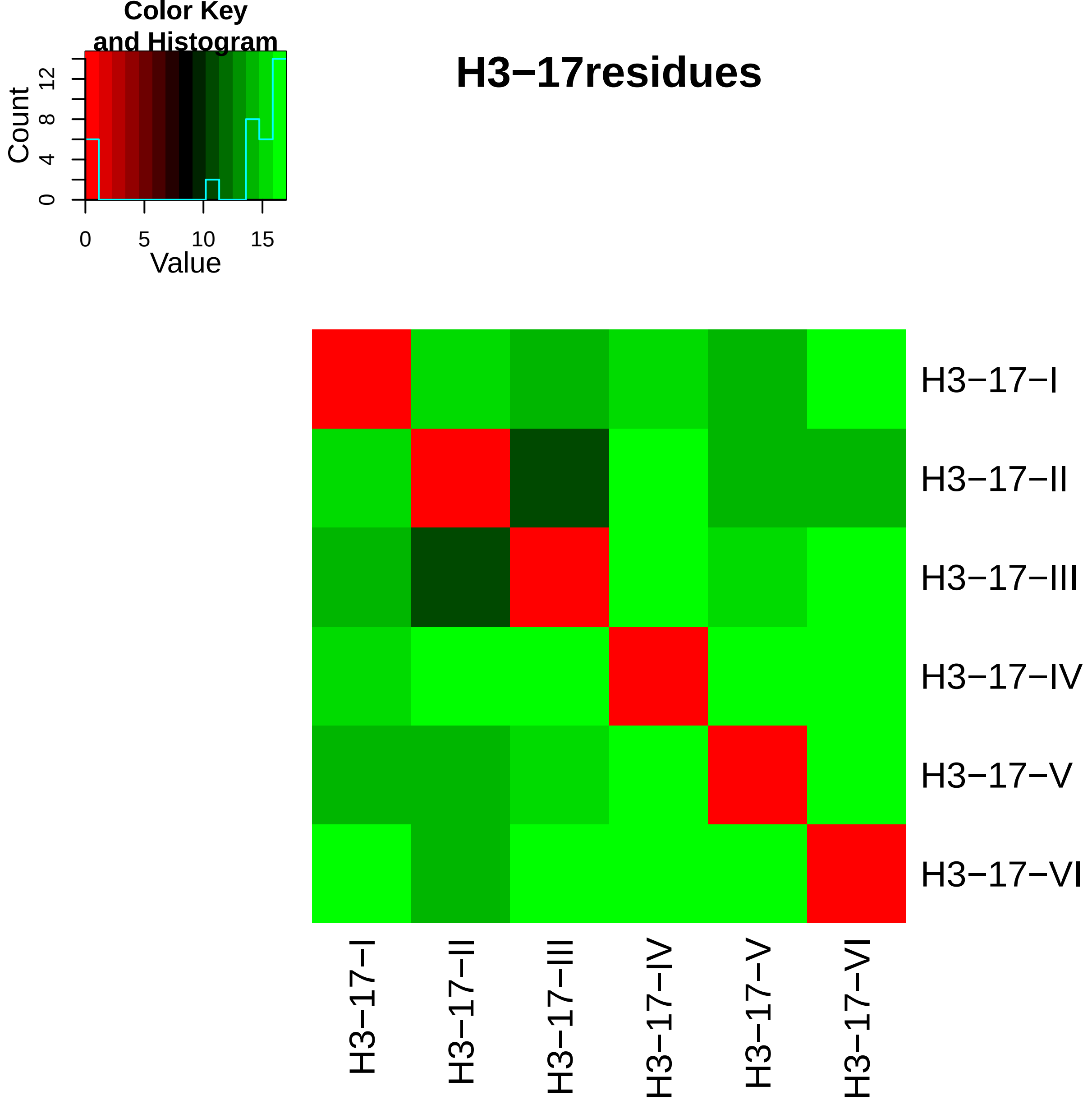

Color Key
and Histogram
Count
0
4
8
12
0
5
10
15
Value
H3−17residues
H3−17−I
H3−17−II
H3−17−III
H3−17−IV
H3−17−V
H3−17−VI
H3−17−I
H3−17−II
H3−17−III
H3−17−IV
H3−17−V
H3−17−VI

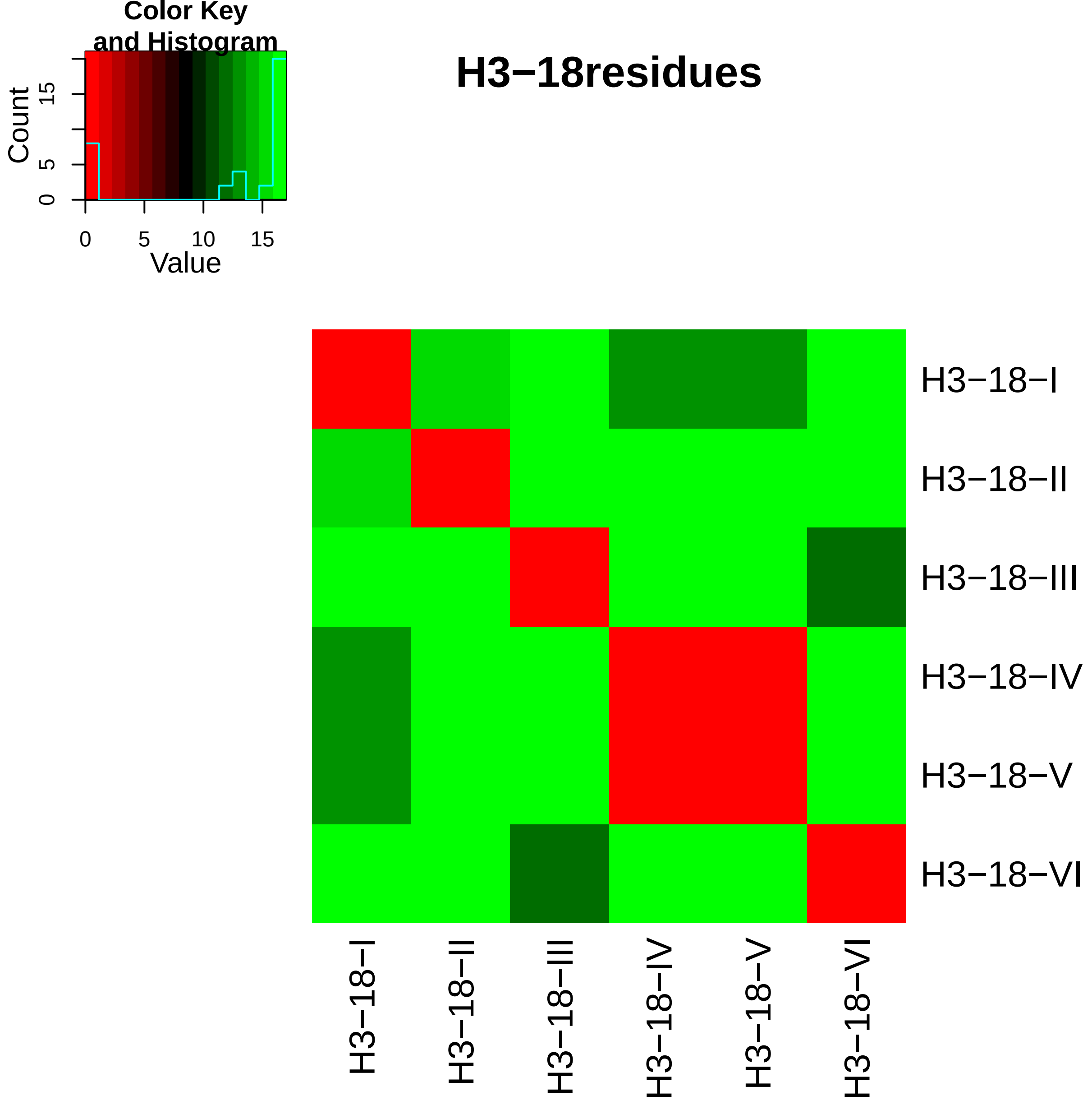
Color Key
and Histogram
Count
0
5
15
0
5
10
15
Value
H3−18residues
H3−18−I
H3−18−II
H3−18−III
H3−18−IV
H3−18−V
H3−18−VI
H3−18−I
H3−18−II
H3−18−III
H3−18−IV
H3−18−V
H3−18−VI

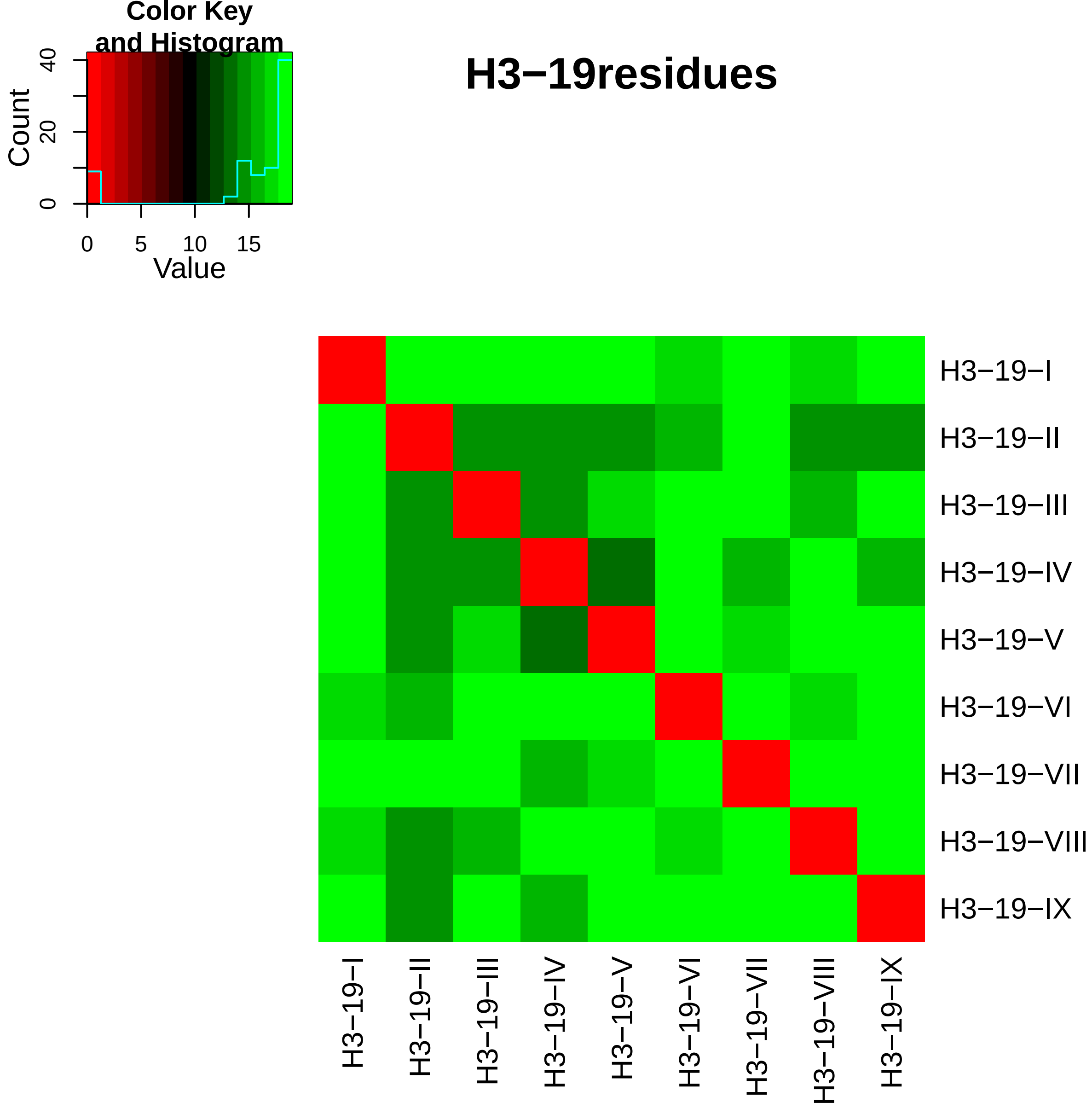

Color Key
and Histogram
Count
Value
0
5
10
15
0
20
40
H3−19residues
H3−19−I
H3−19−II
H3−19−III
H3−19−IV
H3−19−V
H3−19−VI
H3−19−VII
H3−19−VIII
H3−19−IX

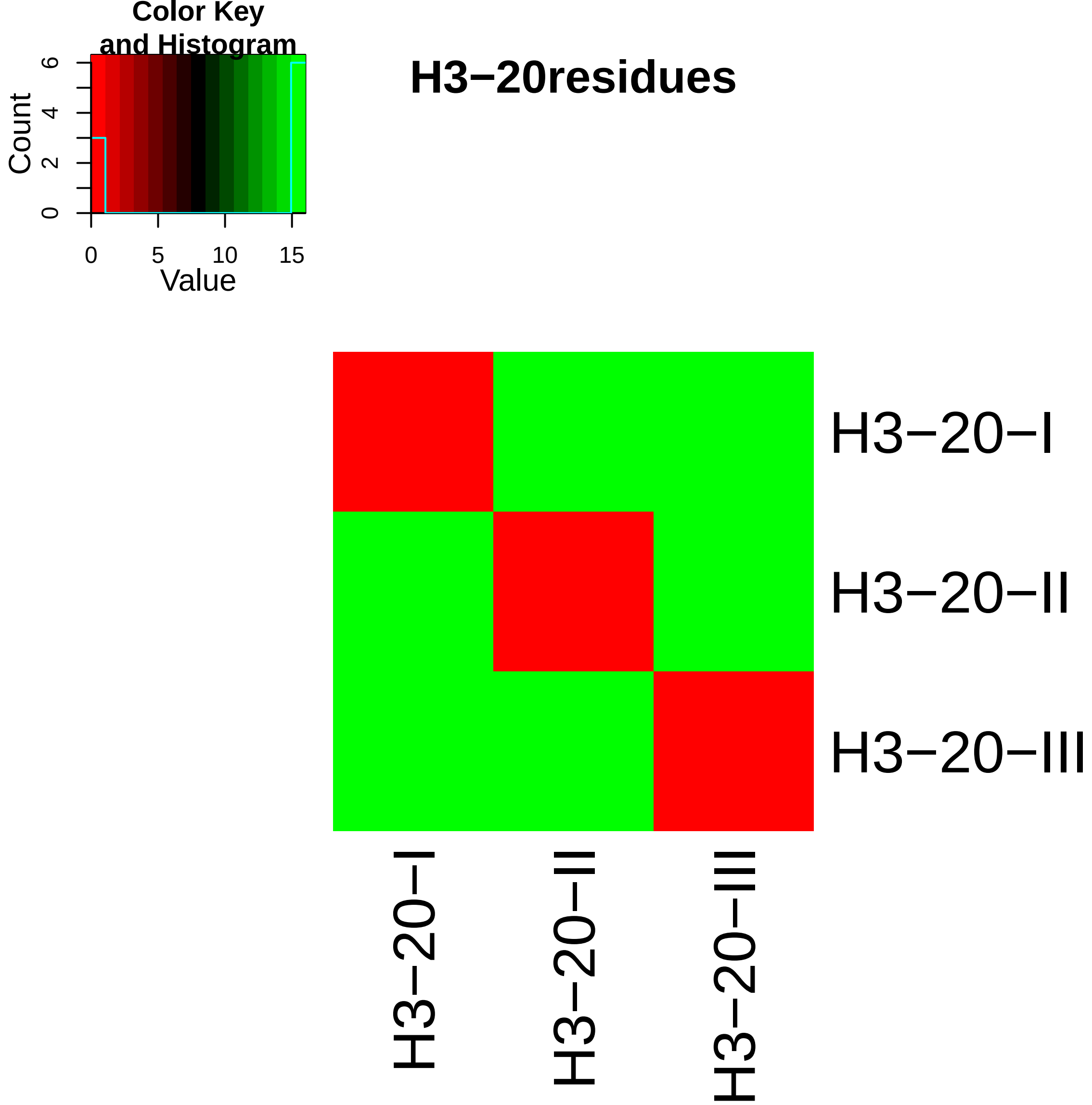
Color Key
and Histogram
Count
Value
0
5
10
15
0
2
4
6
H3−20residues
H3−20−I
H3−20−II
H3−20−III
H3−20−I
H3−20−II
H3−20−III

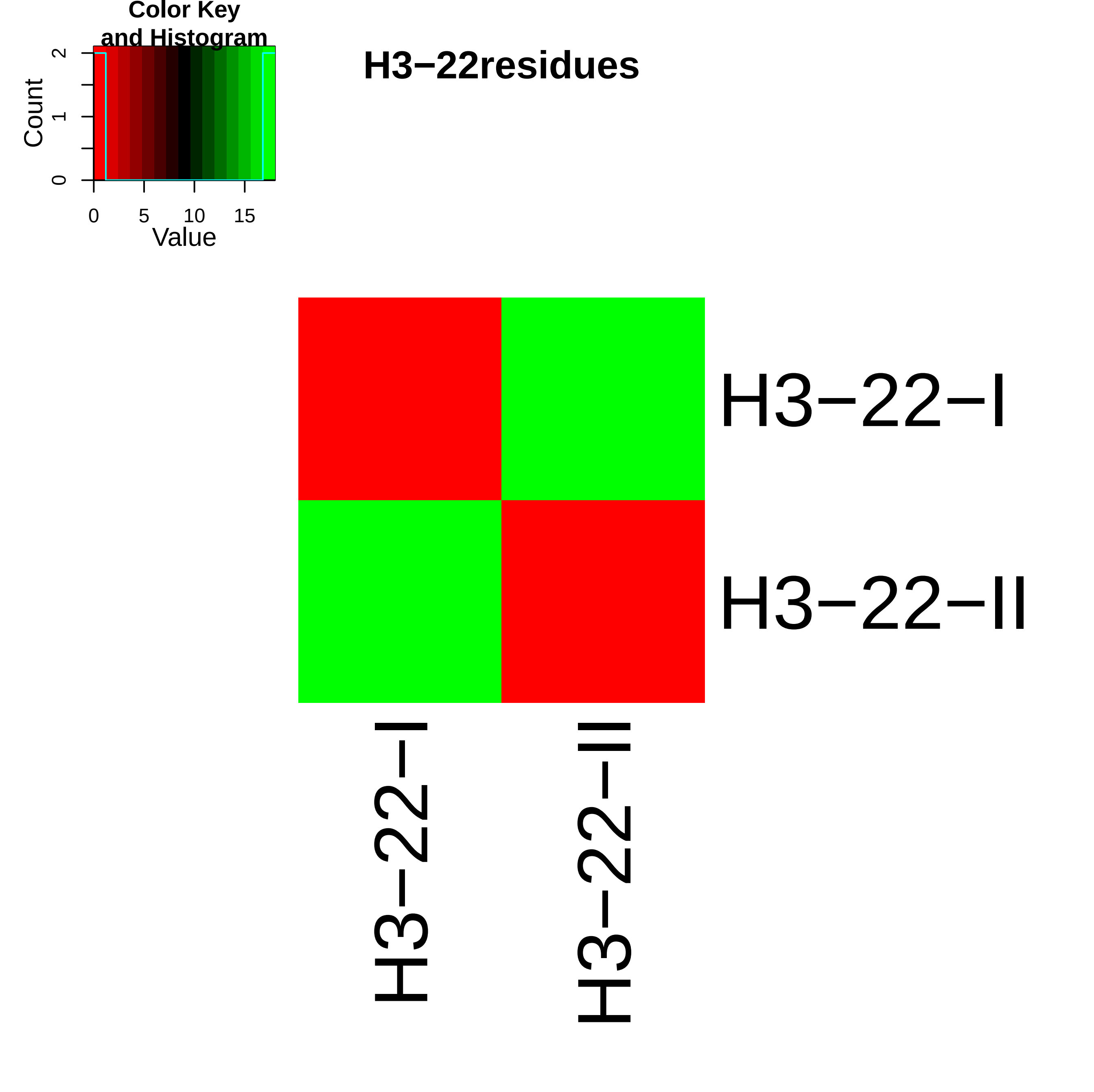

Color Key
and Histogram
Count
Value
0
5
10
15
0
1
2
H3−22residues
H3−22−I
H3−22−II
H3−22−I
H3−22−II

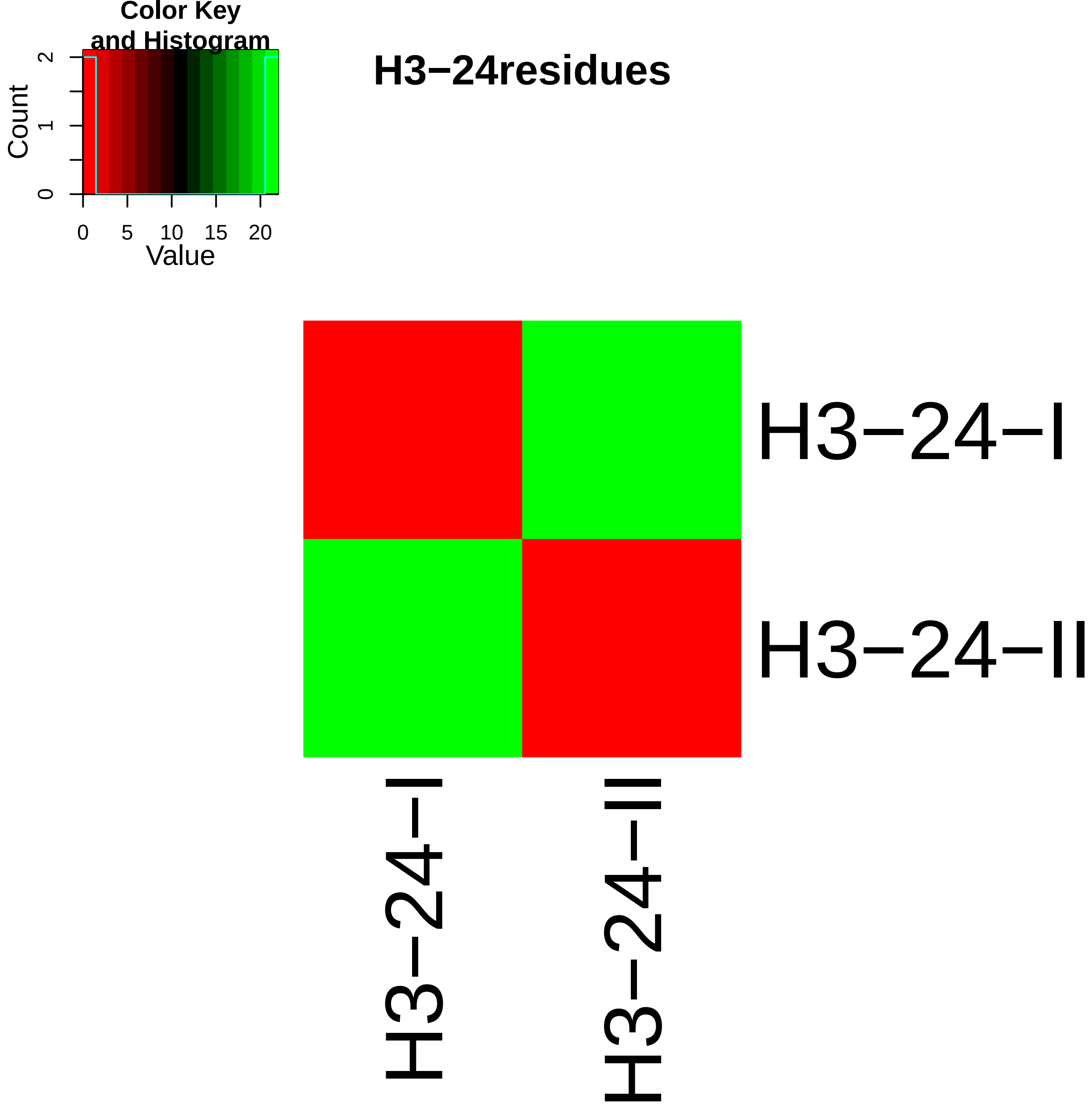

Color Key
and Histogram
Count
0
1
2
0
5
10
15
20
Value
H3−24residues
H3−24−I
H3−24−II
H3−24−I
H3−24−II

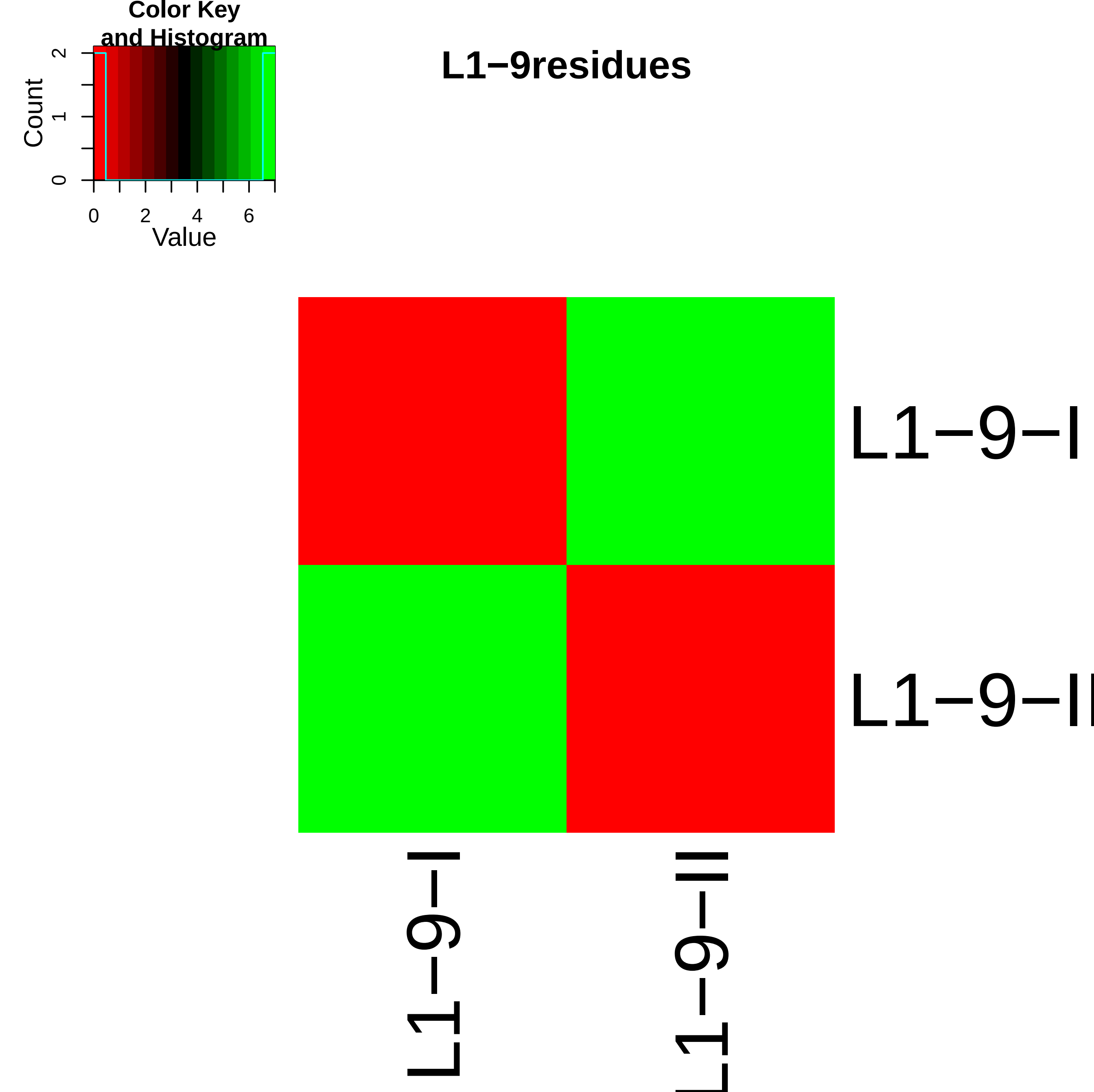
Color Key
and Histogram
Count
0
1
2
0
2
4
6
Value
L1−9residues
L1−9−I
L1−9−II
L1−9−I
L1−9−II

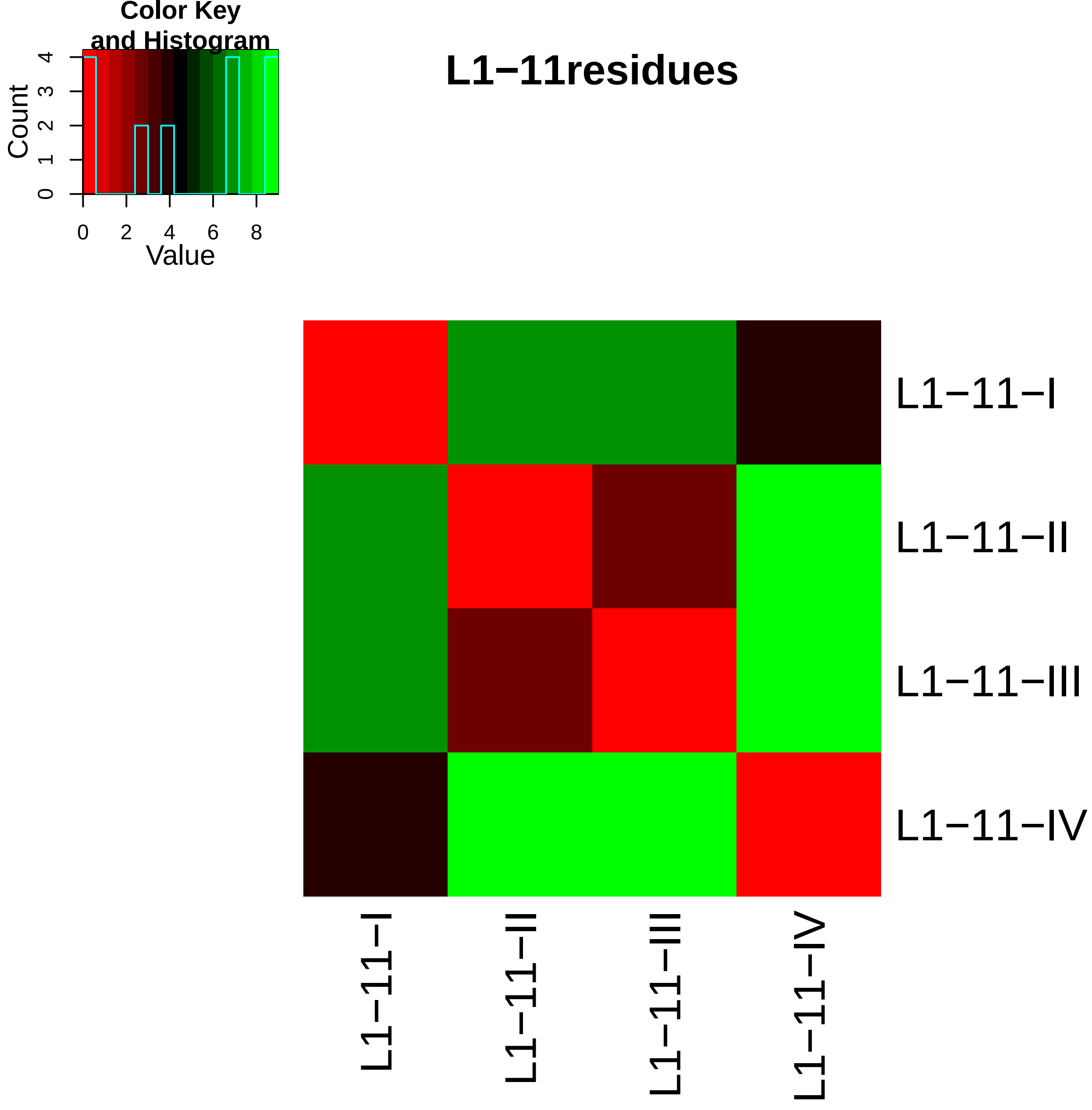
Color Key
and Histogram
Count
Value
0
1
2
3
4
0
2
4
6
8
L1−11residues
L1−11−I
L1−11−II
L1−11−III
L1−11−IV
L1−11−I
L1−11−II
L1−11−III
L1−11−IV

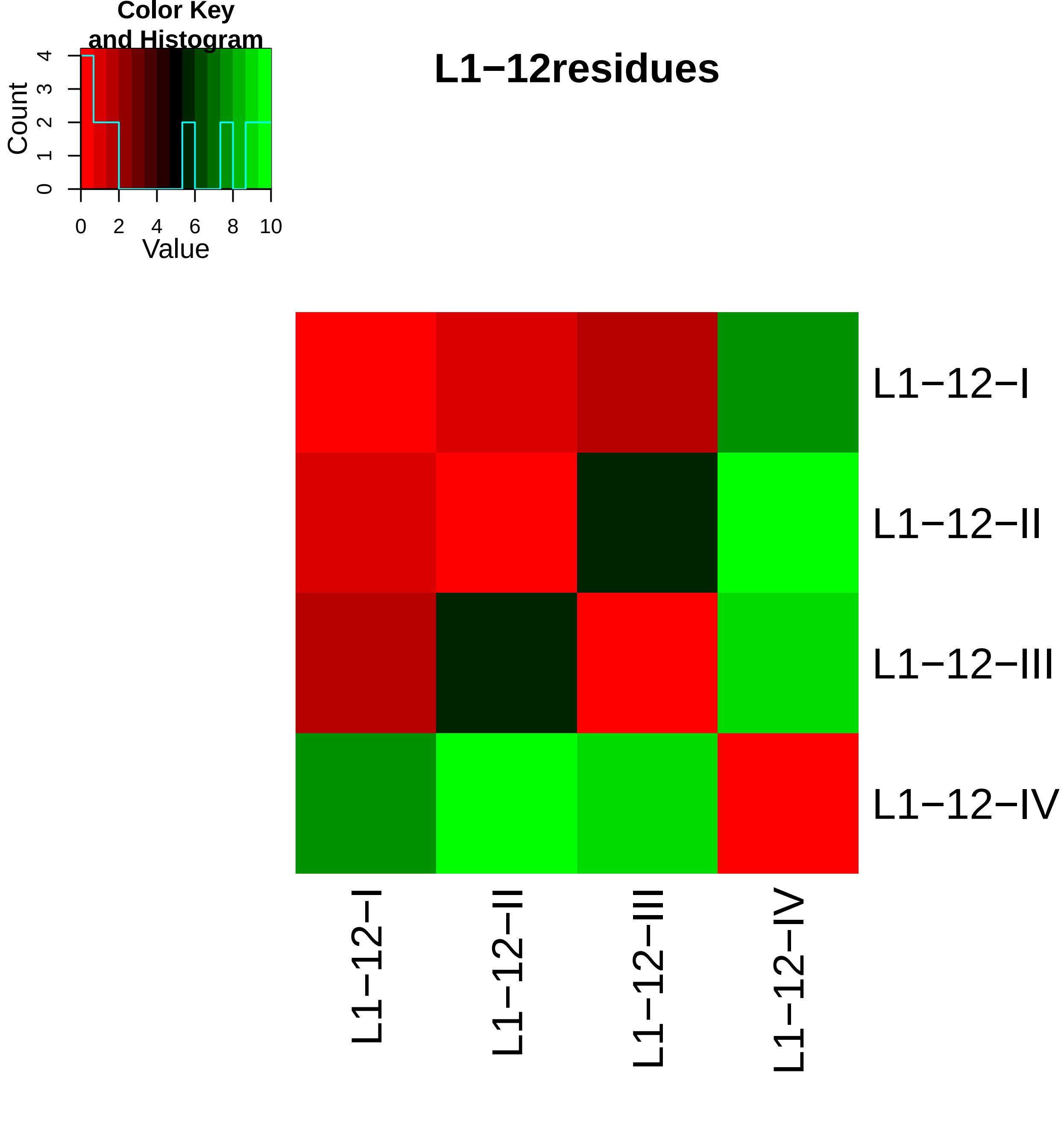

Color Key
and Histogram
Count
Value
0
1
2
3
4
0
2
4
6
8
10
L1−12residues
L1−12−I
L1−12−II
L1−12−III
L1−12−IV
L1−12−I
L1−12−II
L1−12−III
L1−12−IV

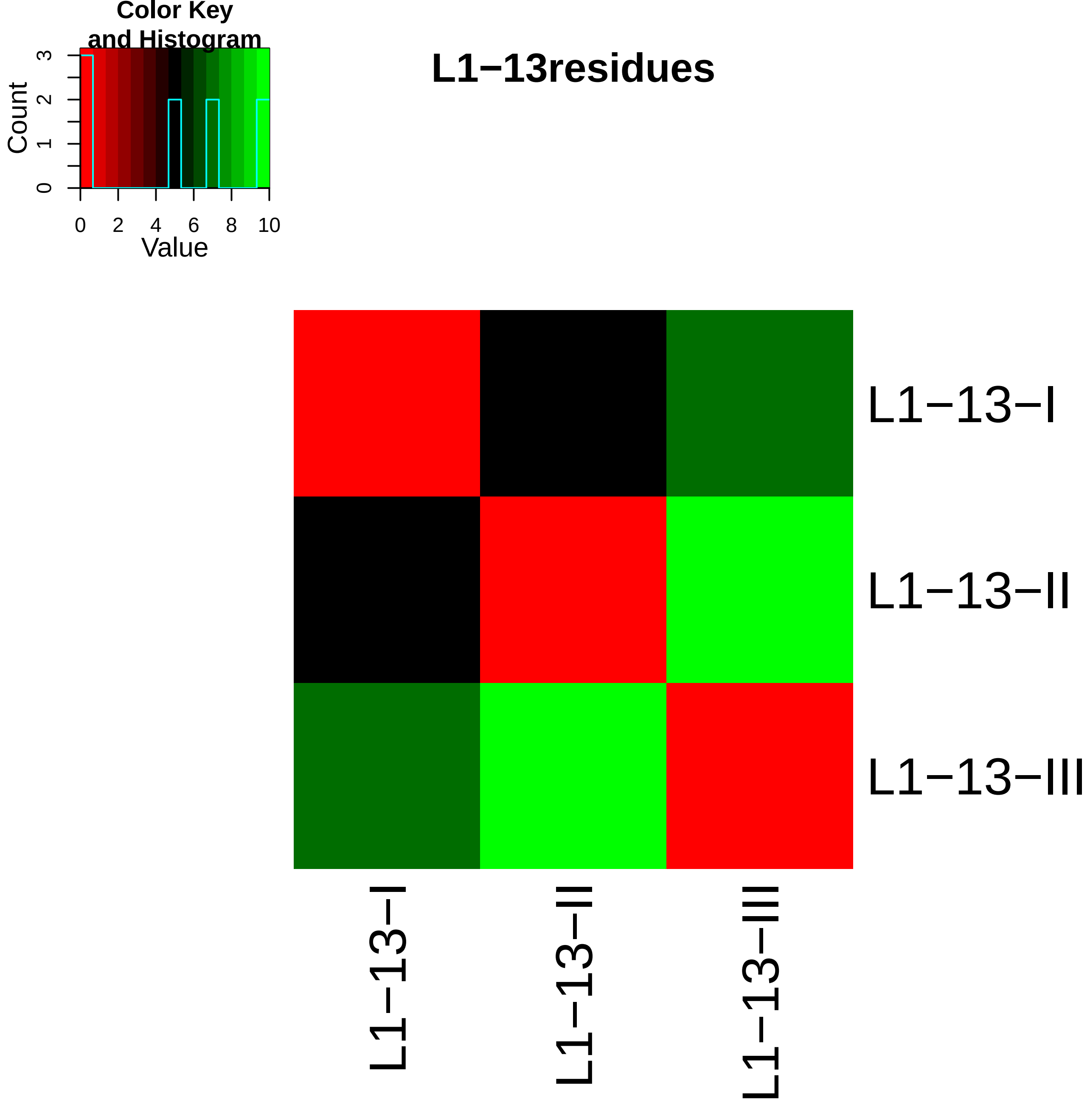

Color Key
and Histogram
Count
0
1
2
3
Value
0
2
4
6
8
10
L1−13residues
L1−13−I
L1−13−II
L1−13−III
L1−13−I
L1−13−II
L1−13−III

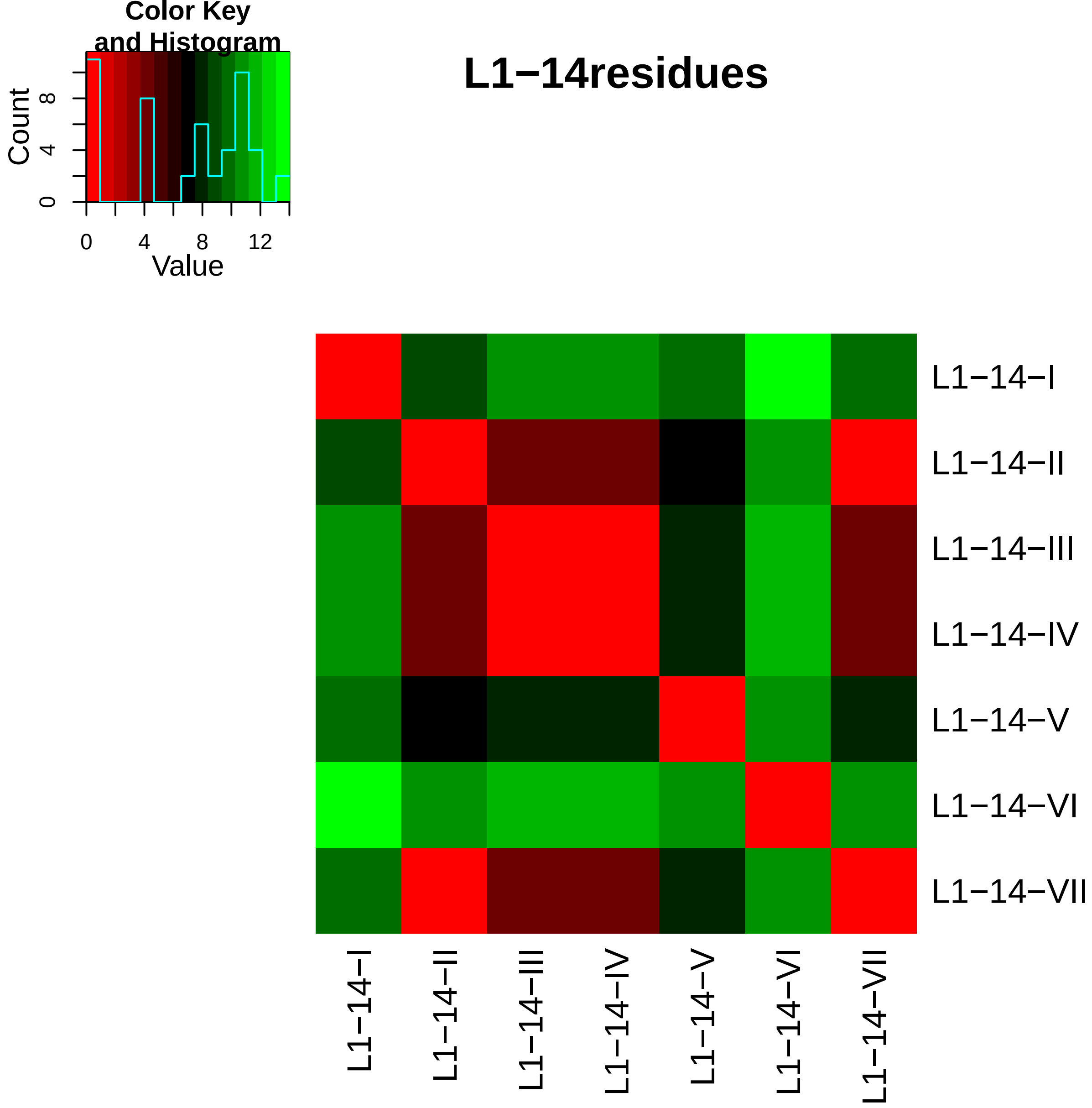
Color Key
and Histogram
Count
0
4
8
0
4
8
12
Value
L1−14residues
L1−14−I
L1−14−II
L1−14−III
L1−14−IV
L1−14−V
L1−14−VI
L1−14−VII

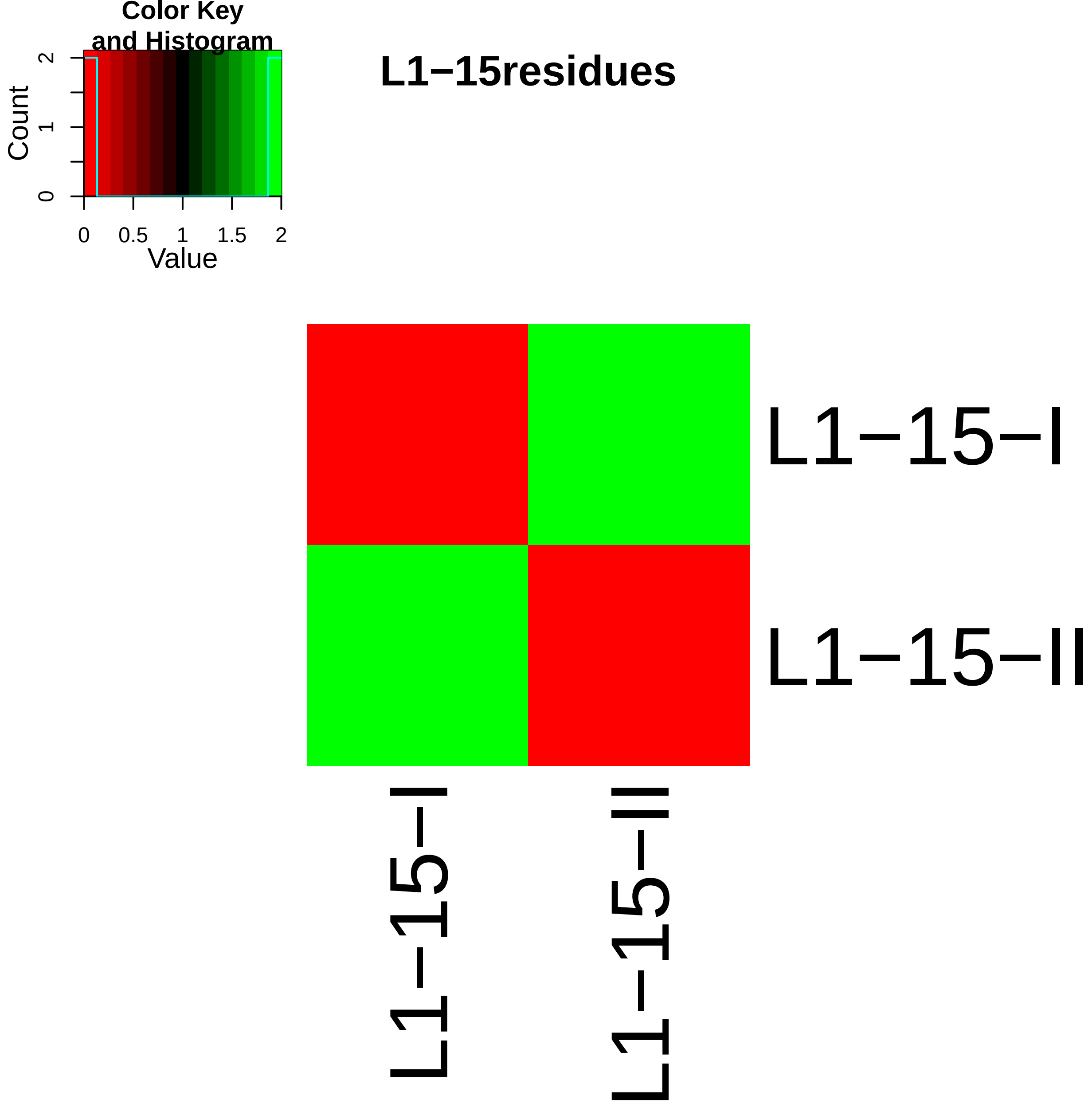

Color Key
and Histogram
Count
Value
0
1
2
0
0.5
1
1.5
2
L1−15residues
L1−15−I
L1−15−II
L1−15−I
L1−15−II

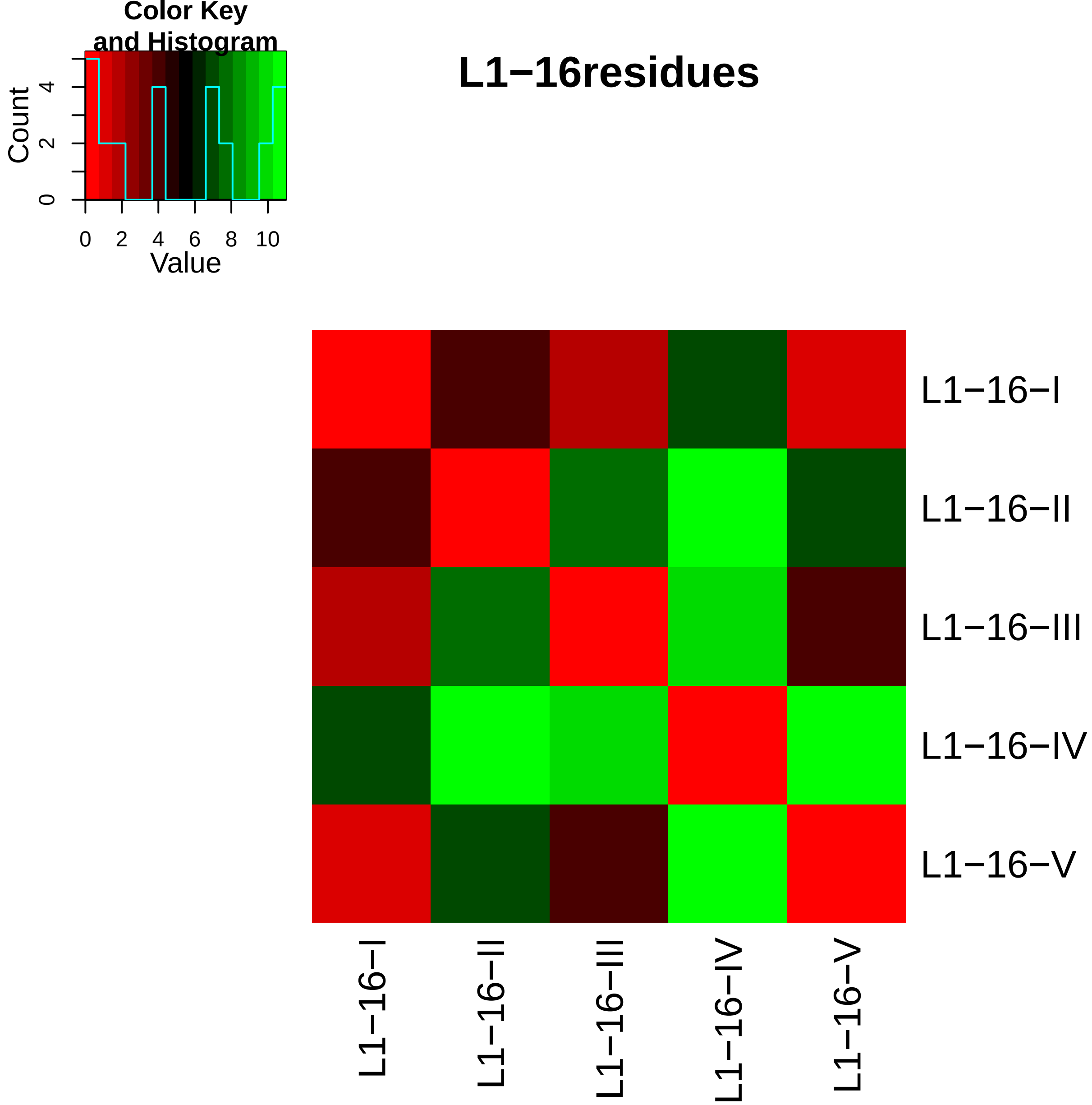

Color Key
and Histogram
Count
0
2
4
0
2
4
6
8
10
Value
L1−16residues
L1−16−I
L1−16−II
L1−16−III
L1−16−IV
L1−16−V
L1−16−I
L1−16−II
L1−16−III
L1−16−IV
L1−16−V

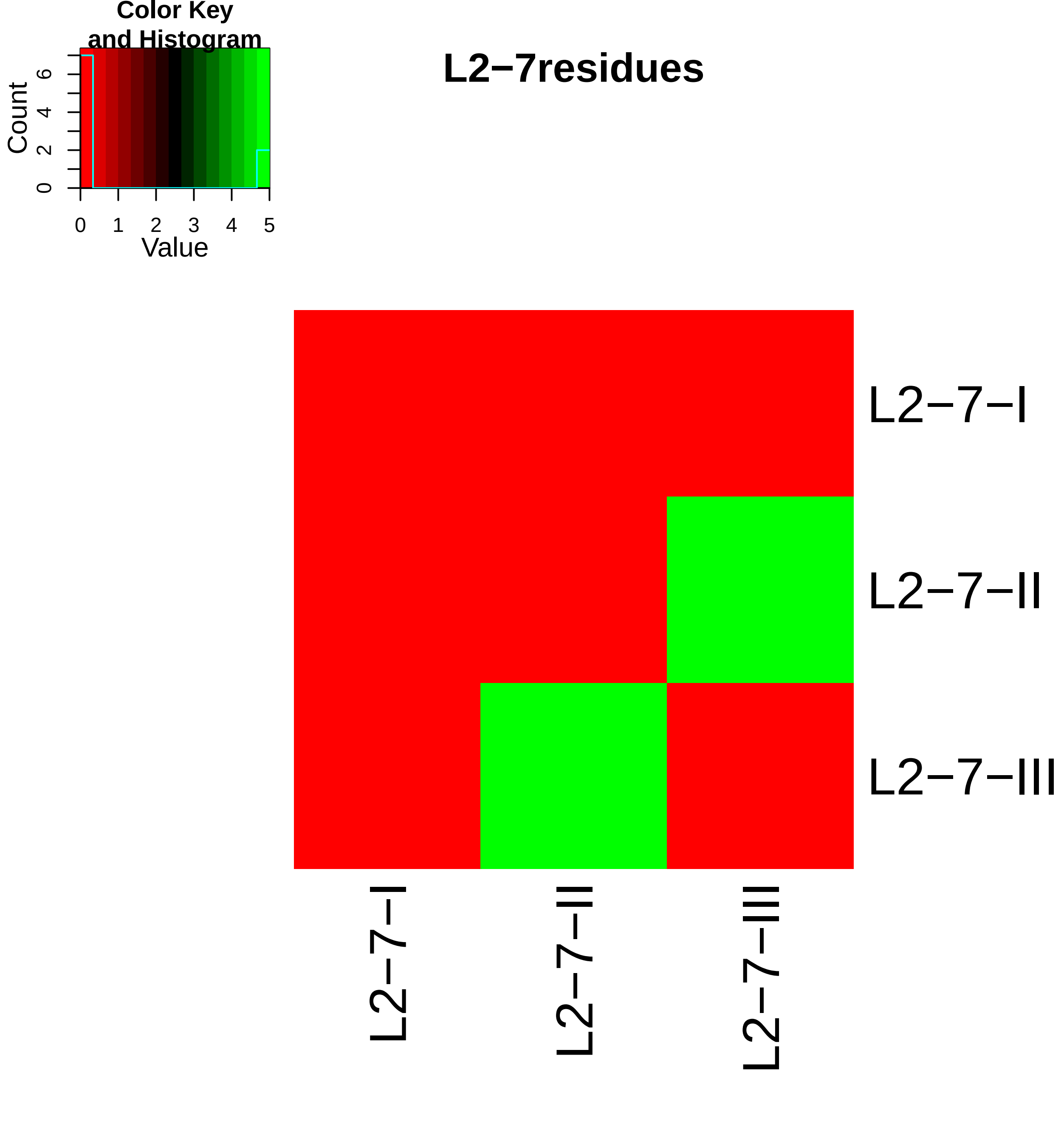

Color Key
and Histogram
Count
Value
0
2
4
6
0
1
2
3
4
5
L2−7residues
L2−7−I
L2−7−II
L2−7−III
L2−7−I
L2−7−II
L2−7−III

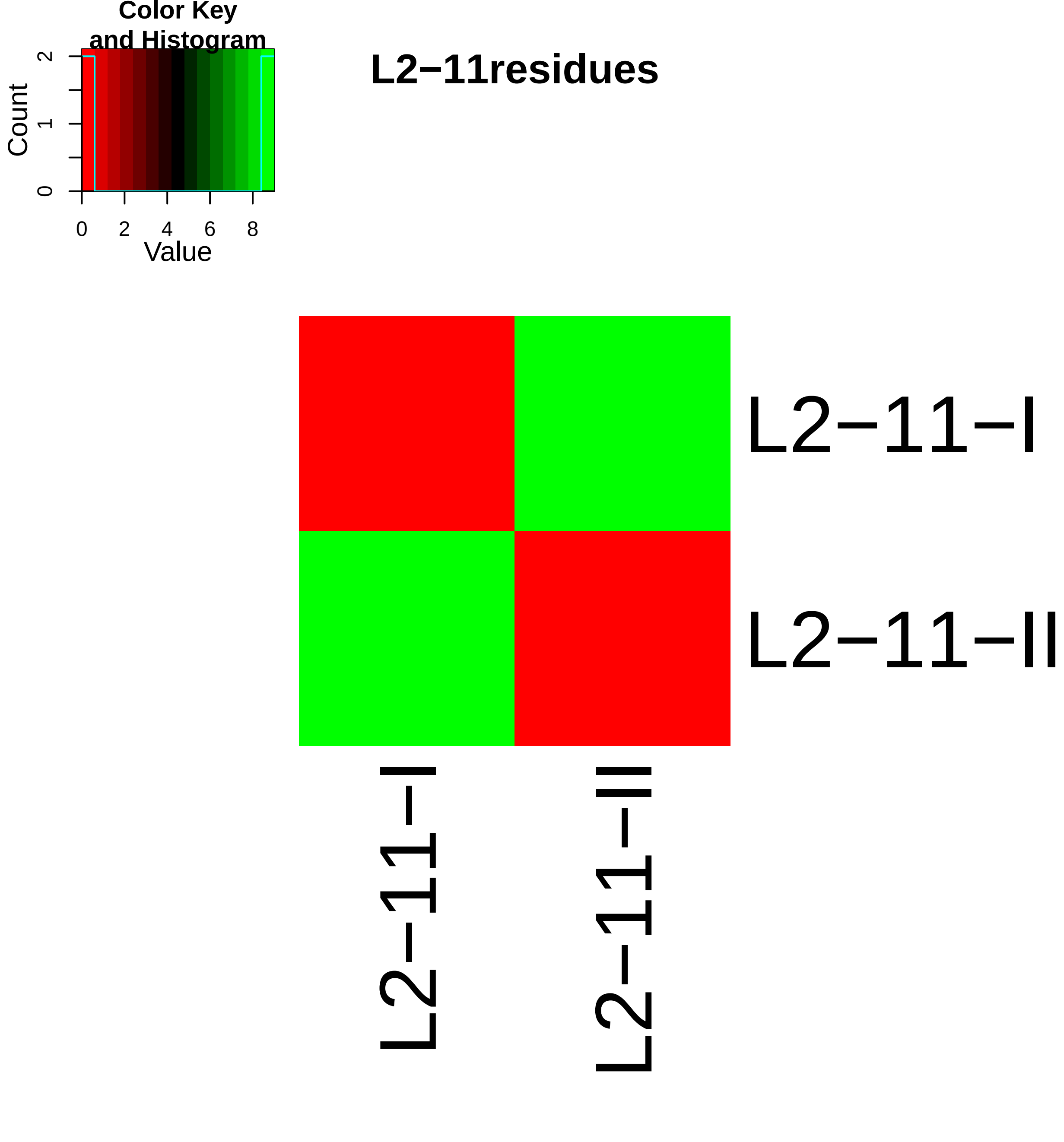
Color Key
and Histogram
Count
0
1
2
0
2
4
6
8
Value
L2−11residues
L2−11−I
L2−11−II
L2−11−I
L2−11−II

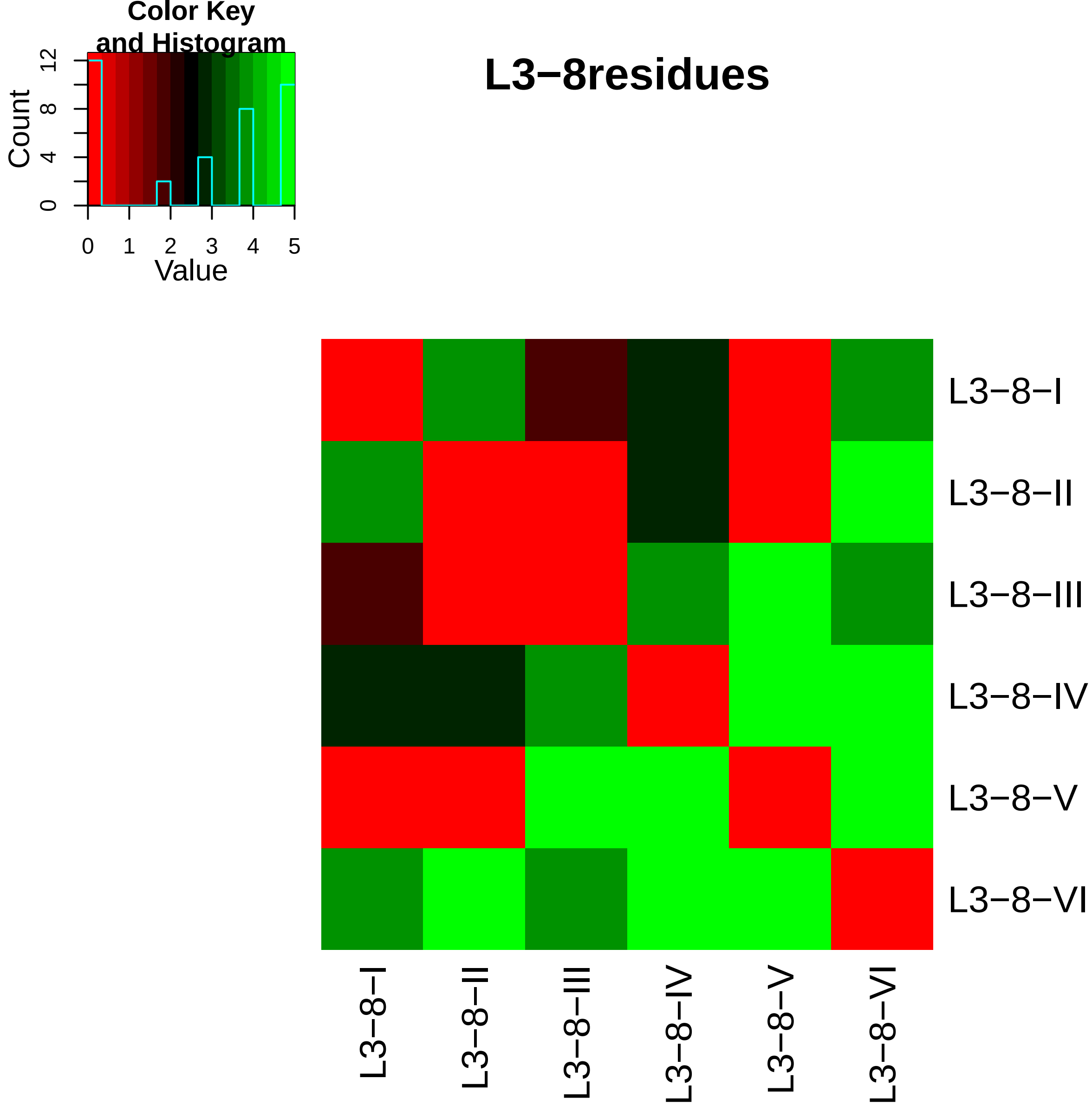
Color Key
and Histogram
Count
0
4
8
12
0
1
2
3
4
5
Value
L3−8residues
L3−8−I
L3−8−II
L3−8−III
L3−8−IV
L3−8−V
L3−8−VI
L3−8−I
L3−8−II
L3−8−III
L3−8−IV
L3−8−V
L3−8−VI

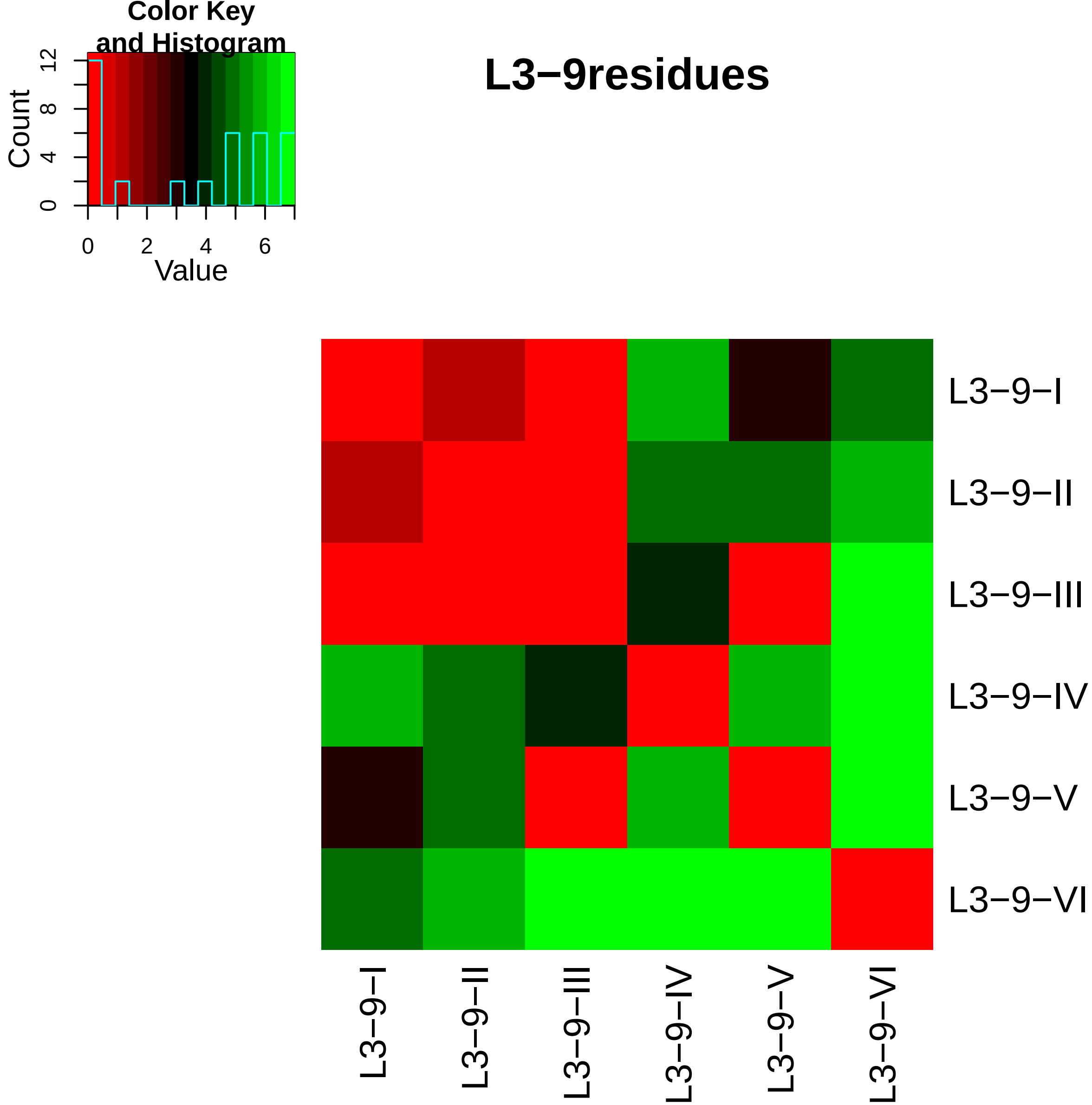
Color Key
and Histogram
Count
0
4
8
12
0
2
4
6
Value
L3−9residues
L3−9−I
L3−9−II
L3−9−III
L3−9−IV
L3−9−V
L3−9−VI
L3−9−I
L3−9−II
L3−9−III
L3−9−IV
L3−9−V
L3−9−VI

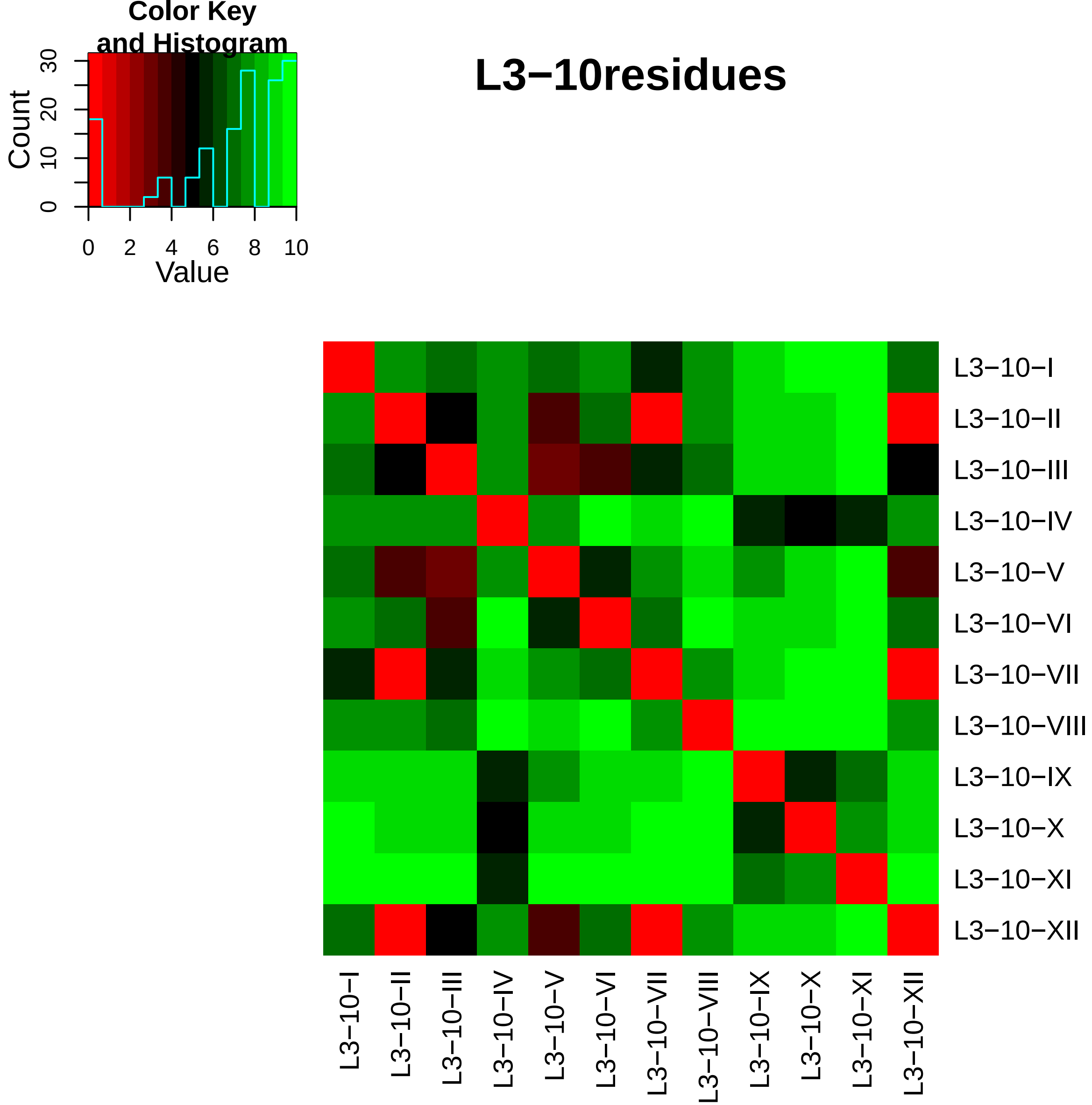

Color Key and Histogram
Count
0
10
20
30
0
2
4
6
8
10
Value
L3−10residues
L3−10−I
L3−10−II
L3−10−III
L3−10−IV
L3−10−V
L3−10−VI
L3−10−VII
L3−10−VIII
L3−10−IX
L3−10−X
L3−10−XI
L3−10−XII

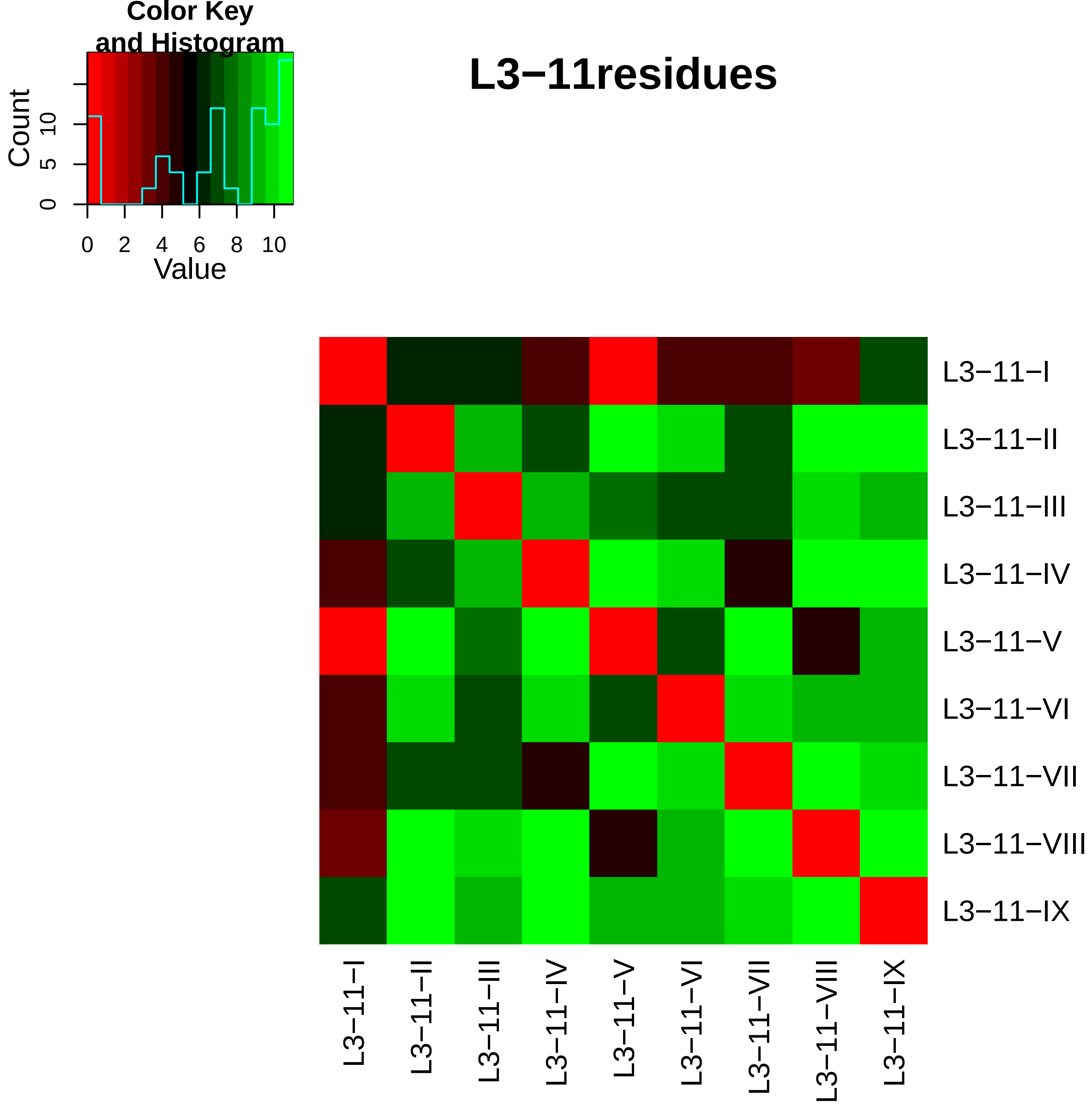

Color Key
and Histogram
Count
Value
0
5
10
0
2
4
6
8
10
L3−11residues
L3−11−I
L3−11−II
L3−11−III
L3−11−IV
L3−11−V
L3−11−VI
L3−11−VII
L3−11−VIII
L3−11−IX

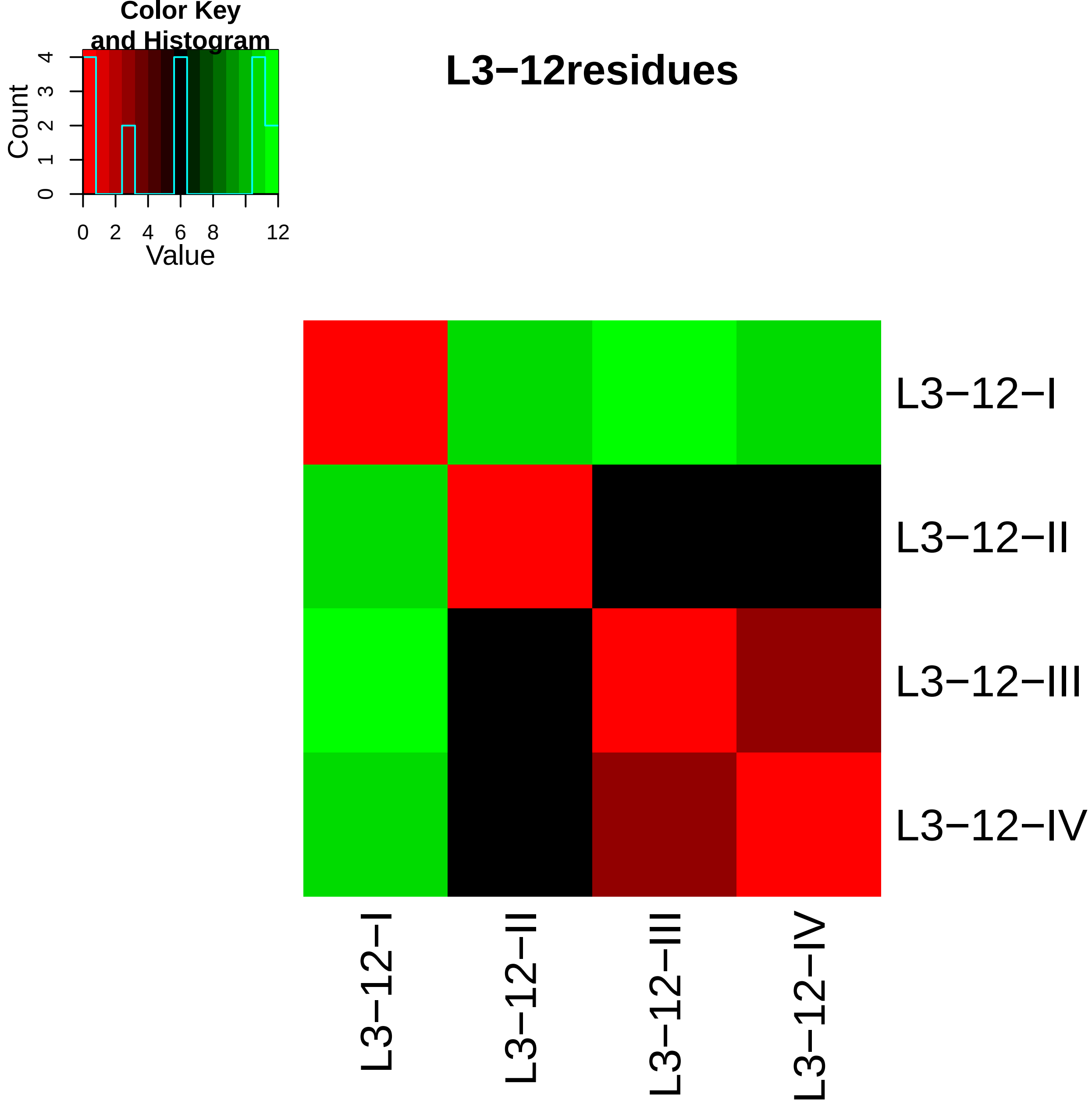
Color Key
and Histogram
Count
Value
0
1
2
3
4
0
2
4
6
8
12
L3−12residues
L3−12−I
L3−12−II
L3−12−III
L3−12−IV
L3−12−I
L3−12−II
L3−12−III
L3−12−IV

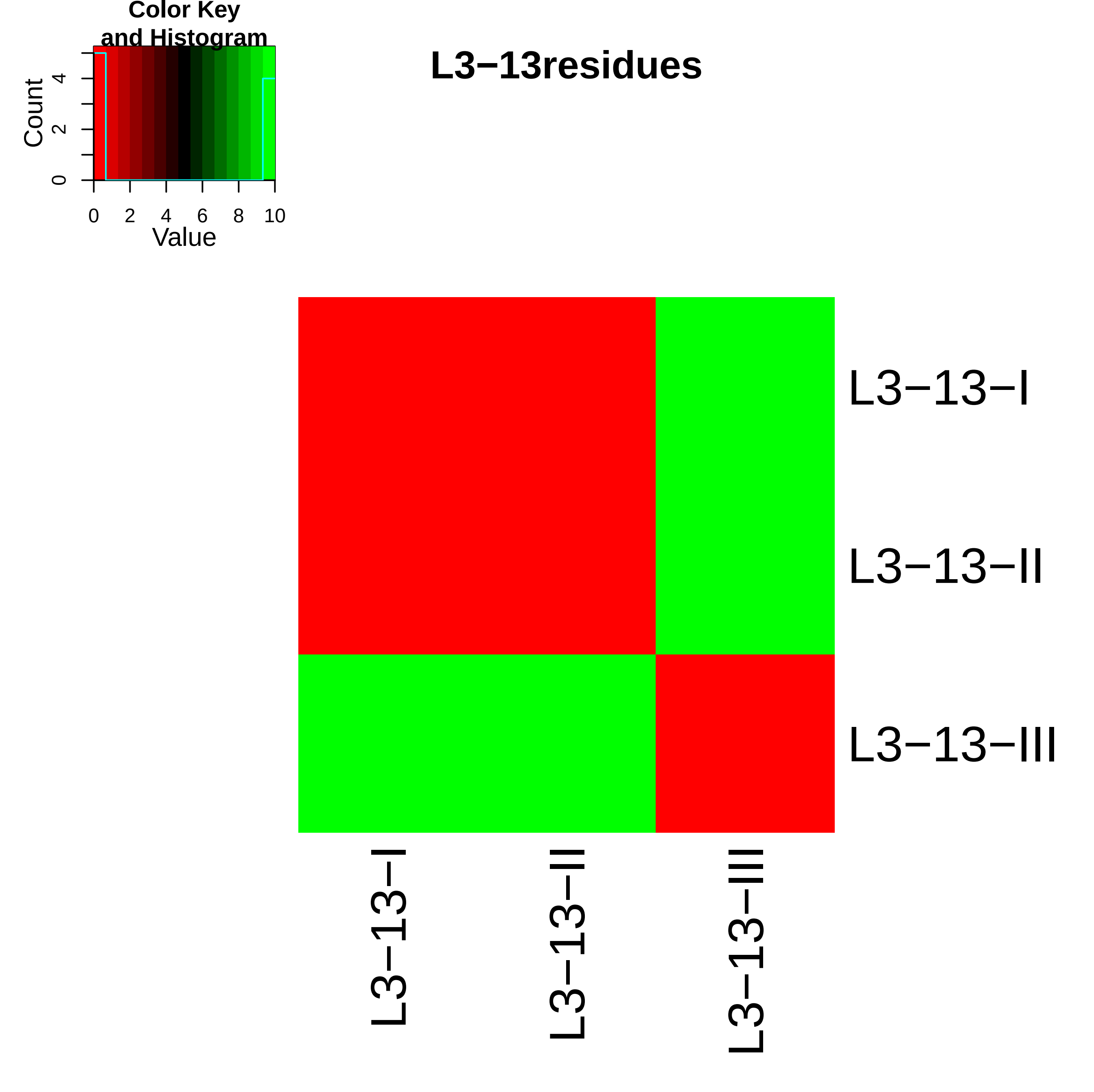
Color Key
and Histogram
Count
Value
0
2
4
6
8
10
L3−13residues
L3−13−I
L3−13−II
L3−13−III
L3−13−I
L3−13−II
L3−13−III
