# Supplementary material for: A complete, multi-level conformational clustering of antibody complementarity-determining regions
Source: PeerJ. 2014 Jul 1;2:e456. doi: 10.7717/peerj.456 (PMC4103072; doi:10.7717/peerj.456)
Supplement: Supplemental Information 4 — Level-2 clusters are shown exceptionally (marked with an asterisk) when no level-1 cluster is formed (minimum of 2 members required). [file peerj-02-456-s004.doc]

| **Cluster** | **Population** | **Unique sequences** | **Medoid PDB entry** | **Medoid Ramachandran conformation** | **Level-3 clusters** | **Level-2 clusters** | **Cluster diameter (Å)(Furthest members)** | **Best resolution in cluster (Å)(PDB)** | **Species in cluster** |
| --- | --- | --- | --- | --- | --- | --- | --- | --- | --- |
| ***CDR-H3 3-residues, Total population: 18, Total Unique sequences: 4, Clusters: 1, Outliers/Singletons: 0, Av.Silhouette: N/A*** | | | | | | | | | |
| H3-3-I | 18 | 4 (100%) | 1h8s_A | PLB | 4 | 2 | 0.21 (2Q8B_H-2Q8A_H) | 1.61 (1c5c_H) | HUMAN, MOUSE |
| ***CDR-H3 4-residues, Total population: 38, Total Unique sequences: 12, Clusters: 2, Outliers/Singletons: 2, Av.Silhouette: 0,56*** | | | | | | | | | |
| H3-4-I | 24 | 9 (75%) | 3MNZ_B | GAAB | 8 | 5 | 0.65 (2R4S_H-2ddq_H) | 1.49 (2VXT_H) | HUMAN, MOUSE |
| H3-4-II | 12 | 1 (8%) | 3BZ4_B | PDAB | 2 | 1 | 0.1 (3C6S_F-3BZ4_H) | 1.7 (3GGW_B) | MOUSE |
| ***CDR-H3 5-residues, Total population: 93, Total Unique sequences: 28, Clusters: 6, Outliers/Singletons: 8, Av.Silhouette: 0,68*** | | | | | | | | | |
| H3-5-I | 57 | 12 (43%) | 2dqc_H | AALAB | 6 | 3 | 1.21 (3DSF_H-1gpo_H) | 1.2 (3D9A_H) | HUMAN, MOUSE |
| H3-5-II | 18 | 8 (29%) | 1ktr_H | LLLAB | 6 | 4 | 0.91 (1gaf_H-1v7n_H) | 1.95 (1gaf_H) | HUMAN, MOUSE |
| H3-5-III | 4 | 2 (7%) | 1e4x_I | BBLPB | 2 | 2 | 0.77 (1e4x_H-1cu4_H) | 1.9 (1e4x_H) | MOUSE |
| H3-5-IV | 2 | 1 (4%) | 1ngx_B | ADBAB | 1 | 1 | 0.01 (1ngx_H-1ngx_B) | 1.8 (1ngx_B) | HUMAN, MOUSE |
| H3-5-V | 2 | 1 (4%) | 1ggb_H | LLLAB | 2 | 2 | 0.12 (1ggc_H-1ggb_H) | 2.8 (1ggb_H) | MOUSE |
| H3-5-VI | 2 | 1 (4%) | 1jnl_H | PBPBP | 2 | 2 | 0.23 (1jnl_H-1jnn_H) | 3.0 (1jnl_H) | MOUSE |
| ***CDR-H3 6-residues, Total population: 33, Total Unique sequences: 12, Clusters: 3, Outliers/Singletons: 3, Av.Silhouette: 0,72*** | | | | | | | | | |
| H3-6-I | 18 | 4 (33%) | 1ub5_H | APGPPB | 4 | 4 | 1.43 (2EH8_H-1f3d_H) | 1.6 (3CFB_H) | MOUSE |
| H3-6-II | 10 | 5 (42%) | 1za6_D | GABPAB | 5 | 4 | 1.28 (2EH7_H-1z3g_H) | 2.0 (2UZI_H) | HUMAN, MOUSE |
| H3-6-III | 2 | 1 (8%) | 1igy_B | PLLLAB | 1 | 1 | 0.01 (1igy_B-1igy_D) | 3.2 (1igy_B) | MOUSE |
| ***CDR-H3 7-residues, Total population: 97, Total Unique sequences: 41, Clusters: 7, Outliers/Singletons: 28, Av.Silhouette: 0,67*** | | | | | | | | | |
| H3-7-I | 12 | 5 (12%) | 3G5V_B | BDPGBAB | 8 | 4 | 0.89 (3G5Z_B-1fe8_J) | 1.59 (3G5Y_B) | MOUSE |
| H3-7-II | 7 | 2 (5%) | 1p2c_E | PDGPBAB | 3 | 1 | 0.38 (1mlc_D-1mlb_B) | 2.0 (1p2c_B) | MOUSE |
| H3-7-III | 4 | 1 (2%) | 3IET_B | BAAGLAB | 4 | 4 | 0.28 (3IF1_D-3IET_D) | 2.2 (3IET_B) | MOUSE |
| H3-7-IV | 3 | 1 (2%) | 3LEX_A | BBAPPDB | 2 | 1 | 0.12 (3LEY_H-3LEX_H) | 1.97 (3LEX_H) | MOUSE |
| H3-7-V | 2 | 1 (2%) | 2ZPK_H | PBPGBAB | 1 | 1 | 0.15 (2ZPK_I-2ZPK_H) | 1.8 (2ZPK_H) | MOUSE |
| H3-7-VI | 32 | 13 (32%) | 3FO9_H | BBADBBB | 16 | 8 | 1.68 (1YQV_H-1mjj_B) | 1.22 (1mju_H) | HUMAN, MOUSE |
| H3-7-VII | 9 | 4 (10%) | 1mre_H | BBGABBB | 7 | 6 | 1.06 (1h3p_H-1dzb_B) | 2.0 (1dzb_A) | MOUSE |
| ***CDR-H3 8-residues, Total population: 168, Total Unique sequences: 46, Clusters: 7, Outliers/Singletons: 27, Av.Silhouette: 0,57*** | | | | | | | | | |
| H3-8-I | 19 | 1 (2%) | 1vhp_5 | BDGLDBLB | 16 | 15 | 1.2 (1vhp_b-1vhp_6) | N/A | HUMAN |
| **Cluster** | **Population** | **Unique sequences** | **Medoid PDB entry** | **Medoid Ramachandran conformation** | **Level-3 clusters** | **Level-2 clusters** | **Cluster diameter (Å)(Furthest members)** | **Best resolution in cluster (Å)(PDB)** | **Species in cluster** |
| H3-8-II | 98 | 27 (59%) | 1g7h_B | BBLLBPAB | 52 | 13 | 1.81 (2R0L_H-1cic_B) | 1.5 (1a2y_B) | HUMAN, MOUSE, LLAMA, HAMSTER, SYNTHETIC HUMANIZED ANTIBODY |
| H3-8-III | 8 | 3 (7%) | 1uz6_P | LPLBAAPB | 3 | 2 | 1.12 (1uz8_H-1mam_H) | 1.8 (1uz8_B) | MOUSE |
| H3-8-IV | 6 | 4 (9%) | 1fl6_B | BBADGPAB | 4 | 3 | 0.82 (2HKF_H-1kel_H) | 1.85 (2g60_H) | HUMAN, MOUSE |
| H3-8-V | 5 | 2 (4%) | 1uwe_Y | BBGDABBB | 3 | 2 | 0.8 (1uwg_Y-1uwe_H) | 2.67 (1uwe_H) | HUMAN, MOUSE |
| H3-8-VI | 3 | 1 (2%) | 1qbm_H | BDDBAPBB | 2 | 2 | 0.18 (1wej_H-1qbl_H) | 1.8 (1wej_H) | MOUSE |
| H3-8-VII | 2 | 1 (2%) | 1i7z_B | BDBAABBB | 2 | 1 | 0.11 (1i7z_D-1i7z_B) | 2.3 (1i7z_B) | CHIMERA OF MOUSE/HUMAN |
| ***CDR-H3 9-residues, Total population: 181, Total Unique sequences: 55, Clusters: 8, Outliers/Singletons: 49, Av.Silhouette: 0,55*** | | | | | | | | | |
| H3-9-I | 40 | 1 (2%) | 2ITD_A | BALDLBPAB | 4 | 1 | 0.25 (3OGC_A-2bob_A) | 1.72 (2IH3_A) | MOUSE |
| H3-9-II | 32 | 11 (20%) | 2OTU_F | PBGDPABAB | 17 | 10 | 1.63 (1mfa_1-1jv5_B) | 1.5 (2W60_A) | HUMAN, MOUSE |
| H3-9-III | 31 | 9 (16%) | 1mhh_B | BBAALBPAB | 17 | 2 | 1.34 (3LS5_H-1pz5_B) | 1.6 (1nlb_H) | HUMAN, MOUSE |
| H3-9-IV | 18 | 8 (15%) | 2QR0_F | PPPLGGBDB | 11 | 6 | 1.39 (3IY4_B-1ORS_B) | 1.28 (1mqk_H) | HUMAN, MOUSE |
| H3-9-V | 4 | 1 (2%) | 3CFI_C | BAGGDBBBL | 3 | 2 | 0.22 (3CFI_I-3CFI_F) | 2.58 (3CFI_C) | LLAMA |
| H3-9-VI | 3 | 2 (4%) | 2arj_H | BBLBDBPAB | 2 | 2 | 1.07 (3K2U_H-2arj_H) | 2.35 (3K2U_H) | HUMAN, RAT |
| H3-9-VII | 2 | 1 (2%) | 1t4k_B | BBABGPGPB | 1 | 1 | 0.07 (1t4k_D-1t4k_B) | 2.5 (1t4k_B) | MOUSE |
| H3-9-VIII | 2 | 1 (2%) | 3B9K_H | BADBDPPDB | 2 | 1 | 0.11 (3B9K_D-3B9K_H) | 2.7 (3B9K_H) | RAT |
| ***CDR-H3 10-residues, Total population: 377, Total Unique sequences: 98, Clusters: 35, Outliers/Singletons: 85, Av.Silhouette: 0,67*** | | | | | | | | | |
| H3-10-I | 79 | 3 (3%) | 2KH2_B | BABAPPBBAB | 45 | 4 | 1.06 (2KH2_B_59-2KH2_B_9) | 1.8 (1l7i_H) | HUMAN, MOUSE |
| H3-10-II | 20 | 1 (1%) | 2VDQ_H | PADPAAPBDB | 6 | 1 | 0.15 (3NIG_H-1ty7_H) | 2.25 (3NIG_E) | MOUSE |
| H3-10-III | 19 | 8 (8%) | 1dbk_H | PPAAALBPDB | 15 | 3 | 1.06 (1jgv_H-1egj_H) | 1.8 (1jgu_H) | HUMAN, MOUSE |
| H3-10-IV | 11 | 1 (1%) | 1ruk_H | BPAADLPLPB | 1 | 1 | 0.28 (1rul_H-1nd0_B) | 1.3 (1ncw_H) | MOUSE |
| H3-10-V | 10 | 4 (4%) | 1s3k_H | PBAALBABAB | 8 | 5 | 0.75 (2cjv_H-1cly_H) | 1.9 (1s3k_H) | MOUSE, CHIMERA OF MOUSE/HUMAN |
| **Cluster** | **Population** | **Unique sequences** | **Medoid PDB entry** | **Medoid Ramachandran conformation** | **Level-3 clusters** | **Level-2 clusters** | **Cluster diameter (Å)(Furthest members)** | **Best resolution in cluster (Å)(PDB)** | **Species in cluster** |
| H3-10-VI | 8 | 1 (1%) | 2gfb_L | ADLPPBPBPA | 2 | 1 | 0.19 (2gfb_F-2gfb_B) | 3.0 (2gfb_B) | MOUSE |
| H3-10-VII | 8 | 3 (3%) | 1lo0_Y | BBPLDBABAB | 3 | 2 | 0.8 (2HWZ_H-1lo2_Y) | 1.8 (2HWZ_H) | MOUSE |
| H3-10-VIII | 8 | 2 (2%) | 3DV6_B | BBAADLBPAB | 4 | 2 | 0.29 (3DUU_B-3DUR_D) | 1.86 (3DUR_B) | MOUSE |
| H3-10-IX | 8 | 1 (1%) | 2ajx_H | BBAALPDGDP | 5 | 1 | 0.46 (2ajv_H-2ajs_H) | 1.5 (2aju_H) | MOUSE |
| H3-10-X | 8 | 1 (1%) | 43c9_F | BBPLBABABB | 6 | 2 | 0.59 (43ca_H-43ca_B) | 2.2 (43c9_B) | MOUSE |
| H3-10-XI | 8 | 1 (1%) | 3HAE_T | BABPLAGPDB | 4 | 1 | 0.81 (3HAE_H-3GJE_H) | 1.9 (3GJF_H) | HUMAN |
| H3-10-XII | 7 | 2 (2%) | 2I9L_F | PPAALDBPAB | 4 | 3 | 0.67 (2igf_H-2I9L_B) | 2.8 (1igf_H) | MOUSE |
| H3-10-XIII | 7 | 3 (3%) | 3QPQ_J | PPAPAABBAB | 5 | 2 | 0.61 (3QPQ_D-3L95_B) | 1.9 (3QPQ_H) | HUMAN |
| H3-10-XIV | 6 | 1 (1%) | 2X7L_G | BADLDLBPAB | 1 | 1 | 0.09 (2X7L_H-2X7L_A) | 3.17 (2X7L_A) | N/A |
| H3-10-XV | 6 | 2 (2%) | 1rz7_H | BADLALBPAB | 3 | 2 | 0.61 (3JWO_H-1f11_B) | 2.0 (1rz7_H) | HUMAN, MOUSE |
| H3-10-XVI | 6 | 1 (1%) | 1bfo_B | AGBGPBPLAB | 3 | 3 | 0.61 (1ce1_H-1bey_H) | 1.9 (1ce1_H) | HUMAN, RAT |
| H3-10-XVII | 6 | 1 (1%) | 3ETB_G | BBLLADBPAB | 2 | 2 | 0.69 (3ESU_F-3ETB_H) | 1.3 (3ESU_F) | MOUSE |
| H3-10-XVIII | 6 | 2 (2%) | 2UYL_B | PPPLDBABAB | 2 | 1 | 0.52 (1bln_D-1bln_B) | 2.5 (2UYL_B) | MOUSE |
| H3-10-XIX | 6 | 3 (3%) | 3QWO_A | BBAADLBPAB | 2 | 2 | 0.76 (3IXT_H-3IFL_H) | 1.5 (3IFL_H) | MOUSE |
| H3-10-XX | 5 | 1 (1%) | 3IXX_I | not available | 2 | 2 | 0.53 (3IXY_I-3I50_H) | 3.0 (3I50_H) | MOUSE |
| H3-10-XXI | 5 | 1 (1%) | 2b2x_I | PBGALABPAB | 4 | 1 | 0.37 (3EOT_H-1mhp_H) | 1.9 (3EOT_H) | MOUSE |
| H3-10-XXII | 5 | 2 (2%) | 3OPZ_I | PPAPLLPPAP | 2 | 1 | 0.61 (3OPZ_J-3DGG_B) | 2.3 (3DGG_B) | MOUSE |
| H3-10-XXIII | 5 | 1 (1%) | 3O41_H | BBGAAAGPAB | 2 | 1 | 0.21 (3QRG_H-3O45_H) | 1.7 (3QRG_H) | HUMAN, MOUSE |
| H3-10-XXIV | 5 | 1 (1%) | 1jnh_B | BABAABBLAB | 3 | 1 | 0.28 (1jnh_F-1jn6_B) | 2.7 (1jn6_B) | MOUSE |
| H3-10-XXV | 4 | 1 (1%) | 1aif_H | BPAPABBBPB | 2 | 2 | 0.84 (3IY5_B-1iai_I) | 2.9 (1aif_H) | MOUSE |
| H3-10-XXVI | 4 | 2 (2%) | 1riu_H | BABPLAGPAB | 2 | 2 | 0.59 (2agj_H-1qyg_H) | 1.81 (1qyg_H) | HUMAN, MOUSE |
| H3-10-XXVII | 4 | 1 (1%) | 2V7N_F | BDGAALGPAB | 3 | 1 | 0.15 (2V7N_H-2V7N_B) | 1.92 (2V7N_B) | HUMAN |
| H3-10-XXVIII | 3 | 1 (1%) | 1kno_B | BLAPLAGPBB | 2 | 1 | 0.14 (1kno_F-1kno_D) | 3.2 (1kno_B) | MOUSE |
| H3-10-XXIX | 3 | 1 (1%) | 1ruq_H | PABPLDGPAB | 2 | 2 | 0.28 (1rur_H-1a3l_H) | 1.5 (1rur_H) | MOUSE |
| H3-10-XXX | 2 | 1 (1%) | 3RPI_H | PAPADLPBAB | 1 | 1 | 0.0 (3RPI_A-3RPI_H) | 2.65 (3RPI_H) | HUMAN |
| H3-10-XXXI | 2 | 1 (1%) | 3DIF_B | PBPGDPPLAB | 1 | 1 | 0.08 (3DIF_B-3DIF_D) | 2.4 (3DIF_B) | HUMAN |
| **Cluster** | **Population** | **Unique sequences** | **Medoid PDB entry** | **Medoid Ramachandran conformation** | **Level-3 clusters** | **Level-2 clusters** | **Cluster diameter (Å)(Furthest members)** | **Best resolution in cluster (Å)(PDB)** | **Species in cluster** |
| H3-10-XXXII | 2 | 1 (1%) | 1mh5_B | BPADPAABBB | 1 | 1 | 0.14 (1mh5_H-1mh5_B) | 2.1 (1mh5_B) | MOUSE |
| H3-10-XXXIII | 2 | 1 (1%) | 25c8_H | PPABPLBPPB | 1 | 1 | 0.17 (25c8_H-35c8_H) | 2.0 (25c8_H) | MOUSE |
| H3-10-XXXIV | 2 | 1 (1%) | 3GKZ_A | BBAABAABBB | 1 | 1 | 0.18 (3GM0_A-3GKZ_A) | 1.9 (3GKZ_A) | MOUSE |
| H3-10-XXXV | 2 | 1 (1%) | 1YC7_A | BBGDABBBBB | 1 | 1 | 0.27 (1YC7_B-1YC7_A) | 1.6 (1YC7_A) | CAMEL |
| ***CDR-H3 11-residues, Total population: 231, Total Unique sequences: 64, Clusters: 26, Outliers/Singletons: 80, Av.Silhouette: 0,70*** | | | | | | | | | |
| H3-11-I | 22 | 2 (3%) | 2R1Y_B | BPDLDPBBBAB | 8 | 1 | 0.6 (3T77_B-2R2E_B) | 1.45 (1q9r_B) | MOUSE |
| H3-11-II | 16 | 3 (5%) | 1oar_H | BPDBLBBBPDP | 8 | 4 | 1.09 (2Y36_H-1oay_J) | 1.8 (1oau_H) | HUMAN, RAT, MOUSE |
| H3-11-III | 15 | 7 (11%) | 1ngp_H | BPAABALBPAB | 14 | 7 | 1.34 (1rjl_B-1a6v_H) | 1.8 (1a6v_H) | MOUSE, SYNTHETIC HUMANIZED ANTIBODY |
| H3-11-IV | 14 | 5 (8%) | 3MBX_H | PABADBBBBAB | 11 | 3 | 1.22 (3L5Y_H-3IY0_H) | 1.6 (3MBX_H) | HUMAN, MOUSE |
| H3-11-V | 12 | 1 (2%) | 2fjf_F | BBAPPALBPAB | 1 | 1 | 0.14 (2fjf_R-2fjf_H) | 2.65 (2fjf_H) | HUMAN |
| H3-11-VI | 9 | 1 (2%) | 3B2U_H | BPADPALBPDP | 9 | 2 | 0.48 (3B2V_H-3B2U_Q) | 2.58 (3B2U_H) | HUMAN |
| H3-11-VII | 8 | 3 (5%) | 3NH7_I | PBBDABPBPAB | 5 | 2 | 1.08 (2mcp_H-1e6j_H) | 1.8 (1e6o_H) | HUMAN, MOUSE |
| H3-11-VIII | 6 | 1 (2%) | 3EO1_B | PADPABBPBDB | 3 | 3 | 0.48 (3EO1_E-3EO0_B) | 1.75 (3EO0_B) | HUMAN |
| H3-11-IX | 6 | 2 (3%) | 1nc4_D | BPPPADPBPAB | 3 | 2 | 0.58 (3H3B_D-1nc4_B) | 2.1 (1nc2_B) | MOUSE |
| H3-11-X | 4 | 1 (2%) | 3CFE_B | BPPGGALBPAB | 2 | 1 | 0.16 (3CFE_H-3CFD_B) | 2.5 (3CFD_H) | MOUSE |
| H3-11-XI | 4 | 1 (2%) | 2fl5_B | PPDBGPBPPDB | 3 | 2 | 0.12 (2fl5_F-2fl5_H) | 3.0 (2fl5_H) | HUMAN |
| H3-11-XII | 4 | 1 (2%) | 1jp5_A | PDAADLPBLAB | 4 | 2 | 0.32 (1svz_A-1jp5_B) | 1.89 (1svz_A) | MOUSE |
| H3-11-XIII | 4 | 1 (2%) | 1nca_H | PABPLLABPPL | 4 | 1 | 0.28 (1ncc_H-1ncb_H) | 2.5 (1nca_H) | MOUSE |
| H3-11-XIV | 3 | 1 (2%) | 2adg_B | BAPABBBPBAB | 3 | 2 | 0.24 (2adj_B-2adi_B) | 2.5 (2adg_B) | MOUSE |
| H3-11-XV | 2 | 1 (2%) | 3NCY_Q | PAGLPGDBPAB | 1 | 1 | 0.00 (3NCY_P-3NCY_Q) | 3.2 (3NCY_Q) | MOUSE |
| H3-11-XVI | 2 | 1 (2%) | 3UBX_H | BPGDADPBPAB | 1 | 1 | 0.01 (3UBX_G-3UBX_H) | 3.1 (3UBX_H) | MOUSE |
| H3-11-XVII | 2 | 1 (2%) | 1afv_H | BPAPAPPBPAB | 2 | 1 | 0.07 (1afv_K-1afv_H) | 3.7 (1afv_H) | MOUSE |
| H3-11-XVIII | 2 | 1 (2%) | 2h9g_B | PABADPAPBBL | 2 | 1 | 0.1 (2h9g_B-2h9g_H) | 2.32 (2h9g_B) | HUMAN |
| H3-11-XIX | 2 | 1 (2%) | 1hil_B | PPPLLAGGBBL | 1 | 1 | 0.11 (1hil_B-1hil_D) | 2.0 (1hil_B) | MOUSE |
| H3-11-XX | 2 | 1 (2%) | 1ken_H | BAGDLDBBBBB | 1 | 1 | 0.12 (1ken_H-1ken_T) | 3.5 (1ken_H) | MOUSE |
| **Cluster** | **Population** | **Unique sequences** | **Medoid PDB entry** | **Medoid Ramachandran conformation** | **Level-3 clusters** | **Level-2 clusters** | **Cluster diameter (Å)(Furthest members)** | **Best resolution in cluster (Å)(PDB)** | **Species in cluster** |
| H3-11-XXI | 2 | 1 (2%) | 1n4x_H | PADLDLABPAB | 2 | 1 | 0.12 (1n4x_H-1n4x_I) | 1.7 (1n4x_H) | MOUSE |
| H3-11-XXII | 2 | 1 (2%) | 3HI6_H | PPLGDPABBAB | 2 | 1 | 0.14 (3HI6_H-3HI6_X) | 2.3 (3HI6_H) | HUMAN |
| H3-11-XXIII | 2 | 2 (3%) | 1fvd_D | PPLLLAAGPAB | 2 | 1 | 0.17 (1fve_D-1fvd_D) | 2.5 (1fvd_D) | HUMAN |
| H3-11-XXIV | 2 | 1 (2%) | 1i8m_B | PBLPDALBPDB | 2 | 1 | 0.21 (1xf2_B-1i8m_B) | 2.1 (1i8m_B) | MOUSE |
| H3-11-XXV | 2 | 1 (2%) | 3NZ8_A | BPBPLBDBPAB | 2 | 1 | 0.24 (3NZ8_H-3NZ8_A) | 2.7 (3NZ8_A) | MOUSE |
| H3-11-XXVI | 2 | 1 (2%) | 12e8_H | BPAAPALPBAB | 1 | 1 | 0.29 (12e8_P-12e8_H) | 1.9 (12e8_H) | MOUSE |
| ***CDR-H3 12-residues, Total population: 206, Total Unique sequences: 51, Clusters: 21, Outliers/Singletons: 32, Av.Silhouette: 0,81*** | | | | | | | | | |
| H3-12-I | 46 | 3 (6%) | 2esg_A | not available | 5 | 3 | 0.87 (1igm_H-2esg_A) | 2.0 (1vge_H) | HUMAN, MOUSE |
| H3-12-II | 42 | 1 (2%) | 2exy_E | BBBDALPPBPAB | 10 | 2 | 0.57 (2H2S_C-2FEC_I) | 2.51 (1OTS_C) | HUMAN, MOUSE |
| H3-12-III | 17 | 3 (6%) | 2P49_B | LBBGDPAAAPDB | 3 | 1 | 0.38 (2P4A_B-2P47_B) | 1.1 (2P45_B) | DROMEDARY, CAMEL |
| H3-12-IV | 11 | 3 (6%) | 2a6d_B | BBAGLGBPBPAB | 11 | 6 | 1.09 (2a6j_B-1iqw_H) | 1.9 (1jfq_H) | MOUSE, CHIMERA OF MOUSE/HUMAN |
| H3-12-V | 6 | 3 (6%) | 3NAA_H | PBPBLLBPBPAB | 2 | 1 | 0.59 (3NCJ_H-1iai_H) | 1.6 (3NCJ_H) | MOUSE |
| H3-12-VI | 5 | 1 (2%) | 1dee_B | PABPBAAPPPAB | 3 | 2 | 0.41 (1hez_D-1dee_F) | 2.7 (1dee_B) | HUMAN |
| H3-12-VII | 5 | 2 (4%) | 3EZJ_H | PBPPAALPBBBB | 4 | 1 | 0.66 (3EZJ_D-3C08_H) | 2.15 (3C08_H) | HUMAN, MOUSE |
| H3-12-VIII | 5 | 1 (2%) | 3EOA_H | BBDABGABBBDB | 5 | 2 | 0.41 (3EOB_H-3EO9_H) | 1.8 (3EO9_H) | HUMAN |
| H3-12-IX | 4 | 1 (2%) | 1qlr_B | PABADBPPBBDB | 3 | 2 | 0.37 (1qlr_D-1dn0_D) | 2.28 (1dn0_B) | HUMAN |
| H3-12-X | 4 | 1 (2%) | 3P0V_H | PPABGAPAPBDB | 4 | 3 | 0.62 (3P0Y_H-3P0V_H) | 1.8 (3P0Y_H) | HUMAN |
| H3-12-XI | 4 | 1 (2%) | 2g5b_H | BPAPLLALBPAB | 2 | 1 | 0.13 (2g5b_F-2g5b_B) | 2.3 (2g5b_B) | MOUSE |
| H3-12-XII | 4 | 1 (2%) | 1j05_B | BBAADBAABBAB | 4 | 3 | 0.72 (1MOE_B-1j05_H) | 1.5 (1j05_H) | MOUSE |
| H3-12-XIII | 4 | 1 (2%) | 3NGB_H | PABAABAAPPAB | 3 | 1 | 0.16 (3NGB_E-3NGB_B) | 2.68 (3NGB_H) | HUMAN |
| H3-12-XIV | 3 | 1 (2%) | 3GBM_H | PABAPPBABBAB | 2 | 1 | 0.19 (3GBN_H-3GBM_I) | 2.2 (3GBN_H) | HUMAN |
| H3-12-XV | 2 | 1 (2%) | 2R56_H | PABAPPALBBAB | 1 | 1 | 0.04 (2R56_I-2R56_H) | 2.8 (2R56_I) | HUMAN |
| H3-12-XVI | 2 | 1 (2%) | 1OL0_A | PPPPDPPAAAPB | 1 | 1 | 0.09 (1OL0_A-1OL0_B) | 1.8 (1OL0_A) | HUMAN |
| H3-12-XVII | 2 | 1 (2%) | 3BGF_H | BBPPAADABLAB | 1 | 1 | 0.11 (3BGF_B-3BGF_H) | 3.0 (3BGF_H) | MOUSE |
| H3-12-XVIII | 2 | 1 (2%) | 2fjh_H | BBAABPPLPPAB | 1 | 1 | 0.14 (2fjh_H-2fjh_B) | 3.1 (2fjh_H) | HUMAN |
| **Cluster** | **Population** | **Unique sequences** | **Medoid PDB entry** | **Medoid Ramachandran conformation** | **Level-3 clusters** | **Level-2 clusters** | **Cluster diameter (Å)(Furthest members)** | **Best resolution in cluster (Å)(PDB)** | **Species in cluster** |
| H3-12-XIX | 2 | 1 (2%) | 1kfa_H | PAPAABGPBPAB | 2 | 1 | 0.18 (1kfa_H-1kfa_I) | 2.8 (1kfa_H) | HUMAN |
| H3-12-XX | 2 | 1 (2%) | 3MA9_H | BLABADPAPPAB | 2 | 1 | 0.19 (3MA9_H-3MAC_H) | 2.05 (3MA9_H) | HUMAN |
| H3-12-XXI | 2 | 1 (2%) | 3C09_H | BBBPAALPPBBB | 1 | 1 | 0.25 (3C09_H-3C09_C) | 3.2 (3C09_H) | HUMAN, MOUSE |
| ***CDR-H3 13-residues, Total population: 130, Total Unique sequences: 42, Clusters: 22, Outliers/Singletons: 25, Av.Silhouette: 0,84*** | | | | | | | | | |
| H3-13-I | 20 | 1 (2%) | 3RIA_F | PAPPLBPDLBPPB | 2 | 2 | 0.07 (3RIF_I-3RHW_I) | 3.26 (3RHW_F) | MOUSE |
| H3-13-II | 11 | 2 (5%) | 2IBZ_X | BPBBAAAGPBPAB | 7 | 3 | 1.54 (3CX5_J-2X1O_A) | 1.34 (2X1O_A) | MOUSE, LLAMA |
| H3-13-III | 8 | 1 (2%) | 3Q3G_K | BPBDLBPGBPPAB | 3 | 1 | 0.15 (3QA3_D-3Q3G_B) | 2.7 (3Q3G_D) | HUMAN |
| H3-13-IV | 8 | 1 (2%) | 1t03_H | PBPABADBBBPAB | 6 | 1 | 0.52 (1r0a_H-1j5o_H) | 2.8 (1r0a_H) | MOUSE |
| H3-13-V | 7 | 2 (5%) | 2IPU_G | BPDBAAALPBPAB | 6 | 2 | 0.42 (2R0W_H-2IPT_H) | 1.65 (2IPU_G) | MOUSE |
| H3-13-VI | 6 | 1 (2%) | 3FO2_H | PBGGDABPABBAB | 4 | 2 | 0.91 (3FO1_B-2GK0_H) | 2.18 (3FO2_H) | HUMAN, MOUSE |
| H3-13-VII | 5 | 1 (2%) | 1nmc_H | PAAGAPDBLGPAB | 4 | 3 | 0.65 (1nma_H-1a14_H) | 2.5 (1a14_H) | MOUSE |
| H3-13-VIII | 5 | 1 (2%) | 3QCU_H | DBBPPLALPBBBP | 2 | 1 | 0.69 (3QCU_I-3QCT_H) | 1.98 (3QCU_H) | HUMAN |
| H3-13-IX | 4 | 1 (2%) | 1q9w_D | BPDBAAABBGPAB | 3 | 1 | 0.35 (1q9o_D-1q9o_B) | 1.75 (1q9w_B) | MOUSE |
| H3-13-X | 4 | 2 (5%) | 3G6A_H | PABDBAAAAPPDB | 4 | 2 | 1.24 (3O2D_H-3G6D_H) | 2.1 (3G6A_H) | HUMAN, MOUSE |
| H3-13-XI | 4 | 1 (2%) | 1lmk_A | BPPAALDBBBPAB | 4 | 1 | 0.44 (1lmk_E-1lmk_C) | 2.6 (1lmk_A) | MOUSE |
| H3-13-XII | 3 | 1 (2%) | 2ZCL_H | BPBGPLDLLPBAB | 2 | 1 | 0.19 (2ZCK_H-2ZCH_H) | 2.83 (2ZCH_H) | MOUSE |
| H3-13-XIII | 2 | 1 (2%) | 3IVK_H | PPPAAADGDPPAB | 1 | 1 | 0.01 (3IVK_H-3IVK_A) | 3.1 (3IVK_H) | MOUSE |
| H3-13-XIV | 2 | 1 (2%) | 1xiw_D | BPABAADAABPAB | 2 | 1 | 0.08 (1xiw_D-1xiw_H) | 1.9 (1xiw_D) | MOUSE |
| H3-13-XV | 2 | 1 (2%) | 3I02_B | PPDBAADGLBPAB | 2 | 1 | 0.15 (3I02_B-3I02_D) | 2.6 (3I02_B) | MOUSE |
| H3-13-XVI | 2 | 1 (2%) | 2J6E_H | PGAPPLPDDBBAB | 2 | 1 | 0.17 (2J6E_H-2J6E_I) | 3.0 (2J6E_H) | HUMAN |
| H3-13-XVII | 2 | 1 (2%) | 2YK1_H | PDGPDPAPBLPBB | 2 | 1 | 0.17 (2YK1_H-2YKL_H) | 1.85 (2YK1_H) | HUMAN |
| H3-13-XVIII | 2 | 1 (2%) | 3KDM_H | PAPADBPALBBDP | 2 | 1 | 0.25 (3KDM_H-3KDM_B) | 1.5 (3KDM_H) | HUMAN |
| H3-13-XIX | 2 | 1 (2%) | 2ntf_H | PPPGPBDABBPAB | 2 | 1 | 0.26 (2ntf_H-2ntf_B) | 3.18 (2ntf_H) | MOUSE |
| H3-13-XX | 2 | 1 (2%) | 2GJZ_H | PAPABABPABBAB | 2 | 1 | 0.28 (2GJZ_H-2GJZ_B) | 2.65 (2GJZ_H) | MOUSE |
| H3-13-XXI | 2 | 1 (2%) | 1fbi_H | BDAPPAAALPPBL | 2 | 1 | 0.3 (1fbi_H-1fbi_Q) | 3.0 (1fbi_H) | MOUSE |
| H3-13-XXII | 2 | 1 (2%) | 1KXV_C | BPABPBPLBPPPP | 1 | 1 | 0.33 (1KXV_C-1KXV_D) | 1.6 (1KXV_C) | CAMEL |
| **Cluster** | **Population** | **Unique sequences** | **Medoid PDB entry** | **Medoid Ramachandran conformation** | **Level-3 clusters** | **Level-2 clusters** | **Cluster diameter (Å)(Furthest members)** | **Best resolution in cluster (Å)(PDB)** | **Species in cluster** |
| ***CDR-H3 14-residues, Total population: 128, Total Unique sequences: 40, Clusters: 19, Outliers/Singletons: 24, Av.Silhouette: 0,78*** | | | | | | | | | |
| H3-14-I | 20 | 1 (3%) | 3CFK_F | BPPBAAALBBBPAB | 8 | 2 | 0.52 (3CFJ_F-1Y0L_D) | 2.5 (1Y0L_H) | HUMAN, MOUSE |
| H3-14-II | 20 | 1 (3%) | 1op3_H | PBBABPBABBPPAB | 9 | 6 | 1.58 (3OAY_H-1op5_M) | 1.75 (1op3_H) | HUMAN |
| H3-14-III | 10 | 2 (5%) | 1yec_H | ALPBAAALBBBPPB | 1 | 1 | 0.4 (1yek_H-1yee_H) | 1.85 (1yej_H) | MOUSE |
| H3-14-IV | 9 | 1 (3%) | 2WZP_L | BBLGPBAPAAAPAB | 5 | 5 | 0.93 (2WZP_F-2BSE_F) | 2.6 (2WZP_D) | LLAMA |
| H3-14-V | 6 | 1 (3%) | 1wcb_H | PPBABDBAADPLAB | 6 | 3 | 0.51 (1wcb_H-1wc7_B) | 2.3 (2bmk_B) | MOUSE |
| H3-14-VI | 4 | 1 (3%) | 1kxq_E | BDPDBAAALBLPDB | 3 | 1 | 0.14 (1kxq_H-1kxq_F) | 1.6 (1kxq_E) | CAMEL |
| H3-14-VII | 4 | 2 (5%) | 2vir_B | PBBPAADLBPBBAP | 3 | 1 | 0.53 (2vir_B-2QSC_H) | 2.8 (2QSC_H) | HUMAN, MOUSE |
| H3-14-VIII | 4 | 1 (3%) | 2dqu_H | BPBBPLPABBBPAB | 2 | 1 | 0.15 (1hyy_H-1hyx_H) | 1.7 (2dqu_H) | MOUSE |
| H3-14-IX | 4 | 2 (5%) | 1cz8_H | PPPPBGBPBPBPAB | 3 | 1 | 0.31 (1cz8_Y-1bj1_H) | 2.4 (1bj1_H) | MOUSE |
| H3-14-X | 4 | 2 (5%) | 2aj3_F | PPBBADAGPDBBAB | 4 | 2 | 1.31 (2aj3_D-1om3_K) | 2.03 (2aj3_B) | HUMAN |
| H3-14-XI | 3 | 1 (3%) | 3QHF_H | BBBPPLBPPBPLAB | 3 | 2 | 0.67 (3QHZ_I-3LZF_H) | 1.55 (3QHZ_I) | HUMAN |
| H3-14-XII | 2 | 1 (3%) | 3IYW_H | not applicable | 1 | 1 | 0.00 (3IYW_H-3N9G_H) | 1.43 (3N9G_H) | HUMAN |
| H3-14-XIII | 2 | 1 (3%) | 1uwx_H | PPBBPLPABBBBAP | 2 | 1 | 0.05 (1uwx_H-1uwx_M) | 2.2 (1uwx_H) | MOUSE |
| H3-14-XIV | 2 | 1 (3%) | 3RKD_H | BBBBPLAGLPBPAB | 2 | 1 | 0.08 (3RKD_H-3RKD_D) | 1.9 (3RKD_H) | MOUSE |
| H3-14-XV | 2 | 1 (3%) | 1fns_H | BALBAADLBDPPAB | 1 | 1 | 0.14 (1fns_H-1oak_H) | 2.0 (1fns_H) | MOUSE, RABBIT |
| H3-14-XVI | 2 | 1 (3%) | 2J4W_H | PPPAAABLDLPBAB | 2 | 1 | 0.24 (2J4W_H-2J5L_C) | 2.5 (2J4W_H) | MOUSE |
| H3-14-XVII | 2 | 1 (3%) | 3G6J_F | PBAGPBLLPBBBAB | 2 | 2 | 0.27 (3G6J_F-3G6J_H) | 3.1 (3G6J_F) | HUMAN |
| H3-14-XVIII | 2 | 1 (3%) | 3CMO_H | BPPPDGGDPBBBAB | 2 | 2 | 0.47 (3CMO_H-3CMO_Y) | 2.3 (3CMO_H) | MOUSE |
| H3-14-XIX | 2 | 1 (3%) | 2ai0_I | not applicable | 2 | 2 | 0.61 (2ai0_I-2ai0_J) | 2.2 (2ai0_I) | MOUSE |
| ***CDR-H3 15-residues, Total population: 96, Total Unique sequences: 23, Clusters: 18, Outliers/Singletons: 15, Av.Silhouette: 0,86*** | | | | | | | | | |
| H3-15-I | 16 | 1 (4%) | 3QXW_E | BABAAAAAPAAAPAB | 6 | 1 | 0.95 (3QXU_D-3QXT_B) | 1.7 (3QXT_A) | LLAMA |
| H3-15-II | 10 | 1 (4%) | 3MLS_H | PPPPPBABPBBBPAB | 8 | 1 | 0.88 (3MLU_H-3MLT_H) | 1.8 (3MLR_H) | HUMAN |
| H3-15-III | 8 | 1 (4%) | 3IKC_B | PPBADBGPBBPBPAB | 2 | 1 | 0.21 (3IKC_D-3IJY_B) | 2.1 (3IJH_B) | MOUSE |
| H3-15-IV | 6 | 1 (4%) | 3FKU_X | PAPABPDBAAPBBAB | 6 | 1 | 0.37 (3FKU_S-3FKU_Z) | 3.2 (3FKU_X) | HUMAN |
| H3-15-V | 4 | 1 (4%) | 2VYR_L | PABAAPDBPLPPPDB | 2 | 1 | 0.17 (2VYR_J-2VYR_I) | 2.0 (2VYR_I) | HUMAN |
| **Cluster** | **Population** | **Unique sequences** | **Medoid PDB entry** | **Medoid Ramachandran conformation** | **Level-3 clusters** | **Level-2 clusters** | **Cluster diameter (Å)(Furthest members)** | **Best resolution in cluster (Å)(PDB)** | **Species in cluster** |
| H3-15-VI | 4 | 1 (4%) | 3UC0_H | PPBADBGABPLBBAB | 4 | 3 | 0.45 (3UC0_I-3UAJ_C) | 2.71 (3UC0_H) | CHIMPANZEE |
| H3-15-VII | 4 | 1 (4%) | 2VYR_H | PAPADPDDBPBAAPP | 1 | 1 | 0.19 (2VYR_G-2VYR_F) | 2.0 (2VYR_E) | HUMAN |
| H3-15-VIII | 4 | 1 (4%) | 3HZM_B | BPBAAPAAPBBBBAB | 4 | 1 | 0.33 (3HZY_B-3HZK_B) | 1.8 (3HZM_B) | MOUSE |
| H3-15-IX | 4 | 1 (4%) | 3BKC_H | PPBPGDPAABBBPAB | 4 | 3 | 0.53 (3BKM_H-3BAE_H) | 1.59 (3BAE_H) | MOUSE |
| H3-15-X | 4 | 1 (4%) | 3PJS_B | BPPPDDDAAAPBPAB | 4 | 4 | 0.5 (3PJS_D-3EFF_D) | 3.8 (3EFF_B) | MOUSE |
| H3-15-XI | 3 | 1 (4%) | 2b1a_H | PBLLABABGBDBPAB | 1 | 1 | 0.22 (2b1h_H-2b0s_H) | 2.0 (2b1h_H) | HUMAN |
| H3-15-XII | 2 | 1 (4%) | 1yed_H | ALPPBAAALBBPPPB | 1 | 1 | 0.01 (1yed_B-1yed_H) | 3.1 (1yed_H) | MOUSE |
| H3-15-XIII | 2 | 1 (4%) | 2QQK_H | BBAPLBAAAPLPPAB | 2 | 1 | 0.15 (2QQK_H-2QQL_H) | 2.75 (2QQK_H) | HUMAN |
| H3-15-XIV | 2 | 1 (4%) | 3MLT_E | BBBLBGAAPPABPAP | 2 | 2 | 0.21 (3MLT_E-3MLT_I) | 2.49 (3MLT_E) | HUMAN |
| H3-15-XV | 2 | 1 (4%) | 3GI8_H | BPBBAABDPBBPPAB | 2 | 2 | 0.24 (3GI8_H-3GI9_H) | 2.48 (3GI9_H) | MOUSE |
| H3-15-XVI | 2 | 1 (4%) | 3AAZ_A | BPBBGAPAABBBPDP | 2 | 2 | 0.25 (3AAZ_A-3AAZ_H) | 2.2 (3AAZ_A) | HUMAN |
| H3-15-XVII | 2 | 1 (4%) | 1fn4_B | BBALPBAPGLAGBBL | 2 | 2 | 0.34 (1fn4_B-1fn4_D) | 2.8 (1fn4_B) | RAT |
| H3-15-XVIII | 2 | 1 (4%) | 3K80_A | BAALDGBAPAADBAB | 2 | 1 | 0.38 (3K80_A-3K80_B) | 2.4 (3K80_A) | LLAMA |
| ***CDR-H3 16-residues, Total population: 40, Total Unique sequences: 16, Clusters: 8, Outliers/Singletons: 12, Av.Silhouette: 0,81*** | | | | | | | | | |
| H3-16-I | 10 | 1 (6%) | 3dwt_H | BPBBBDBPPBAAABDP | 6 | 1 | 0.86 (3eak_B-3dwt_C) | 1.95 (3eak_A) | CAMEL |
| H3-16-II | 4 | 1 (6%) | 3CSY_G | PPAAPBADBBBBPPAB | 4 | 3 | 0.28 (3CSY_E-3CSY_C) | 3.4 (3CSY_A) | HUMAN |
| H3-16-III | 4 | 1 (6%) | 2hrp_N | PPPBPPAABBPPBPAB | 4 | 2 | 0.77 (2hrp_H-1mf2_H) | 2.2 (2hrp_H) | MOUSE |
| H3-16-IV | 2 | 1 (6%) | 1U0Q_A | BPPLDGPAAPAAAPAB | 1 | 1 | 0.09 (1U0Q_A-1U0Q_B) | 1.6 (1U0Q_A) | LLAMA |
| H3-16-V | 2 | 1 (6%) | 3INU_H | PPAAPBGDPPPBPPDB | 2 | 1 | 0.18 (3INU_H-3INU_M) | 2.5 (3INU_H) | HUMAN |
| H3-16-VI | 2 | 1 (6%) | 3U7W_H | PAPBBAABBBADPPAB | 2 | 1 | 0.32 (3U7W_H-3U7Y_H) | 2.45 (3U7Y_H) | HUMAN |
| H3-16-VII | 2 | 1 (6%) | 1etz_H | BBBBBBLLPBBBBBAP | 2 | 1 | 0.35 (1etz_H-1etz_B) | 2.6 (1etz_H) | MOUSE |
| H3-16-VIII | 2 | 1 (6%) | 3RA7_H | PABABAAPAAAABPAB | 2 | 1 | 0.36 (3RA7_H-3RA7_I) | 2.8 (3RA7_H) | MOUSE |
| ***CDR-H3 17-residues, Total population: 28, Total Unique sequences: 14, Clusters: 6, Outliers/Singletons: 9, Av.Silhouette: 0,93*** | | | | | | | | | |
| H3-17-I | 7 | 1 (7%) | 3PNW_B | BPAGDBPPPLLLLPPAB | 3 | 1 | 0.33 (3PNW_Q-3PNW_E) | 2.05 (3PNW_B) | HUMAN |
| H3-17-II | 3 | 1 (7%) | 3SM5_H | BDPPPABAABPBPPPAB | 2 | 1 | 0.02 (3SM5_J-3SM5_H) | 3.19 (3SM5_H) | HUMAN |
| H3-17-III | 3 | 1 (7%) | 1f58_H | BBPBPBGBLBBBPBPDB | 3 | 1 | 0.56 (3f58_H-2f58_H) | 2.0 (1f58_H) | MOUSE |
| **Cluster** | **Population** | **Unique sequences** | **Medoid PDB entry** | **Medoid Ramachandran conformation** | **Level-3 clusters** | **Level-2 clusters** | **Cluster diameter (Å)(Furthest members)** | **Best resolution in cluster (Å)(PDB)** | **Species in cluster** |
| H3-17-IV | 2 | 1 (7%) | 1mvf_A | BABPPABPADBAAABDB | 1 | 1 | 0.21 (1mvf_A-1mvf_B) | 1.65 (1mvf_A) | CAMEL |
| H3-17-V | 2 | 1 (7%) | 2fb4_H | PAPDLBBBAABBAGPDB | 2 | 1 | 0.22 (2fb4_H-2ig2_H) | 1.9 (2fb4_H) | HUMAN |
| H3-17-VI | 2 | 1 (7%) | 3MLW_H | PBBBPAALPBBDDBPAP | 2 | 1 | 0.23 (3MLW_H-3MLW_I) | 2.7 (3MLW_H) | HUMAN |
| ***CDR-H3 18-residues, Total population: 37, Total Unique sequences: 11, Clusters: 6, Outliers/Singletons: 6, Av.Silhouette: 0,82*** | | | | | | | | | |
| H3-18-I | 12 | 2 (18%) | 3JUY_F | PPPPPPLPPPLADAPPAB | 10 | 3 | 1.35 (3JUY_D-1n0x_K) | 1.8 (1n0x_H) | HUMAN |
| H3-18-II | 9 | 1 (9%) | 2fx9_I | PPBPGPGDDPPPDGPPAB | 4 | 3 | 0.89 (2fx8_K-2fx8_I) | 1.76 (2fx7_H) | HUMAN |
| H3-18-III | 4 | 1 (9%) | 2X1P_C | BAABADPPPPAPAAABDB | 1 | 1 | 0.19 (2X1P_D-2X1P_B) | 1.1 (2X1P_A) | LLAMA |
| H3-18-IV | 2 | 1 (9%) | 2a9n_H | BPPPAPGBPLDDBABBDB | 2 | 2 | 0.32 (2a9n_H-2a9n_I) | 3.0 (2a9n_H) | HUMAN |
| H3-18-V | 2 | 1 (9%) | 2a9m_H | PPAADDBADAAAAPBBAB | 2 | 1 | 0.54 (2a9m_I-2a9m_H) | 2.1 (2a9m_H) | HUMAN |
| H3-18-VI | 2 | 1 (9%) | 3STB_A | BAPBPABPBPPPAAAPAB | 2 | 1 | 0.56 (3STB_A-3STB_B) | 2.5 (3STB_A) | LLAMA |
| ***CDR-H3 19-residues, Total population: 48, Total Unique sequences: 12, Clusters: 9, Outliers/Singletons: 2, Av.Silhouette: 0,89*** | | | | | | | | | |
| H3-19-I | 24 | 2 (17%) | 2NY3_D | BPDGPAADLPBALPAPBAB | 6 | 2 | 0.42 (1rzk_H-1gc1_H) | 1.99 (2NY1_D) | HUMAN |
| H3-19-II | 5 | 1 (8%) | 3MLY_I | BBBBBBDALAGPBPDBBAB | 5 | 4 | 0.54 (3MLY_H-3MLX_I) | 1.7 (3MLY_H) | HUMAN |
| H3-19-III | 3 | 1 (8%) | 2X89_C | BPAAAAADPPAPLPDAPAB | 1 | 1 | 0.19 (2X89_B-2X89_A) | 2.16 (2X89_A) | HUMAN |
| H3-19-IV | 3 | 1 (8%) | 3IDX_H | PPPPPAAPDBPBABDGPDB | 3 | 2 | 1.06 (3IDY_B-3IDY_H) | 2.5 (3IDX_H) | HUMAN |
| H3-19-V | 3 | 1 (8%) | 1KXT_D | GPGBBBBBBLLBBBPBAGP | 3 | 2 | 0.28 (1KXT_F-1KXT_B) | 2.0 (1KXT_B) | CAMEL |
| H3-19-VI | 2 | 1 (8%) | 3BN9_D | PPBABAABPBPDADBPBAB | 1 | 1 | 0.12 (3BN9_D-3BN9_F) | 2.17 (3BN9_D) | HUMAN |
| H3-19-VII | 2 | 1 (8%) | 1f2x_K | PDBDPAAAAAALBAAAPAP | 2 | 1 | 0.17 (1f2x_K-1f2x_L) | 2.1 (1f2x_K) | CAMEL |
| H3-19-VIII | 2 | 1 (8%) | 3SKJ_H | BBAADBPBABPPAPBBBAB | 2 | 1 | 0.47 (3SKJ_H-3SKJ_I) | 2.5 (3SKJ_H) | HUMAN |
| H3-19-IX | 2 | 1 (8%) | 3TV3_H | BBGPDABBBGBPBPDBBAB | 2 | 2 | 0.41 (3TV3_H-3TYG_H) | 1.29 (3TV3_H) | HUMAN |
| ***CDR-H3 20-residues, Total population: 13, Total Unique sequences: 4, Clusters: 3, Outliers/Singletons: 0, Av.Silhouette: 0,90*** | | | | | | | | | |
| H3-20-I | 7 | 2 (50%) | 3GHB_H | BBBBBBPPADBPBBBPBPAP | 6 | 2 | 1.34 (3GHE_H-3C2A_I) | 2.1 (3C2A_H) | HUMAN |
| H3-20-II | 4 | 1 (1%) | 3ZTJ_I | PAPPLPBPABADPPPBPPAB | 4 | 1 | 0.33 (3ZTN_H-3ZTJ_G) | 3.0 (3ZTN_H) | HUMAN |
| H3-20-III | 2 | 1 (1%) | 1za3_B | BPBDAAAAAALPPBDLBPDB | 2 | 1 | 0.25 (1za3_B-1za3_H) | 3.35 (1za3_B) | HUMAN |
| ***CDR-H3 21-residues, Total population: 10, Total Unique sequences: 1, Clusters: 1, Outliers/Singletons: 0, Av.Silhouette: N/A*** | | | | | | | | | |
| H3-21-I | 10 | 1 (100%) | 3Q6F_B | BALPBBBBADAGPBBBPBPDB | 5 | 1 | 1.19 (3Q6F_H-3PIQ_G) | 3.19 (3Q6F_B) | HUMAN |
| **Cluster** | **Population** | **Unique sequences** | **Medoid PDB entry** | **Medoid Ramachandran conformation** | **Level-3 clusters** | **Level-2 clusters** | **Cluster diameter (Å)(Furthest members)** | **Best resolution in cluster (Å)(PDB)** | **Species in cluster** |
| ***CDR-H3 22-residues, Total population: 33, Total Unique sequences: 4, Clusters: 2, Outliers/Singletons: 2, Av.Silhouette: 0,90*** | | | | | | | | | |
| H3-22-I | 29 | 1 (25%) | 1u92_B | BPPPPBBLLBPDBAAAAPBBAB | 7 | 2 | 1.29 (3LEV_H-1tjh_H) | 1.86 (3IDG_B) | HUMAN, MOUSE |
| H3-22-II | 2 | 1 (25%) | 1rhh_B | BBBPDLLBABDBLBBPAABBAB | 2 | 1 | 0.44 (1rhh_B-1rhh_D) | 1.9 (1rhh_B) | HUMAN |
| ***CDR-H3 23-residues, Total population: 1, Total Unique sequences: 1, Clusters: 0, Outliers/Singletons: 1, Av.Silhouette: N/A*** | | | | | | | | | |
| H3-23-O-1* | 1 | 1 (100%) | 3G9A_B | BDPPBDLPBGPDGABAAAPPBAB | 1 | 1 | N/A | 1.61 (3G9A_B) | ALPACA |
| ***CDR-H3 24-residues, Total population: 12, Total Unique sequences: 2, Clusters: 2, Outliers/Singletons: 0, Av.Silhouette: 0,95*** | | | | | | | | | |
| H3-24-I | 7 | 1 (50%) | 1jtp_A | BAPPPAPPBPAAAAAABLLPLPAB | 7 | 2 | 0.58 (1mel_A-1jtt_A) | 1.9 (1jtp_A) | CAMEL |
| H3-24-II | 5 | 1 (50%) | 3U46_A | PPDBBBPAALPPBPLPPBDABBAB | 4 | 1 | 0.38 (3U4B_H-3TCL_A) | 1.91 (3TCL_H) | HUMAN |
| ***CDR-H3 25-residues, Total population: 1, Total Unique sequences: 1, Clusters: 0, Outliers/Singletons: 1, Av.Silhouette: N/A*** | | | | | | | | | |
| H3-25-O-1* | 1 | 1 (100%) | 3Q6G_H | BPBBPBPBPPABBPBBBBBPBPPBL | 1 | 1 | N/A | 1.61 (3Q6G_H) | HUMAN |
| ***CDR-H3 28-residues, Total population: 12, Total Unique sequences: 2, Clusters: 1, Outliers/Singletons: 0, Av.Silhouette: N/A*** | | | | | | | | | |
| H3-28-I | 12 | 2 (100%) | 3MUG_J | PPBBPPPBLLBPBBPPLLPPPPALBBAB | 10 | 2 | 1.44 (3U4E_A-3MUG_L) | 1.8 (3U2S_H) | HUMAN |
| ***CDR-H3 31-residues, Total population: 1, Total Unique sequences: 1, Clusters: 0, Outliers/Singletons: 1, Av.Silhouette: N/A*** | | | | | | | | | |
| H3-31-O-1* | 1 | 1 (100%) | 3U1S_H | BBBBBBPABBBPBADBPPPBBBBBBPABPDP | 1 | 1 | N/A | 2.3 (3U1S_H) | HUMAN |
| **Total (level-1 clusters only)** | **1620** |  |  |  | **851** | **411** |  |  |  |

**Supplementary table:** Summary for the clustering of CDR-H3. Level-2 clusters are shown exceptionally (marked with an asterisk) when no level-1 cluster is formed (minimum of 2 members required).
